# Mass spectrometry-complemented molecular modeling predicts the interaction interface for a camelid single-domain antibody targeting the *Plasmodium falciparum* circumsporozoite protein’s C-terminal domain

**DOI:** 10.1016/j.csbj.2024.08.023

**Published:** 2024-08-28

**Authors:** Kwabena F.M. Opuni, Manuela Ruß, Rob Geens, Line De Vocht, Pieter Van Wielendaele, Christophe Debuy, Yann G.-J. Sterckx, Michael O. Glocker

**Affiliations:** a Department of Pharmaceutical Chemistry, School of Pharmacy, College of Health Science, University of Ghana, P.O. Box LG43, Legon, Ghana; b Proteome Center Rostock, University Medicine Rostock and University of Rostock, Schillingallee 69, 18057 Rostock, Germany; c Laboratory of Medical Biochemistry, Faculty of Pharmaceutical, Biomedical, and Veterinary Sciences, University of Antwerp, Universiteitsplein 1, Wilrijk, 2610 Antwerp, Belgium

**Keywords:** AlphaFold2, Assembled epitope, Circumsporozoite protein, Epitope mapping, *in-silico* docking, ITEM-TWO analysis, Mass spectrometry, Paratope mapping, *Plasmodium falciparum*

## Abstract

**Background:**

Bioanalytical methods that enable rapid and high-detail characterization of binding specificities and strengths of protein complexes with low sample consumption are highly desired. The interaction between a camelid single domain antibody (sdAbCSP1) and its target antigen (PfCSP-Cext) was selected as a model system to provide proof-of-principle for the here described methodology.

**Research design and methods:**

The structure of the sdAbCSP1 – PfCSP-Cext complex was modeled using AlphaFold2. The recombinantly expressed proteins, sdAbCSP1, PfCSP-Cext, and the sdAbCSP1 – PfCSP-Cext complex, were subjected to limited proteolysis and mass spectrometric peptide analysis. ITEM MS (Intact Transition Epitope Mapping Mass Spectrometry) and ITC (Isothermal Titration Calorimetry) were applied to determine stoichiometry and binding strength.

**Results:**

The paratope of sdAbCSP1 mainly consists of its CDR3 (aa100–118). PfCSP-Cext’s epitope is assembled from its α-helix (aa40–52) and opposing loop (aa83–90). PfCSP-Cext’s GluC cleavage sites E46 and E58 were shielded by complex formation, confirming the predicted epitope. Likewise, sdAbCSP1’s tryptic cleavage sites R105 and R108 were shielded by complex formation, confirming the predicted paratope. ITEM MS determined the 1:1 stoichiometry and the high complex binding strength, exemplified by the gas phase dissociation reaction enthalpy of 50.2 kJ/mol. The *in-solution* complex dissociation constant is 5 × 10^-10^ M.

**Conclusions:**

Combining AlphaFold2 modeling with mass spectrometry/limited proteolysis generated a trustworthy model for the sdAbCSP1 – PfCSP-Cext complex interaction interface.

## Graphical Abstract

**Figure:**
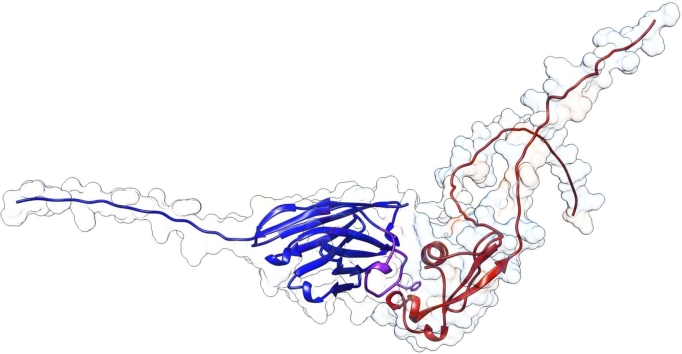

## 1 Introduction

Camelid single domain antibodies (sdAbs aka nanobodies) correspond to the variable domain (VHH) of heavy-chain only Abs (HCAbs) found in llamas, alpacas, camels, and dromedaries [1], [2]. Since the generation of sdAbs has become routine, they are of interest beyond immuno-analytical or diagnostic purposes [3]. Envisaged biomedical application areas include tumor treatment [4], therapeutics against human viral diseases [5], and treatment of infectious diseases in general [6]. Their small sizes, together with their capabilities to bind epitopes that may not be targetable by conventional antibodies, make sdAbs attractive for being widely applied in research and application development. Considering that highly specific and irreversible antigen binding remains any binder’s most important feature and considering that sequence characteristics of binders have not yet become completely predictable [7], determinations of antigen specificities and of binding constants by experiment keep their importance. Methods that report important molecular features of binders, specificity and binding strength, at best without the need for time- and large material amount-consuming procedures, are highly desired.

Regarded as a real breakthrough in molecular modeling, the potential of machine learning-based methods (e.g., AlphaFold and RoseTTAFold) has been proven for generating accurate tertiary structure predictions with just the amino acid sequences as input, but without the need for any further experimental data [8]. Yet, while extremely successful in predicting secondary structure details, predicting the overall domain orientation of multi-domain proteins remains a challenge for AlphaFold2 [9], [10]. This limitation affects structure predictions of protein complexes, particularly with respect to assigning the arrangements of the interacting proteins with respect to each other [11]. A solution to this problem has been suggested by combining AlphaFold2 predictions with experimental data, which might come either from macromolecular X-ray crystallography (MX) [12], nuclear magnetic resonance (NMR) [13], or chemical / photo-cross-linking (XL) combined with mass spectrometry (MS) [14], [15], [16], [17] and hydrogen - deuterium exchange (HDX) MS [18]. Then, assigning interacting partial surfaces of complexes with atomic resolution through integrative approaches is thought to be within reach, even without knowledge of 3D coordinates of atoms obtained by experiment.

MS-based methods have been developed for experimentally determining binding regions on an antigen’s surface, i.e*.*, for identifying the epitope, which is of high interest because of the mass spectrometer’s known precision and, perhaps equally important, its very low sample consumption [19]. The hitherto most often applied MS-based epitope mapping methods, epitope extraction, and epitope excision [20], [21], include limited proteolysis of antigens with more or less sequence-specific proteases [22], [23], [24], [25]. Comparison of antigen-derived digestion products, obtained with or without the presence of an antibody (or fragment thereof), has been informative by revealing those cleavage sites that had been protected through complex formation, enabling the identification of those peptides of a protein’s partial surface area that contained parts of or even the entire epitope [26]. In a more recent refinement of the MS-based epitope mapping method, termed “Intact Transition Epitope Mapping (ITEM)”, immobilization of one of the complex forming partners, as well as the need for chemical labeling of either the antigen or the antibody, has become obsolete. Because of the ultra-high sensitivity of modern mass spectrometers, sample consumption was even lowered further to low pmol or high fmol quantities [27], [28], [29]. The combination of *in-silico* computational methods, which are used for structure modeling and molecular dynamics simulation with MS-based epitope mapping approaches, allowed an understanding of force-interchanging amino acid residues on the sub-epitope level, i.e*.*, on the level of individual amino acid residues within a given epitope [30]. Moreover, by applying the ITEM approach, precise estimation of binding strength differences became possible, which were connected to (i) single amino acid exchanges [31], [32], (ii) post-translational modifications at specific amino acid positions [33], or (iii) influences of intrinsically disordered regions (IDRs) on protein complex stability [34].

Here, we describe a structural biology approach to determine the molecular interface, binding stoichiometry, and binding strength between a camelid sdAb (sdAbCSP1) and its target antigen (the C-terminal domain of the *Plasmodium falciparum* circumsporozoite protein, PfCSP-Cext) which was selected as a model system. Structure prediction of the sdAbCSP1 – PfCSP-Cext complex was initiated with AlphaFold2, followed by scoring the resulting complex models using HADDOCK 2.4. The *in-silico* predicted interaction sites were confirmed by experimental mapping of both epitope and paratope using limited proteolysis of PfCSP-Cext, sdAbCSP1, and the sdAbCSP1 – PfCSP-Cext complex combined with MS as readout. We also determined the binding stoichiometry and the binding strength of the sdAbCSP1 – PfCSP-Cext complex by ITEM MS and compared this to isothermal titration calorimetry (ITC) results. Combining all the data provided an accurate interface picture for the sdAbCSP1 – PfCSP-Cext complex. Because of its speed and low sample consumption, the approach reported here (combining *in silico*, in vitro, and gas-phase-depending methods) may be suggested to serve as a *comme-il-faut* workflow for investigating similar complexes.

## 2 Materials and methods

### 2.1 Modeling of protein and protein complex structures

#### 2.1.1 Modeling of the 3D structure of sdAbCSP1 and PfCSP-Cext

The amino acid sequences of sdAbCSP1 (Fig. S1A) and of PfCSP-Cext (Fig. S1B) were used to model the sdAbCSP1 and the PfCSP-Cext 3D structures using AlphaFold2 [35]. The AlphaFold2 server (https://colab.research.google.com/github/sokrypton/ColabFold/blob/main/AlphaFold2.ipynb#scrollTo=G4yBrceuFbf3, accessed on 31/01/2024) generated PDB files of 5 sdAbCSP1 candidate structures as well as of 5 PfCSP-Cext candidate structures and ranked them based on the pLDDT scores. The protein structures with the highest pLDDT scores were selected and pdb files were generated.

#### 2.1.2 Modeling of the 3D structure of sdAbCSP1 – PfCSP-Cext and sdAbCSP1 – PfCSP-C (tr) complexes

The structural models of sdAbCSP1 – PfCSP-Cext and sdAbCSP1 – PfCSP-C (tr) complexes were predicted using AlphaFold-Multimer [36] as well as the AlphaFold2 server upon submitting the amino acid sequences of sdAbCSP1 and PfCSP-Cext or PfCSP-C (tr). Twenty-five models were predicted per run, and the best models underwent a final relaxation step. The models were evaluated based on the following parameters: AlphaFold-Multimer model confidence (a weighted combination of the predicted template modeling (pTM) and interface predicted template modeling (ipTM) scores, 0.8 *ipTM + 0.2 *pTM [36], the predicted aligned error (PAE) matrix [8], the local and global predicted local distance difference test (pLDDT) scores [8], the predicted DockQ (pDockQ) values [11] and the normalized discrete optimized protein energy (zDOPE) scores [37]. Protein-protein interactions were analyzed using the PISA server [38]. Molecular graphics and analyzes were performed with UCSF ChimeraX [39]. A distance of ≤ 4 Å was chosen to determine the amino acid residues of the PfCSP-Cext epitope, which made contact with the sdAbCSP1 paratope and vice versa [39].

#### 2.1.3 HADDOCK docking and scoring

The protein and / or complex model structures obtained from AlphaFold2 modeling, as well as complexes from the PDB files 1BZQ [40] and 5USF [41], were submitted to HADDOCK 2.4 (Utrecht, The Netherlands) docking experiments [42]. The protein coordinates of the complexes from AlphaFold2 output files, as well as those of the complexes from the PDB data bank files, were separated and saved as independent protein PDB files. These individual protein PDB files were used as input files for docking using HADDOCK 2.4. “Default” configurations are the same configurations as from the AlphaFold2 output files. “Crystal” configurations (positive controls) are the same configurations as from the PDB files 1BZQ [40] and 5USF [41]. HADDOCK 2.4 clustered up to 200 structures into 1 cluster, representing 100 % of the water-refined models. “Head on”, “side on”, “backward” and “random” docking configurations (negative controls) were manually assembled prior to performing an *in silico* docking using amino acid residues 46–58 from the PfCSP-Cext antigen (which encompasses most of the alpha helix (aa40–52)) and amino acids of sdAbCSP1 CDR1, CDR2, and CDR3 configuration; amino acid residues 46–58 from the PfCSP-Cext antigen (which encompasses most of the alpha helix (aa40–52)) and amino acids of sdAbCSP1 CDR3 configuration; and not indicating any configuration, respectively.

### 2.2 SASA calculations

The SASAs of the epitope and paratope of the sdAbCSP1 – PfCSP-Cext complex were calculated using an in-house bioinformatics tool, “EpiMED-Surf” [43], [44]. The PDB files of the individual protein models and / or the complex models were used as input files and the SASAs for each atom, the hydrophobicity of each residue, and the per-atom charge and radius were computed. The output PDB file of the complex model after the SASA calculation was edited to obtain PDB files for the separated PfCSP-Cext and sdAbCSP1 models. The differences of the SASAs between (i) sdAbCSP1 – PfCSP-Cext complex and (ii) PfCSP-Cext alone were calculated for those amino acid residues which were determined to be in close vicinities (distance of ≤ 4 Å), and the differences in SASA values of each atom of the epitope were determined. Next, the differences of the SASAs between (i) sdAbCSP1 – PfCSP-Cext complex and (ii) sdAbCSP1 alone were calculated, and the differences in SASA values of each atom of the paratope were determined.

### 2.3 Binding strength variations upon *in silico* site-specific mutagenesis

The changes in binding energies of the sdAbCSP1 – PfCSP-Cext complex upon introducing point mutations in the epitope - paratope binding interface were predicted using the BeAtMuSiC web server (version 1.0, Brussels, Belgium) [30], [45], [46] (accessed on 20/03/2024). The PDB file of the sdAbCSP1 – PfCSP-Cext complex model was uploaded to the web server, and the amino acid residues of PfCSP-Cext and sdAbCSP1 of the complex model were selected as the two binding partners. The server automatically performed systematic mutations of the interface amino acid residues, i.e*.,* residues belonging either to the epitope or to the paratope.

### 2.4 Protein stock solutions and buffers

sdAbCSP1 (1.6 µg/µl) was dissolved in 20 mM Tris-HCl, 150 mM NaCl, pH 7.5 (stock solution 1). PfCSP-Cext (1.96 µg/µl) was dissolved in 50 mM Tris-HCl, 500 mM NaCl, pH 7.5 (stock solution 2). Protein stock solutions 1 and 2 were stored frozen at −20 °C prior to further handling. 200 mM ammonium acetate, pH 6.7, was used for rebuffering proteins from stock solutions 1 and 2. Endoproteinase Glu-C was purchased from Roche (Basel, Switzerland). An amount of 50 µg of Glu-C (1 vial) was dissolved in 100 µl deionized water to reach a final protease concentration of 500 ng/µl (stock solution 3). Sequencing grade modified trypsin was purchased from Promega (Madison, Wisconsin, USA). An amount of 20 µg of trypsin (1 vial) was dissolved in 200 µl 50 mM ammonium bicarbonate, pH 7.8, to reach a final protease concentration of 100 ng/µl (stock solution 4). Protease stock solutions 3 and 4 were stored at −20 °C prior to further handling.

### 2.5 Preparation of protein working solutions

To prepare sdAbCSP1 and PfCSP-Cext working solutions 1 and 2, stock solutions 1 and 2, respectively, were rebuffered into 200 mM ammonium acetate, pH 6.7, using Amicon Ultra 0.5 centrifugal 3 K filter devices (Merck Millipore, Carrigtwohill, Ireland) for several times. In each case, 100 µl aliquots of protein stock solutions 1 and 2, respectively, were placed in separate filter devices which were filled up with 400 µl 200 mM ammonium acetate, pH 6.7, each. Centrifugations were performed at 13,400 rpm for 30 min using a table centrifuge (MiniSpin, Eppendorf, Hamburg, Germany) at room temperature. The flow-through fractions were discarded and the filter devices were filled up with 400 µl of 200 mM ammonium acetate, pH 6.7, each. Centrifugation / discarding / re-filling steps were repeated eight times, each. Subsequently, the filtration devices were placed upside down on top of new tubes and were centrifuged for 5 min at 4500 rpm. Finally, the sdAbCSP1 retentates (ca. 50 µl, working solutions 1) or the PfCSP-Cext retentates (ca. 50 µl, working solutions 2) were separately collected. Prior to performing protein digestions, 5 µl of Glu-C solution (stock solution 3) were diluted with 45 µl of 200 mM ammonium acetate, pH 6.7, to reach a final protease concentration of 50 ng/µl (working solution 3). To prepare working solutions which contained the sdAbCSP1 – PfCSP-Cext complex with sdAbCSP1 in excess (working solution 4), 5.38 µl (10 µg) of sdAbCSP1 solution (working solution 1) were mixed with 1.87 µl (5 µg) of PfCSP-Cext solution (working solution 2). Working solution 4 was incubated for 1 h at room temperature or 16 h at 4 °C. Likewise, to prepare working solutions which contained the sdAbCSP1 – PfCSP-Cext complex with PfCSP-Cext in excess (working solution 5), 2 µl of sdAbCSP1 solution (working solution 1) and 4 µl of PfCSP-Cext (working solution 2) were diluted to final protein concentrations of 0.1 µg/µl, each, using 200 mM ammonium acetate, pH 6.7. Then, 4 µl of diluted sdAbCSP1 working solution 1 were mixed with 12 µl of diluted PfCSP-Cext working solution 2. The resulting working solution 5 was incubated for 1 h at room temperature.

### 2.6 Protein concentration determination

Protein concentrations were determined using the Qubit^TM^ 2.0 Fluorometer assay (Invitrogen by Life technologies / Thermo Fisher Scientific, Waltham, MA, USA) as described [47]. The protein concentrations of the sdAbCSP1 solutions (working solutions 1) were 1.86 µg/µl, 1.69 µg/µl, and 1.28 µg/µl. The protein concentrations of the PfCSP-Cext solutions (working solutions 2) were 2.67 µg/µl, 2.82 µg/µl, and 3 µg/µl.

### 2.7 Limited proteolysis for epitope and paratope mapping

#### 2.7.1 Limited digestion with Glu-C

A volume of 1.87 µl (5 µg) of PfCSP-Cext solution (working solution 2) was mixed with 2 µl (100 ng) Glu-C solution (working solution 3). Then, 6.13 µl of 200 mM ammonium acetate, pH 6.7, were added to reach a final volume of 10 µl. Enzyme: substrate ratio was 1:50. Digestion was performed at 25 °C for 24 h. Afterwards, an aliquot of 1 µl of the resulting peptide solution was diluted with 3 µl of 200 mM ammonium acetate, pH 6.7 (solution MS 1). A volume of 7.25 µl (15 µg) of sdAbCSP1 – PfCSP-Cext complex-containing solution (working solution 5) was mixed with 6 µl (300 ng) of Glu-C solution (working solution 3) and 1.75 µl of 200 mM ammonium acetate, pH 6.7, were added to reach a final volume of 15 µl. Enzyme: substrate ratio was 1:50. After incubation at 25 °C for 24 h, an aliquot of 2 µl of the resulting peptide-containing solution was extracted and diluted with 2 µl of 200 mM ammonium acetate, pH 6.7 (solution MS 2). A volume of 1.77 µl (5 µg) of PfCSP-Cext solution (working solution 2) was mixed with 2 µl (100 ng) of Glu-C solution (working solution 3) and 16.23 µl of 200 mM ammonium acetate, pH 6.7, were added to reach a final volume of 20 µl. Enzyme: substrate ratio was 1:50. After incubation at 25 °C for 72 h, 2 µl of 10 % formic acid, pH 1.7, were added (solution MS 3). A volume of 7.7 µl (15 µg) of sdAbCSP1 – PfCSP-Cext complex-containing solution (working solution 5) was mixed with 6 µl (300 ng) of Glu-C solution (working solution 3) and 6.3 µl of 200 mM ammonium acetate, pH 6.7, were added to reach a final volume of 20 µl. Enzyme: substrate ratio was 1:50. After incubation at 25 °C for 72 h, 2 µl of 10 % formic acid, pH 1.7 (solution MS 4).

#### 2.7.2 Limited digestion with trypsin

A volume of 2.7 µl (5 µg) of sdAbCSP1 solution (working solution 1) was mixed with 1 µl (100 ng) of trypsin (stock solution 4). Then, 6.3 µl of 200 mM ammonium acetate, pH 6.7, were added to reach a final volume of 10 µl. Enzyme: substrate ratio was 1:50. After incubation at 25 °C for 2 h, a first aliquot of 2 µl of the resulting peptide-containing solution was extracted and diluted with 3.5 µl of 200 mM ammonium acetate, pH 6.7 (solution MS 5). The tryptic digestion of the remaining peptide solution (8 µl) was continued at 25 °C for 72 h. Then, a second aliquot of 2 µl of the peptide-containing solution was extracted and diluted with 3.5 µl of 200 mM ammonium acetate, pH 6.7 (solution MS 6). A volume of 7.25 µl (15 µg) of sdAbCSP1 – PfCSP-Cext complex-containing solution (working solution 5) was mixed with 3 µl (300 ng) of trypsin solution (stock solution 4) and 4.75 µl of 200 mM ammonium acetate, pH 6.7, were added to reach a final volume of 15 µl. Enzyme: substrate ratio was 1:50. After incubation at 25 °C for 2 h, an aliquot of 2 µl of the resulting peptide-containing solution was extracted and diluted with 2 µl of 200 mM ammonium acetate, pH 6.7 (solution MS 7). The tryptic digestion of the remaining peptide solution (13 µl) was continued at 25 °C for 72 h. Then, a second aliquot of 2 µl of the peptide-containing solution was extracted and diluted with 2 µl of 200 mM ammonium acetate, pH 6.7 (solution MS 8).

### 2.8 Offline nanoESI MS^E^ peptide mapping

For offline nanoESI mass spectrometric peptide mapping analysis, 3 µl of diluted peptide mixtures of solutions MS 1, MS 2, MS 5, MS 6, MS 7, and MS 8 were loaded into separate nanoESI capillary needles. Capillary needles were pulled and gold-coated in house [30]. NanoESI-MS measurements [28] were performed on a Synapt G2-S mass spectrometer (Waters MS-Technologies, Manchester, United Kingdom) with the following measurement settings: capillary voltage, 1.0–1.2 kV; source temperature, 40 °C; source offset voltage, 30 V or 50 V; sample cone voltage, 30 V or 50 V; cone gas flow, 10 l/h; purge gas flow 20 l/h; trap gas flow, 2.0 ml/min; initial trap collision cell voltage, 4 V, and transfer collision cell voltage, 0 V. Measurements were acquired in positive-ion mode, applying a mass window of *m/z* 200–5000. The quadrupole mass filter was set to full transmission. The mass axis was calibrated using 1 mg/ml sodium iodide dissolved in isopropanol/water (50:50, v/v). Mass spectra were processed using MassLynx version 4.1 (Waters Corporation, Manchester, UK). Peptide ion signals were assigned and interpreted manually [48], comparing experimental *m/z* values with a peak list obtained from the theoretical digests of the amino acid sequences of PfCSP Cext and sdAbCSP1, respectively, using the GPMAW software 10.30 (Lighthouse data, Odense, Denmark).

### 2.9 Online nanoLC MS^E^ analysis of peptide mixtures

Nanoscale LC separations were performed with a nanoACQUITY UPLC system (Waters Corporation, Manchester, UK), equipped with a C_18_ nanoACQUITY Trap 100 Å 5 µm, 180 µm x 20 mm pre-column (Waters Corporation) and a nanoACQUITY UPLC HSS T3, 10 K psi, 100 Å, 1.8 µm, 75 µm X 150 analytical reversed phase column (Waters Corporation). Peptide mixtures of solutions MS 3 and MS 4, respectively, were diluted 1: 50 with a solution of 0.1 % formic acid / 2 % acetonitrile. The diluted peptide mixtures, 1.5 µl partial loop injection, were initially transferred with an aqueous 0.1 % formic acid / 0.1 % acetonitrile solution to the pre-column at a flow rate of 10 µl/min for 4 min. Mobile phase A was 0.1 % formic acid in water, whereas mobile phase B was 0.1 % formic acid in acetonitrile. After desalting and pre-concentration, the peptides were eluted from the pre-column to the analytical column and separated with a gradient of 3 % to 35 % mobile phase B within 55 min at a flow rate of 0.3 µl/min, followed by a 5 min rinse with 85 % of mobile phase B and a 4 min rinse with 95 % of mobile phase B. The column was re-equilibrated to achieve initial conditions for 25 min. The column temperature was maintained at 35 °C. The lock mass compound, [Glu1]-Fibrinopeptide B, was delivered by the auxiliary pump of the LC system at 0.5 µl/min at a concentration of 100 fmol/µl to the reference sprayer of the NanoLockSpray source of the mass spectrometer. The precursor ion masses and associated fragment ion spectra of the peptides that eluted from the nanoLC were measured with a SYNAPT G2S HDMS mass spectrometer (Waters Corporation, Manchester, UK), which was directly coupled to the chromatographic system. The mass spectrometer was operated in positive ion mode as described elsewhere [49]. The time-of-flight analyzer of the mass spectrometer was externally calibrated with fragment ions of [Glu1]-Fibrinopeptide B from *m/z* 50 to 2000, with the data post-acquisition lock mass corrected using the monoisotopic mass of the doubly charged precursor of [Glu1]-Fibrinopeptide B. Accurate mass data were collected in data independent acquisition mode by alternating the energy applied to the collision cell between low energy and elevated energy states. The spectral acquisition time in each mode was 0.5 s with a 0.015 s inter-scan delay. In low energy MS mode, data were collected at a constant trap collision energy of 4 V and transfer collision energy of 2 V. In elevated energy MS mode, the transfer collision energy maintained 2 V, while the trap collision energy was ramped from 18 V to 40 V within 0.5 s. One cycle of low and elevated energy data was acquired every 0.515 s. The reference sprayer was sampled every 45 s. NanoLC-MS data were processed using MassLynx version 4.1 (Waters Corporation, Manchester, UK). Peptide ion signals were assigned and interpreted manually as published [50], comparing experimental *m/z* values with a peak list obtained from the theoretical digests of the amino acid sequences of sdAbCSP1 and PfCSP Cext, respectively, using the GPMAW software 10.30 (Lighthouse data, Odense, Denmark).

### 2.10 ITEM-TWO analysis of sdAbCSP1 – PfCSP-Cext complex dissociation

A volume of 4 µl of working solution 5 was loaded into a nanoESI capillary needle. For ITEM measurements of sdAbCSP1 – PfCSP-Cext complex dissociation reactions in the gas phase, the following instrumental settings were used: capillary voltage, 1.2–1.3 kV; source temperature, 40 °C; source offset voltage, 80 V; sample cone voltage, 80 V; cone gas flow, 10 l/h; purge gas flow 20 l/h; trap gas flow, 3.0 ml/min; transfer collision cell voltage, 0 V. The first collision cell (TRAP) was used to dissociate complexes by increasing the collision cell voltage difference in a stepwise manner: 2 V, 5 V, 10 V, 15 V, 20 V, 25 V, 30 V, 35 V, 40 V, 45 V, 50 V, 55 V, 60 V, 65 V, 70 V, 75 V, 80 V, 85 V, 90 V, 95 V, 100, V, 105 V, 110 V, 115 V and 120 V. The quadrupole analyzer was used with the following settings: M1 = 3500, dwell time and ramp time 25 %; M2 = 3500, dwell time and ramp time 25 %; M3 = 3500. Measurements were acquired in positive-ion mode, applying a mass window of *m/z* 400–8000. Mass ranges were calibrated using 1 mg/ml sodium iodide in isopropanol/water (50:50, v/v). Measurements were done in triplicate and data analysis was performed as described [51], [52], [53].

The mass spectrometry raw data have been deposited at the PRIDE [54] partner repository of the ProteomeXchange Consortium with the dataset identifier PXD051302.

### 2.11 ITC analysis of sdAbCSP1 – PfCSP-Cext complex formation

The interaction between PfCSP-C and sdAbCSP1 was investigated by ITC on a MicroCal PEAQ-ITC instrument (Malvern Panalytical Ltd, Malvern, UK). SdAbCSP1 (45 µM) was titrated into the sample cell containing PfCSP-C (4 µM). Both proteins were extensively dialyzed against the same buffer (20 mM Tris-HCl, 150 mM NaCl, pH 8.0) to match the buffer composition exactly. Before being examined in the calorimeter, all samples were degassed for 10 min at a temperature close to the titration temperature (25 ℃) to prevent long equilibration delays. The reference power was set to 2 µcal s^-1^ and a stirring speed of 750 rpm was used. An equilibrium delay of 180 s before the start of each measurement was employed, while a spacing of 150 s between each injection was used. Fifteen injections with constant volumes (2.4 µl) were performed during data collection. The ﬁrst injection was always 0.4 µl and its associated heat was never considered during data analysis. To determine the injection heats, control titrations were performed consisting of sdAbCSP1 injections into the buffer-ﬁlled cell (thus, in the absence of PfCSP-C). Baseline adjustment, control subtraction, and data analysis were performed using NITPIC [55]. The data were analyzed with the “one set of sites” binding model resulting in ﬁtted values for the stoichiometry of the interaction (N), the association constant (K_a_), and the change in enthalpy (∆H_a_) and entropy (∆S_a_) associated with the binding events. All experiments were performed in triplicate.

## 3 Results

### 3.1 Protein structure modeling and *in silico* docking

#### 3.1.1 Structure prediction of the sdAbCSP1 – PfCSP-Cext complex

**Fig. 1:**
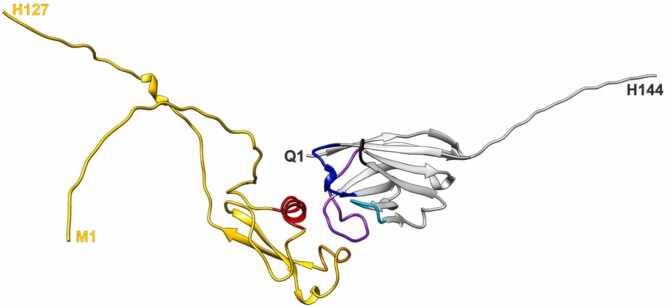
Three-dimensional structure representations of the sdAbCSP1 – PfCSP-Cext complex model from AlphaFold2. The backbones of the amino acid sequences are shown as ribbons (cartoon view). Gray-colored, sdAbCSP1; Gold-colored, PfCSP-Cext; Red-colored, alpha helix of PfCSP-Cext (aa40–52); Orange-colored, loop of PfCSP-Cext (aa83–90); Blue-colored, CDR1 (aa25–33); Cyan-colored, CDR2 (aa52–57); Purple-colored, CDR3 (aa100–118); Black-colored, HV4 (aa74–77) of sdAbCSP1. N-terminal and C-terminal amino acid residues are indicated (single letter code).

The structures of sdAbCSP1, PfCSP-Cext and the sdAbCSP1 – PfCSP-Cext complex were predicted from their amino acid sequences (Fig. S1) with relatively high confidence as judged from various local and global validation metrics (Fig. S2). For both sdAbCSP1 and PfCSP-Cext, local pLDDT values and overall zDOPE and pTM scores indicate that the structural models are reliable. Similarly, the sdAbCSP1 – PfCSP-Cext complex was modeled with high accuracy, as evidenced by the relevant scores (PAE, pDockQ, and ipTM), thereby revealing the paratope-epitope interactions (Table 1, Fig. 1, and Fig. S2). Interestingly, sdAbCSP1 appears to bind a hydrophobic pocket on PfCSP-Cext’s α-TSR domain surface, lined by the α-helix (aa40–52) and the opposing loop structure (aa83–90). The sdAbCSP1 paratope consists mainly of CDR3 residues (aa100–118).

**Table 1:** Amino acid residues encompassing the sdAbCSP1 – PfCSP-Cext complex interaction interface.

protein^a^	amino acid residues
sdAbCSP1	R27, S31, Y32, N57, Y59, L100, L101, Q102, F103, G104, R105, R108, A110, D111, Y112, D113, Y114
PfCSP-Cext	S29, K42, K45, E46, L48, N49, Q52, L55, K83, D84, E85, L86, D87, Y88, N90

a amino acid residues with the shortest atom distance ≤ 4 Å between PfCSP-Cext and sdAbCSP1.

Despite the sdAbCSP1 – PfCSP-Cext complex model from AlphaFold2 providing a clear and convincing prediction about the paratope-epitope interactions, alternative complex modeling experiments were performed to further validate the proposed complex model.

#### 3.1.2 Positive and negative controls of *in silico* docking experiments

To test alternative complex modeling approaches, the 3D coordinates of the individually modeled PfCSP-Cext and sdAbCSP1 proteins were submitted to HADDOCK 2.4 using different starting configurations. First, amino acid residues from PfCSP-Cext’s antigen aa46–58 (which encompasses most of the alpha helix (aa40–52)) were selected as the potential epitope, and CDR1, CDR2, and CDR3 of sdAbCSP1 were chosen to make contact to PfCSP-Cext’s partial surface, i.e*.,* to participate in the paratope surface. With this “head on” configuration of sdAbCSP1, a complex model was generated in which all three CDRs had made contact to PfCSP-Cext (complex #2; Fig. S3). Second, amino acid residues from PfCSP-Cext’s antigen aa46–58 (which encompasses most of the alpha helix (aa40–52)) were selected as the potential epitope, and only CDR3 of sdAbCSP1 was allowed to participate in complex formation as a potential paratope. With this “side on” configuration of sdAbCSP1, a complex model was generated (complex #3; Fig. S4). Third, to further lend confidence to the results, docking of sdAbCSP1 to PfCSP-Cext was performed starting with an incompatible orientation and CDR regions were placed such that they were facing away from PfCSP-Cext’s binding site. With this “backward” configuration of sdAbCSP1, a complex model was generated (complex #4; Fig. S5). Fourth, the docking experiment was performed without pre-selecting interacting surfaces, neither on PfCSP-Cext nor on sdAbCSP1. With this “random” configuration, a complex model was produced (complex #5; Fig. S6). Fifth, the amino acid sequence of PfCSP-C, which resembles amino acid residues aa39–100 from PfCSP-Cext and for which a crystal structure exists (PDB ID: 3VDJ, [56]) plus the amino acid sequence of truncated sdAbCSP1, which resembles amino acid residues aa1–125 from sdAbCSP1, i.e*.,* without non-structured C- and/or N-terminal extensions, were subjected to AlphaFold2 to model the sdAbCSP1 (tr) – PfCSP-C complex (complex #6; Fig. S2). Sixth and seventh, the 3D coordinates of all non-hydrogen atoms of the published complexes in which one partner was a single chain antibody (PDB files 1BZQ and 5USF) were submitted to HADDOCK 2.4 docking experiments to provide complex models (complex #7; Fig. S7 and complex #8; Fig. S8).

All modeled complexes, as well as the complex structures that are based on existing crystal structures, had been submitted to HADDOCK 2.4, through which a comparable scoring system was applicable for all complexes, independent of the origins of the complex model / structure. HADDOCK 2.4 sampled max. 200 structures into clusters, and in all cases, the cluster with the highest number of complex structures was chosen to represent the best group and the best score of a given complex (Table 2). The “two-step modeling” procedure for scoring the sdAbCSP1 – PfCSP-Cext complexes provided for complex #1, which was derived from AlphaFold2 modeling using default settings, a score of −107.3. This score was clearly different from the scores that were obtained when “manually” docking sdAbCSP1 and PfCSP-Cext with four different starting conditions (complexes #2, #3, #4 and #5). For these “negative controls” scores of about −60 were obtained. AlphaFold2 modeling with default settings of the truncated sdAbCSP1 – PfCSP-C complex (complex #6) resulted in a HADDOCK score of −103.5, which is well comparable to that of the sdAbCSP1 – PfCSP-Cext, complex and proved good reproducibility of the “two-step modeling” procedure. At last, the scores of the complexes originating from existing crystal structures, i.e*.,* complexes #7 and #8 (positive controls), provided scores of −92.6 and −142.6, respectively.

**Table 2:** Structure model characterizing metrics of the sdAbCSP1 – PfCSP-Cext complex and controls.

**No.**	**Complex**	**Starting configuration**	**HADDOCK score**^a^ **(a.u.)**	**Cluster**^a^ **size**	**RMSD**^b^ **(Å)**	**VdW energy**^c^ **(kcal / mol)**	**El. energy**^d^ **(kcal / mol)**	**Des. energy**^e^ **(kcal / mol)**	**RV energy**^f^ **(kcal / mol)**
1	sdAbCSP1 – PfCSP-Cext	default^g^	−107.3 ± 5.2	200	1.5 ± 1.2	−35.0 ± 5.3	−380.9 ± 23.6	3.7 ± 1.2	2.2 ± 0.7
2	sdAbCSP1 – PfCSP-Cext	head on^h^	−56.8 ± 3.1	26	23.4 ± 1.7	−37.5 ± 4.4	−157.6 ± 24.7	−2.3 ± 3.6	144.7 ± 61.9
3	sdAbCSP1 – PfCSP-Cext	side on^i^	−64.3 ± 2.7	28	18.9 ± 1.0	−33.0 ± 4.0	−181.5 ± 14.8	2.5 ± 0.9	25.2 ± 20.9
4	sdAbCSP1 – PfCSP-Cext	backwards^j^	−65.9 ± 9.8	28	29.6 ± 0.2	−30.6 ± 4.2	−258.0 ± 22.5	3.6 ± 1.2	126.7 ± 46.5
5	sdAbCSP1 – PfCSP-Cext	random^k^	−60.4 ± 7.4	4	30.3 ± 0.5	−23.5 ± 1.1	−209.5 ± 35.3	5.0 ± 2.2	0.0 ± 0.0
6	sdAbCSP1 (tr) – PfCSP-C	default^l^	−103.5 ± 2.8	188	0.7 ± 0.4	−29.7 ± 4.5	−384.7 ± 21.1	1.7 ± 1.8	14.7 ± 7.2
7	1BZQ	crystal^m^	−92.6 ± 1.3	180	2.2 ± 0.3	−54.1 ± 3.0	−106.0 ± 17.9	−17.9 ± 0.9	16.7 ± 11.7
8	5USF	crystal^n^	−142.6 ± 0.7	200	0.5 ± 0.4	−80.9 ± 3.3	−264.8 ± 20.4	−10.0 ± 1.4	12.0 ± 5.3

a Unitless numbers; a.u.: arbitrary units.

b Root mean square deviation (RMSD) from the overall lowest-energy structure, according to [57].

c Van der Waals energy.

d Electrostatic energy.

e Desolvation energy.

f Restraints violation energy.

g Starting configuration as from AlphaFold2 output applying full-length amino acid sequences of sdAbCSP1 and PfCSP Cext (cf. Fig. 1 and Fig. S2).

h CDR1, CDR2, and CDR3 from sdAbCSP1 head on brought into the vicinity of amino acid residues 46–58 from PfCSP Cext (cf. Fig. S3).

i CDR3 from sdAbCSP1 sideways brought into the vicinity of amino acid residues 46–58 from the PfCSP Cext antigen (cf. Fig. S4).

j CDR1, CDR2, and CDR3 from sdAbCSP1 facing away from the binding site (cf. Fig. S5)

k Without indicating preferred sites of sdAbCSP1 or PfCSP Cext (cf. Fig. S6).

l Starting configuration as from AlphaFold2 output applying truncated amino acid sequences, i.e., sdAbCSP1 (tr) and PfCSP C (cf. Fig. S2).

m Starting configurations as from 1BZQ or 5USF crystal structures (cf. Fig. S7 and Fig. S8).

Since scores of around −100 or below were obtained from AlphaFold2 modeling and from existing crystal structures, this score comparison suggests that AlphaFold2 produced a model for the sdAbCSP1 – PfCSP-Cext complex, which is considered excellent and provides a good suggestion of the interacting partial surfaces, i.e*.,* epitope and paratope of either complex constituent.

### 3.2 Mass spectrometric PfCSP-cext epitope mapping and sdAbCSP1 paratope mapping

Next, we sought to experimentally substantiate the *in-silico* epitope and paratope predictions through MS. Data were first collected for the intact and re-buffered sdAbCSP1 and PfCSP-Cext proteins. Mass spectra with strong multiply charged ion signals were recorded. The multiply protonated molecular ion signals of sdAbCSP1 were accompanied by a series of ion signals which, according to their *m/z* values, indicated the presence of a protein species with a loss of 18 Da, most likely a result of transforming the N-terminal glutamine residue into pyro-glutamine. The experimentally determined molecular mass of PfCSP-Cext matched well to the calculated molecular masses derived from the protein’s amino acid sequence (Fig. S9 and Tables S1, S2, and S3). In conclusion, the starting materials were quite pure and, hence, were considered well suitable for limited proteolysis experiments.

#### 3.2.1 PfCSP-Cext epitope mapping by limited proteolysis

**Fig. 2:**
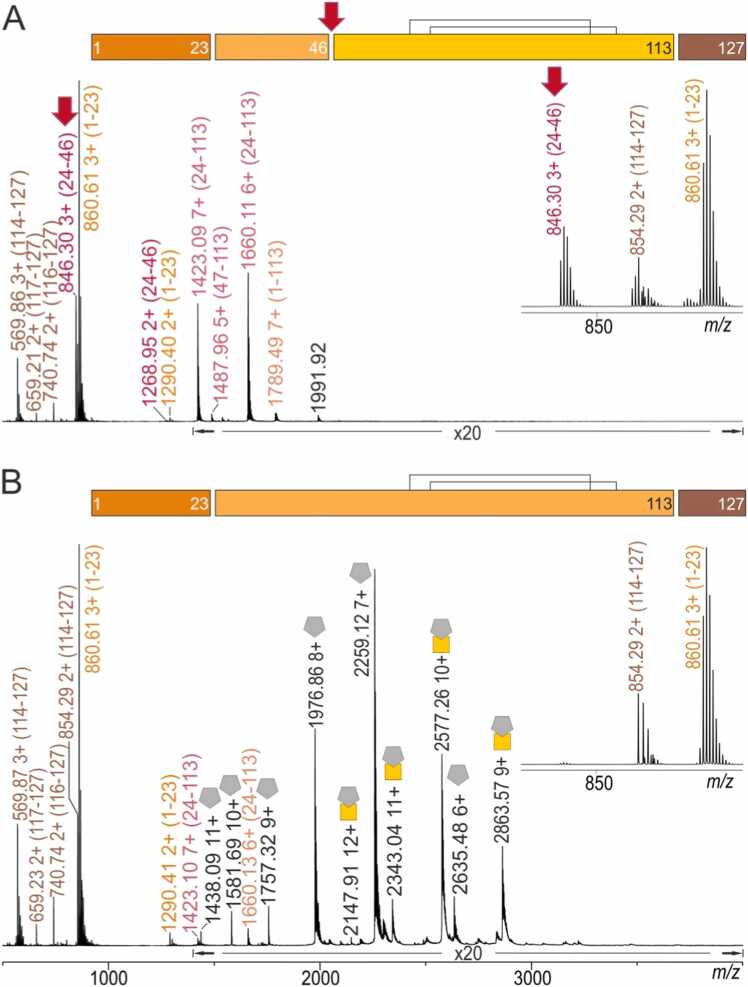
Peptide mapping by off-line nanoESI-MS^E^ analysis after 24 h limited GluC digestion of PfCSP-Cext and sdAbCSP1 – PfCSP-Cext complex. **A,** PfCSP-Cext alone. **B,** sdAbCSP1 - PfCSP-Cext complex with excess sdAbCSP1 (molar ratio of sdAbCSP1: PfCSP-Cext = 1.8: 1). Selected ion signals are labeled with *m/z* values, and charge states for ion signals are given. Gold colored square & gray colored pentagon, sdAbCSP1 – PfCSP-Cext complex; Gold colored square, PfCSP-Cext; Gray colored pentagon, sdAbCSP1. Bars on top of spectra represent full-length proteins and cleavage sites are shown as gaps. Numbers represent amino acid positions. Black lines indicate disulfide bridges. The red arrow marks the cleavage site, which was shielded by the complex formation, and the ion signal, which proves the difference (see inserts). Amplification factors are given. Solvent: 200 mM ammonium acetate, pH 6.7. For ion signal assignments, see Table S4.

Limited proteolysis using GluC after 24 h digestion time showed that the protease had cut only terminal sequence parts from PfCSP-Cext. The liberated peptides could be attributed either to the N-terminal partial amino acid sequence range aa1–46 or the C-terminal partial amino acid sequence range aa114–127, which contained the His-tag sequence (Fig. 2A and Table S4). Both terminal endings encompassed unstructured extensions that extruded from the compactly folded PfCSP-C domain (aa39–100 from PfCSP-Cext). Of note, the only cleavage site from within PfCSP-Cext’s compactly folded domain was observed to have taken place at amino acid residue E46, which is located in the center of PfCSP-Cext’s α-helix (aa40–52).

When the sdAbCSP1 – PfCSP-Cext complex-containing solution was submitted to 24 h limited proteolysis using GluC, the corresponding mass spectrum presented again peptide ion signals which belonged to PfCSP-Cext’s terminal peptides aa1–23 and aa114–127, respectively. Interestingly, no cleavage was observed at amino acid residue E46 (Fig. 2B and Table S4). However, the mass spectrum contained additional ion signals that were assigned to the intact sdAbCSP1 – PfCSP-Cext complex and to undigested sdAbCSP1, which was present in excess (molar ratio of sdAbCSP1: PfCSP-Cext was 1.8: 1).

Complex formation obviously had shielded the cleavage site E46 on PfCSP-Cext. This result indicated that the PfCSP-Cext epitope targeted by sdAbCSP1 included E46 and thus involved PfCSP-Cext’s α-helix (aa40–52).

To confirm this result, the limited digestion period was extended to 72 h GluC digestion. Peptide mixtures of either PfCSP-Cext or of sdAbCSP1 – PfCSP-Cext complex-containing solutions were compared, this time by nanoLC-ESI-MS^E^ analysis. The two LC chromatograms showed distinctive differences, which resulted from differentiating peptide compositions (Fig. S10 and Table S5). Cleavage of PfCSP-Cext at E46 was confirmed in the peptide mixture, which eluted in fraction 2 (Fig. S11) when PfCSP-Cext was digested. Fraction 2 was absent in the LC trace of the peptide mixture of the sdAbCSP1 – PfCSP-Cext complex-containing solution (Fig. S10). In both cases, the N-terminal peptides aa1–23 eluted in fractions 5 (Fig. S11). Interestingly, PfCSP-Cext showed, after 72 h, limited digestion some oxidation and deamidation (Fig. S12 and Table S5). Of note, yet another cleavage within the compactly folded PfCSP-C domain was observed after 72 h GluC limited digestion at E58, which resulted in peptide aa47–58 (Fig. S13). By contrast, no cleavage within the compactly folded PfCSP-C domain was observed after 72 h GluC limited digestion of the sdAbCSP1 – PfCSP-Cext complex-containing solution. Instead, the N- and C-terminally truncated PfCSP-Cext, which encompassed aa24–113, remained untouched and eluted in fraction 14 (Fig. S13 and Table S5), indicating again shielding of the α-TSR domain’s α-helix by complex formation. The fact that no peptides from sdAbCSP1 were observed indicated its resistance towards proteolysis by GluC.

To prove that the observed PfCSP-Cext cleavage differences were not results of GluC specificity, limited proteolysis was performed with trypsin using solutions with either PfCSP-Cext or with the sdAbCSP1 – PfCSP-Cext complex. The mass spectrum of the peptide mixture, which was obtained after trypsinolysis of PfCSP-Cext (Fig. S14A), showed that N-terminal cleavage sites K2, R19, K32, and K42 were susceptible to cleavage. No further cleavages were observed (Table S6). The mass spectrum also contained ion signals of multiply charged truncated PfCSP-Cext species aa20–127 and aa33–127, respectively. Likewise, N-terminal peptides with cleavages at PfCSP-Cext’s K2, R19, and K32 residues were found after tryptic digestion of the sdAbCSP1 – PfCSP-Cext complex together with ions of truncated PfCSP-Cext which encompassed aa30–127 (Fig. S14B). This result stands in agreement with the shielding of the α-helix (aa40–52) of PfCSP-Cext upon complex formation with sdAbCSP1, thereby again confirming that the PfCSP-Cext α-helix contained a part of the epitope.

It needs to be emphasized that the mass spectrum after trypsin digestion also contained ion signals of peptides that were assigned to sdAbCSP1 partial amino acid sequences, which was present in excess (molar ratio of sdAbCSP1: PfCSP-Cext was 1.8: 1) as well as of sdAbCSP1 – PfCSP-Cext complexes in which the sdAbCSP1 component was completely intact, i.e*.*, contained the full length amino acid sequence. However, despite being complexed, the PfCSP-Cext component of the complex was found to be N-terminally truncated and some complexes contained partial amino acid sequences aa20–127 or aa33–127 (Table S6). Yet another interesting truncation of sdAbCSP1 was observed in which only the partial amino acid sequence aa106–108 had been clipped off. Removal of the respective SSR tripeptide produced an sdAbCSP1 species in which the two protein pieces, aa1–105 and aa109–144, were kept together by non-covalent interactions leading to multiply protonated ion signals at *m/z* 2217.21 and *m/z* 1940.10. It should be emphasized that not a single sdAbCSP1 fragment had participated in complex formation.

#### 3.2.2 sdAbCSP1 paratope mapping by limited proteolysis

**Fig. 3:**
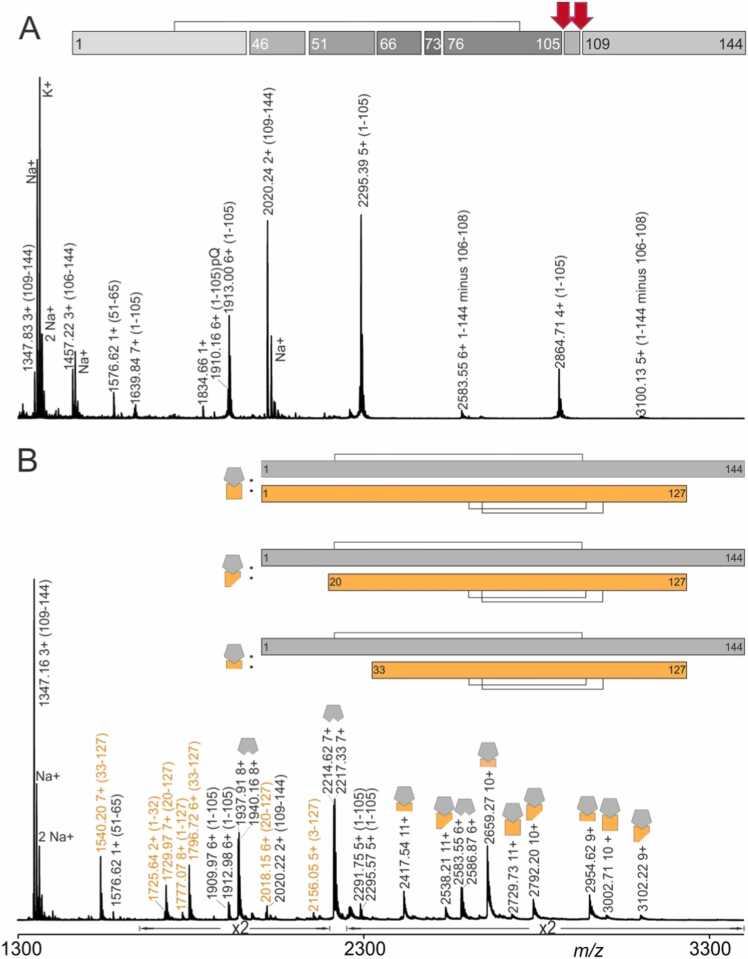
Peptide mapping by off-line nanoESI-MS^E^ analysis after 2 h limited tryptic digestion of sdAbCSP1 and of sdAbCSP1 – PfCSP-Cext complex. **A,** sdAbCSP1 alone. **B,** sdAbCSP1 – PfCSP-Cext complex with excess sdAbCSP1 (molar ratio of sdAbCSP1: PfCSP-Cext = 1.8: 1). Selected ion signals are labeled with *m/z* values, and charge states for ion signals are given. Gold-colored square & gray-colored pentagon, sdAbCSP1 – PfCSP-Cext complex; Gold-colored square, PfCSP-Cext; Gray-colored pentagon, sdAbCSP1. Bars on top of spectra represent full-length or truncated proteins and cleavage sites are shown as gaps. Numbers represent amino acid positions. Black lines indicate disulfide bridges. The red arrows mark the cleavage sites, which were shielded by complex formation. Amplification factors are given. Solvent: 200 mM ammonium acetate, pH 6.7. For ion signal assignments, see also Table S7.

Since sdAbCSP1 turned out to be susceptible to cleavage by trypsin (but not by GluC) and PfCSP-Cext turned out to be mostly resistant towards trypsin digestion (but not to digestion by GluC), we attempted a paratope mapping experiment with a 2 h limited trypsin digestion of either sdAbCSP1 or sdAbCSP1 – PfCSP-Cext complex. Peptide mapping after 2 h tryptic digestion of sdAbCSP1 and of sdAbCSP1 – PfCSP-Cext complex by offline nanoESI-MS^E^ analysis revealed the paratope to be located around residues R105 and R108 (Fig. 3, S15, and Table S7).

After 2 h limited tryptic digestion, sdAbCSP1 was cleaved predominantly at R105 and R108, thereby producing a peptide mixture that, in the mass spectrum, provided strong ion signals of three main protein fragments: aa1–105, aa106–108, and aa109–144. Ion signals with lesser intensities proved cleavages at sdAbCSP1’s R45, R50, R65, R72, and K76 residues (Fig. 3 and S15). All these ion signals were also present in the mass spectrum of the peptide mixture, which was obtained after 2 h limited trypsinolysis of the sdAbCSP1 – PfCSP-Cext complex (Fig. 3, S15, and Table S7). Again, the dominating ion signals were assigned to the truncation of sdAbCSP1 in which the partial amino acid sequence aa105–108 had been clipped off, and the so truncated sdAbCSP1 contained the two protein pieces aa1–105 and aa109–144, which were kept together by non-covalent interactions. The mass spectrum also contained strong ion signals of the sdAbCSP1 – PfCSP-Cext complex, in which only the PfCSP-Cext component was either intact or N-terminally truncated. Again, the sdAbCSP1 component in the complex remained intact. Obviously, complexation shielded the partial amino acid sequence aa105–108 and other cleavage sites from trypsinolysis. Because of the dominating ion signals, cleavage at R105 and R108 is considered important for sdAbCSP1’s losing its ability to bind to PfCSP-Cext. The complete absence of these cleavages in the sdAbCSP1 – PfCSP-Cext complex promotes the partial amino acid sequence SSR, aa106–108, from being part of sdAbCSP1’s CDR3 to the region which must contain the paratope.

### 3.3 sdAbCSP1 – PfCSP-cext complex binding strength analysis

#### 3.3.1 *In-silico* analysis of sdAbCSP1 – PfCSP-Cext complex stability variations

**Fig. 4:**
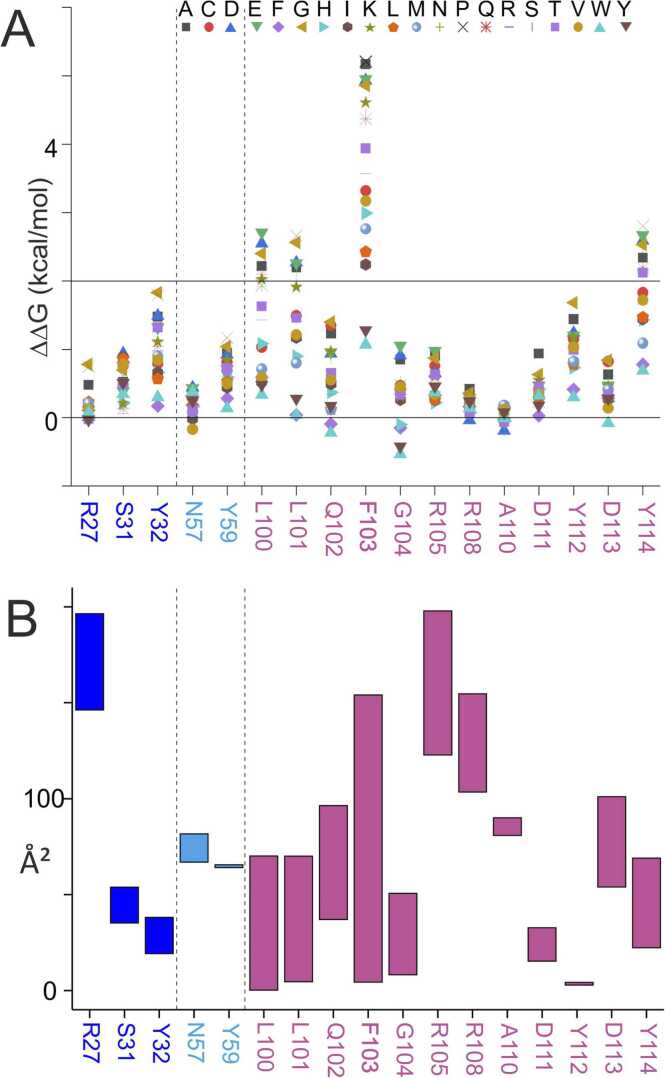
Changes in free energies and surface accessibility changes accompanied with single amino acid exchanges of paratope-residing amino acid residues. **A,** Relative free energy changes (ΔΔG values in kcal / mol) caused by single amino acid exchanges of sdAbCSP1 at each position which makes contact with PfCSP-Cext. The horizontal line at 2 kcal/mol marks the upper limit of the range of energy differences whose values are assumed to be negligible. **B,** Bar diagram of SASA differences. Top of the bar: SASA of the amino acid residue in unbound sdAbCSP1. Bottom of the bar: SASA of the amino acid residue in sdAbCSP1 – PfCSP-Cext complex. For individual values, see Tables S8 and S10.

Since limited proteolysis and MS analysis of peptide mixtures confirmed that (i) PfCSP-Cext’s α-helix contained at least a part of the AlphaFold2 predicted epitope and that (ii) sdAbCSP1’s CDR3 contained the paratope around amino acid residues R105 and R108, both predicted and experimentally confirmed epitope and paratope regions were interrogated to determine the key amino acid residues residing therein. To do this, binding energy changes were *in silico* calculated by introducing point mutations at each position of the interacting amino acid residues (cf. Table 1), and the resulting energy changes were calculated as ΔΔG increments, which were added to the arbitrarily set value (ΔG=0) of the default amino acid residue from the amino acid sequence at its respective position within the paratope (Fig. 4A, Table S8) or within the epitope (Fig. S16A, Table S9). An amino acid exchange that weakens the complex results in a positive ΔΔG value, and one that strengthens the complex in a negative ΔΔG value.

By free energy difference calculations, the most prominent amino acid residue in sdAbCSP1’s CDR3 is F103. The F103P (ΔΔG: 5.21 kcal/mol) and F103A (ΔΔG: 5.17 kcal/mol) exchanges caused the strongest energy penalties, i.e*.,* weakened the complex most. Most of the other amino acid exchanges at the F103-neighboring paratope positions caused energy penalties of around and below 2 kcal/mol (Fig. 4A and Table S8). Moreover, surface accessibilities of amino acid residues of either the paratope or the epitope were expected to change decisively upon complex formation. The largest change in surface accessibility within sdAbCSP1’s paratope was found at F103. The accessible surface of F103 in sdAbCSP1 was determined to be 154.65 Å^2^, dropping to 4.36 Å^2^ in the sdAbCSP1 – PfCSP-Cext complex (Fig. 4B and Table S10).

Likewise, energy penalties and surface accessibility changes, which are accompanied with single amino acid exchanges of PfCSP-Cext’s epitope-residing amino acid residues, pointed to two leucine residues, L48 and L86, as being of interest (Fig. S16 and Table S9 and Table S11). The L48P (ΔΔG: 2.72 kcal/mol) and the L86P (ΔΔG: 2.62 kcal/mol) exchanges caused substantial complex binding strength weakening, whereas all other exchanges resulted in energy penalties, which stayed mostly at or below 2 kcal/mol (Fig. S16 and Table S9). Interestingly, the largest changes in surface accessibilities on the PfCSP-Cext epitope were found at K45 and D84. K45 is part of PfCSP-Cext’s α-helix, and D84 lies within the opposing loop, which was also predicted to be involved in binding to sdAbCSP1. Whereas the accessible surfaces of K45 and D84 in PfCSP-Cext were determined to be 123.28 Å^2^ and 135.07 Å^2^, respectively, they dropped to 9.20 Å^2^ and 24.38 Å^2^, respectively, in the sdAbCSP1 – PfCSP-Cext complex (Fig. S16B and Table S11). The fact that such “hot spots” were found within the predicted paratope as well as within the predicted epitope, lends further reliability to the *in-silico*-derived complex model structure.

#### 3.3.2 Mass spectrometric ITEM-TWO analysis of complex dissociation in the gas phase

To quantify the binding strength of the sdAbCSP1 – PfCSP-Cext complex, we performed ITEM-TWO analyses. The sdAbCSP1 – PfCSP-Cext complex was generated by mixing two solvents, one which contained sdAbCSP1, dissolved in ammonium acetate, and the other in which PfCSP-Cext was dissolved also in ammonium acetate (molar ratio of sdAbCSP1: PfCSP-Cext was 1: 3.4). The experimentally determined masses of all constituents of the protein mixture matched well with the respective calculated masses (Table S12).

**Fig. 5:**
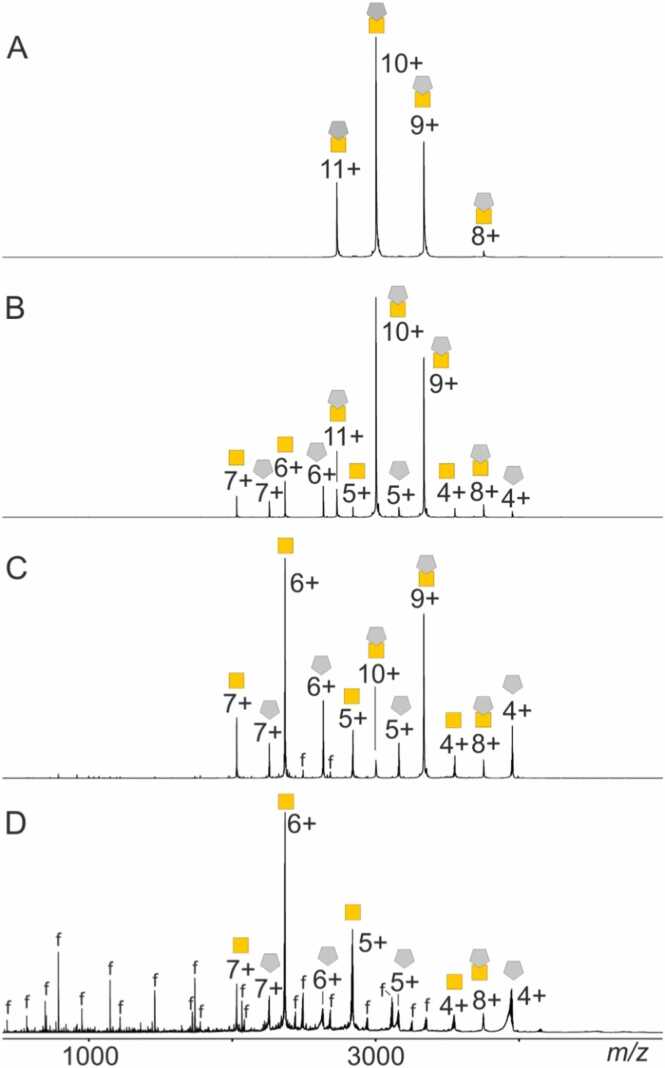
Offline nanoESI mass spectra of sdAbCSP1 – PfCSP-Cext complex dissociation at different collision cell voltage difference settings. **A,** ΔCV 10 V. **B,** ΔCV 40 V. **C,** ΔCV 70 V. **D,** ΔCV 100 V. Molar ratio of sdAbCSP1: PfCSP-Cext = 1: 3.4. Ion signals are labeled with charge states. Gold-colored square & gray-colored pentagon, sdAbCSP1 – PfCSP-Cext complex; Gold-colored square, PfCSP-Cext; Gray-colored pentagon, sdAbCSP1; f: fragments. The quadrupole was set to block transmission of ions with *m/z* < 2400. Solvent: 200 mM ammonium acetate, pH 6.7. For ion signal intensities, see Tables S13, S14, and S15.

By electrospray ionization of the mixture, the sdAbCSP1 – PfaCSP-Cext complex was transferred into the gas phase intact, together with residual sdAbCSP1 and PfaCSP-Cext as was deduced from the mass spectrum in which multiply charged ion signals of the complex were found together with multiply protonated ion signals of the two individual proteins (Fig. S17). A typical ITEM-TWO experiment starts with isolating the multiply charged ions of the sdAbCSP1 – PfCSP-Cext complex whereas all other ions are deflected (Fig. 5A), and continues with step-wise increasing the collision cell voltage (ΔCV) through which the ionized complex starts to dissociate (Figs. 5B and 5C). At very high ΔCV values, the complex components also began to fragment (Fig. 5D).

At each ΔCV setting, all ion intensities of all protein species, sdAbCSP1, PfCSP-Cext, and sdAbCSP1 – PfCSP-Cext complex, were determined. For each protein species, the mean charge states were calculated together with the intensities of the means of ion signals (Tables S13, S14, and S15). Interestingly, the complex-released sdAbCSP1 appeared in two conformations, which were distinguishable by their charge structures. For the extended conformation (sdAbCSP1), the 6 + ion signal was the strongest and for the compact conformation (sdAbCSP1’), the 4 + ion signal was of highest intensity (Fig. 5C). Then, all mean intensities (Table S16) were summed up, and the individual protein’s mean intensities were normalized. ITEM-TWO analyses were performed in triplicate, and the courses of normalized and averaged mean ion signal intensities of the sdAbCSP1 – PfCSP-Cext complex (starting material, educt) were then plotted as a function of collision cell voltage difference (Fig. S18). The accurately fitted line (R^2^ = 0.991) is a curve that follows Boltzmann characteristics (Table S17).

From the steep part of the Boltzmann curve, i.e*.,* the tangent line, one extracts the region within which the collision cell voltage difference change yields a proportional fraction of complex dissociation. After converting the ΔCV axis into a collision temperature ($T_{\mathrm{coll}}$), one draws an Arrhenius plot (Fig. S19) to calculate the apparent rate constant $k_{\mathrm{D m}0g}^{\#}$ (Table 3). Applying the Eyring-Polanyi equation yields in the apparent quasi equilibrium constant $K_{\mathrm{D m}0g}^{\#}$. From the van’t Hoff equation and plotting experimental ${\Delta G}_{\mathrm{mg}}^{\#}$ versus $T_{\mathrm{coll}}$ one determines ${\Delta S}_{\mathrm{mg}}^{\#}$ as the negative slope of the line. At last, with the help of the Gibbs-Helmholtz equation, one obtains ${\Delta H}_{m0g}^{\#}$, and ${T_{\mathrm{amb}}\Delta S}_{m0g}^{\#}$ (Fig. S20, Table 3).

**Table 3:** Apparent kinetic and quasi thermodynamic values for sdAbCSP1 – PfCSP-Cext complex dissociation a.

complex	$k_{\mathrm{D m}0g}^{\#}$^b^^,^^c^ [Ø]	$K_{\mathrm{D m}0g}^{\#}$^b^ [1/s]	${\Delta G}_{m0g}^{\#}$^b^ [kJ/mol]	${\Delta H}_{m0g}^{\#}$^b^ [kJ/mol]	${T_{\mathrm{amb}}\Delta S}_{m0g}^{\#}$^b^^,^^d^ [kJ/mol]
sdAbCSP1 – PfCSP-Cext	1.0 × 10^10^	3.7 × 10 ^−12^	15.7	50.2	34.5

a apparent (#), mean charge state (m), no external energy contribution (0), gas phase (g), dissociation (D).

b accuracy approx. 10 %

c unitless number

d $T_{\mathrm{amb}}$: 298 K

Since dissociation of the sdAbCSP1 – PfCSP-Cext complex in the gas phase is endergonic, i.e*.,* not spontaneous (${\Delta G}_{m0g}^{\#}$ > 0), and endotherm (${\Delta H}_{m0g}^{\#}$ > 0), one concludes that the opposite reaction, i.e*.,* complex formation, is exergonic, i.e*.,* spontaneous (ΔG < 0), and exotherm (ΔH < 0). Dissociation of the sdAbCSP1 – PfCSP-Cext complex in the gas phase increases entropy (${T_{\mathrm{amb}}\Delta S}_{m0g}^{\#}$ > 0), which is the driving force of the reaction. On the contrary, complex formation is expected to either cost entropy (TΔS < 0) or increase entropy (${T_{\mathrm{amb}}\Delta S}_{m0g}^{\#}$ < 0), but should be enthalpy driven.

#### 3.3.3 *In-solution* ITC complex binding strength analysis

**Fig. 6:**
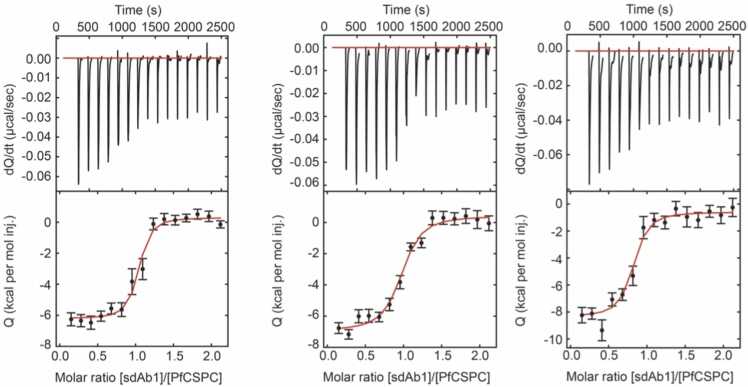
ITC - ITC measurements at 25 °C for the binding of sdAbCSP1 to PfCSPC. Each replicate measurement is shown. The top panels represent the thermograms in which the black lines depict the raw data. The bottom panels show the isotherms. The black dots display the experimental data points, and the red traces show the fit.

The *in-solution* thermodynamic properties of sdAbCSP1 – PfCSP-C complex formation (Fig. 6) were determined using ITC. With a K_D_ of 5 × 10^-10^ M, the sdAbCSP1 – PfCSP-C complex is considered very strong (Table S18). As expected, complex formation is spontaneous (exergonic; ΔG_a_: - 48.8 kJ/mol) and enthalpy driven (ΔH_a_: - 34.9 kJ/mol). Entropy increases during complex formation (TΔS_a_: 13.8 kJ/mol) but is accompanied by a high exothermic term.

## 4 Discussion

Combining peptide level MS results with 3D atom coordinates of experimentally determined protein structures or available 3D structure models has been applied for many years to obtain precise information on protein structural details, such as disulfide bond linkages [58], reactive surface sites [59], or protein ligand interaction sites [60], [61]. Likewise, early developments in chemical protein modification [59], [62], limited proteolysis [22], [23], [24], and cross-linking [63], [64] in combination with MS have laid the grounds for investigating protein complexes by MS with respect to determining the stoichiometry of complex constituents [65], binding surfaces [66], [67], and binding strengths [52], [68]. The “constraint-based” modeling approach has since been incorporated into “structural proteomics” [69]. However, the opposite, i.e*.,* first predicting protein structures by *in silico* molecular modeling and then adding experimental evidence by MS, has been reported [70], also with the aim to predict epitopes and paratopes of immune complexes [44]. In line with this “modeling first” approach, we have used AlphaFold2 to predict the epitope and the paratope of the sdAbCSP1 – PfCSP-Cext complex and found that the predicted complex structure stands in line with experimental evidence, which in our case was collected by limited proteolysis and analysis of the produced peptides by mass spectrometry.

To elucidate epitopes and paratopes, respectively, limited proteolysis can be performed with any protease, and the conditions which need to be set also require the choice of an enzyme to substrate ratio, pH of the buffer, and other solvent conditions, such as salt strength, the addition of chaotropes, the addition of organic co-solvents, etc. to come to favorable conditions. When changing any of these variables, one must also consider that protein complex formation, as a dynamic process, might be affected. Speed of digestion with respect to complex dynamics is crucial. When digestion is faster than complex formation, any information about which sites are protected because of complexation may be lost, because once the complex partners had been liberated, they would be rapidly digested further into smaller fragments. However, when digestion is slower than complex formation, one can accumulate intermediate fragments over time, which, though no longer capable of forming complexes, retain fairly intact intermediate structures. From such intermediate proteolytic fragments one can deduce information about those molecular surfaces which had been shielded by complexation. To reach these ideal states, the limited proteolysis reaction has to be slowed down enough to allow enrichment of the intermediates of interest while at the same time complex formation ought not to be significantly affected. Both goals were achieved in this study by lowering the pH of the solution to 6.7, which is below trypsin’s digestion optimum. Noteworthy, the analysis of limited proteolysis products is greatly facilitated when one of the two complex partners remains resistant to digestion while the other is susceptible. This happened to be the case here where PfCSP-Cext turned out to be resistant to tryptic cleavage, whereas sdAbCSP1 was not digested by GluC. Such rather selective digestion behavior is not expected to be obtained with less specific proteases.

There are alternatives to the limited proteolysis approach. HDX-MS has the advantage of providing information about complex shielding without requesting resistance to digestion of one of the complex partners. However, analysis of HDX is a highly sophisticated procedure and requires special and costly equipment. Of note, suitable (pepsin) cleavage sites are required when performing HDX experiments to produce well analyzable peptides. In addition, high sequence coverage is of importance to not overlook shielded partial surfaces [71]. To set point mutations by genetic cloning is generally possible for studying the roles of specific amino acid exchanges and is a means of prediction verification as well. Yet, this procedure is typically time consuming and requires additional lab resources. A crucial limitation of the mutation-based approach is that one first needs to make sure that the mutation under consideration doesn't affect the structural stability of the protein or causes changes in the protein’s overall folding [72]. It should be noted that although helpful for determining structural details, limited proteolysis is not considered a suitable method for studying dynamics of binding in most cases of protein complex formation.

Protein structures and functions can be affected by post-translational modifications and glycosylation of CSP has been brought into context with potential masking of functional CSP epitopes [73]. The PfCSP-Cext protein which was studied here is of recombinant origin, produced by overexpression in *E. coli*. Although one cannot completely rule out glycosylation when using bacterial expression systems, this modification should have been picked up by our mass spectrometric molecular mass analyses since the associated mass increment addition is well in the range of the instrument’s resolution. Since by molecular mass determinations or by peptide mapping we have only found loss of water to form pyroglutamate, protein glycosylation and possible interference in binding strength was dismissed.

Using HADDOCK 2.4 for scoring the accuracy of the sdAbCSP1 – PfCSP-Cext complex model is a practical “two-stage approach” since the source of the 3D coordinates, i.e*.,* either an existing 3D structure or a structure model, was accepted without discrimination by the software for performing the scoring calculations. For comparison, other protein complex scoring algorithms, such as DockQ [74], require a benchmark structure, ideally an experimentally determined 3D protein structure. Moreover, current scoring algorithms typically consider the preciseness of all the complex participating constituents' atom coordinates [75], [76]. Yet, when it comes to predicting the epitope and/or the paratope of a protein complex, one is primarily interested in learning the positioning of the contact-making amino acid residues. In contrast, the positioning of atoms from other domains of the interacting proteins or the overall protein – protein orientation is less important. Thus, an “overall” scoring algorithm may be discouraging to accept an *in-silico* prediction despite the fact that the algorithm possibly had predicted the positions of the most intriguing “key residues” in either the epitope or the paratope, or in both, with sufficient or even high accuracy. It remains to be seen whether or not next generation protein complex prediction and scoring algorithms, such as the ones of the recently launched AlphaFold3 program [77] shall be distinguishing more between the two options. Here, we applied an approach using AlphaFold2 to predict the sdAbCSP1 – PfCSP-Cext complex interface structure in combination with limited proteolysis and mass spectrometry.

**Fig. 7:**
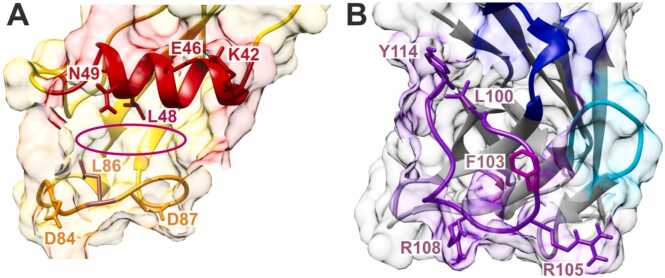
Structure model of the sdAbCSP1 – PfCSP-Cext complex. **A,** PfCSP-Cext’s epitope is generated by an α-helix (red) and a loop (orange) which together form a cavity (red oval) for capturing the protruding F103 amino acid residue from the paratope. **B,** sdAbCSP1’s paratope is formed by the CDR3 segment (purple). Backbone atoms are shown as ribbons (cartoon view) and side chains of key amino acid residues are depicted as sticks. Labels use amino acid single letter code and indicate the position in the amino acid sequence. Molecular surfaces are depicted as transparent van der Waal’s spheres. For color codes refer to Fig. 1.

Taking all the *in-silico* data into account which were obtained from the sdAbCSP1 – PfCSP-Cext complex structure model, i.e*.,* free energy difference changes (ΔΔG) and SASA changes, it is tempting to speculate that the predicted paratope provides five “key residues”: L100, F103, R105, R108, and Y114 (Fig. 7), where the central F103 amino acid residue of the paratope is flanked by Y114 and L100 (upper left corner), by R108 (lower left corner), and by R105 (lower right corner). The “corner” amino acid residues are involved in intermolecular hydrogen bonds connecting sdAbCSP1’s paratope to PfCSP-Cext’s epitope, whereas the central F103 residue makes hydrophobic interactions to amino acid residues from a cavity within the epitope.

The matching surface geometry on the epitope includes seven “key residues”: K42, E46, L48, N49, D84, L86, and D87. The first four amino acid residues are placed on PfCSP-Cext’s α-helix and the latter three on the adjacent loop (Fig. 7). The cavity, which is formed by these two secondary structure features places L48 and L86 into ideal positions for accepting F103 from the paratope in between them, thereby generating a sandwich-like structure. The flanking hydrogen bonds then connect (i) K42 and E46 from PfCSP-Cext’s α-helix (epitope) with Y114 from sdAbCSP1’s CDR3 (paratope), (ii) D87 from PfCSP-Cext’s loop (epitope) with R108 from sdAbCSP1’s CDR3 (paratope), (iii) D84 from PfCSP-Cext’s loop (epitope) with R105 from sdAbCSP1’s CDR3 (paratope), and (iv) N49 from PfCSP-Cext’s α-helix (epitope) with L100 from sdAbCSP1’s CDR3 (paratope).

The here predicted epitope on PfCSP-Cext’s surface, which is recognized by sdAbCSP1’s CDR3, resembles the interaction of Fab1710 with PfCSP-C [78]. A prominent hydrophobic interaction was found in the Fab1710 - PfCSP-C complex, flanked by hydrogen bonds between epitope and paratope. The amino acid residue Y360 from Fab1710’s HCDR3 was placed in the center of the so called α-epitope where it interacted with L320 from PfCSP-C’s α-helix. The amino acid residue L320 from PfCSP-C’s α-epitope refers to L48 in PfCSP-Cext’s α-helix. Other key residues of the PfCSP-C α-epitope are K314 (equals K42 on PfCSP-C’s α-helix), which is involved in hydrogen bonding to the paratope (LCDR1). K317 (equals K45) is involved in hydrogen bonding to HCDR3. E318 (equals E46) is involved in hydrogen bonding to LCDR2. And N321 (equals N49) is involved in hydrogen bonding to HCDR3 of Fab1710’s paratope.

The excellent performance of *in-silico* prediction methods was proven for sdAb structures [79]. And the prediction of protein complex structures by *in-silico* methods has in the meantime become so strong that the design of protein-binding proteins has become possible from the target structure alone [80]. Despite this truly remarkable success, predicted protein – protein complex structures could be refined by adding experimental data, such as “surface fingerprints” [81]. Moreover, adding experimental data to *in-silico* predicted protein structures has been found helpful for analyzing protein dynamics, i.e*.,* helping to distinguish multiple conformations of proteins [82].

With the here described results we were able to define (i) the interacting sites and (ii) the binding strength of the sdAbCSP1 – PfCSP-Cext complex, the two most important molecular features of any protein complex which might be considered as the “two faces of the same coin”. While the first is oriented on structure elucidation of a protein - protein complex, the second aims at a functional property of the same complex. As both features are strongly interwoven, they have been studied together. Determining implications of sdAbCSP1 – PfCSP-Cext complex formation of also other sdAbCSP’s is the main subject of ongoing and future studies which in particular aim at obtaining comprehensive explanations on specific molecular interactions, and more generally on biological activity of sdAbs in relevant cell or animal models.

## 5 Conclusions

This is the first report in which ITEM MS has been applied on a protein – protein complex system with camelid sdAbs. As shown with the sdAbCSP1 – PfCSP-Cext complex, MS-complemented molecular modeling allows generating hypotheses about the key residues placed at the right positions within the epitope and paratope surfaces and their types of force interactions. Our here described procedure provides a rapid and resource-efficient roadmap for analyzing dynamic protein – protein interactions, such as complex formation, by studying protein complexes and their free protein constituents on the sub-epitope and/or sub-paratope level.

## Funding

R. Geens is a doctoral fellow supported by a DOCPRO4-NIEUWZAP (code 40043) grant awarded to Y.G.-J. Sterckx by the University of Antwerp ‘Bijzonder Onderzoeksfonds (10.13039/501100007229BOF)’. L. De Vocht is a doctoral fellow supported by the FWO-Vlaanderen (11P4B24N). The WATERS Synapt G2S mass spectrometer has been bought through an EU grant [EFRE-UHROM 9] made available to Michael O. Glocker.

## CRediT authorship contribution statement

**Michael Glocker:** Writing – original draft, Supervision, Resources, Funding acquisition, Conceptualization. **Yann G.-J. Sterckx:** Writing – original draft, Supervision, Project administration, Methodology, Funding acquisition, Conceptualization. **Christophe Debuy:** Formal analysis. **Pieter Van Wielendaele:** Software, Investigation. **Manuela Ruß:** Validation, Formal analysis. **Kwabena F.M. Opuni:** Writing – original draft, Software, Methodology, Investigation, Formal analysis. **Line De Vocht:** Validation, Formal analysis. **Rob Geens:** Validation, Formal analysis.

## Declaration of Competing Interest

The authors declare no conflicts of interest.

## Data Availability

The mass spectrometry data have been deposited to the ProteomeXchange Consortium via the PRIDE [48] partner repository with the dataset identifier PX D051302.
