# Supplementary material for: Mass spectrometry-complemented molecular modeling predicts the interaction interface for a camelid single-domain antibody targeting the Plasmodium falciparum circumsporozoite protein’s C-terminal domain
Source: Comput Struct Biotechnol J. 2024 Aug 28;23:3300–14. doi: 10.1016/j.csbj.2024.08.023 (PMC11409006; doi:10.1016/j.csbj.2024.08.023)
Supplement: Supplementary file 1 — Supplementary material [file mmc1.docx]

Supplement

Mass spectrometry-complemented molecular modeling predicts the interaction interface for a camelid single-domain antibody targeting the *Plasmodium falciparum* circumsporozoite protein’s C-terminal domain

Kwabena F.M. Opuni 1,*), Manuela Ruß 2,*), Rob Geens 3), Line De Vocht 3), Pieter Van Wielendaele 3), Christophe Debuy 3), Yann G.J. Sterckx 3), and Michael O. Glocker 2,#)

1) Department of Pharmaceutical Chemistry, School of Pharmacy, College of Health Science,

University of Ghana, P.O. Box LG43, Legon, Ghana.

2) Proteome Center Rostock, University Medicine Rostock and University of Rostock,

Schillingallee 69, 18057 Rostock, Germany.

3) Laboratory of Medical Biochemistry, Faculty of Pharmaceutical, Biomedical, and Veterinary Sciences, University of Antwerp, Universiteitsplein 1, 2610 Wilrijk, Antwerp, Belgium.

* these authors contributed equally

# to whom correspondence may be addressed

Prof. Dr. Michael O. Glocker

Proteome Center Rostock

University Rostock Medical Center and Natural Science Faculty

University of Rostock

Schillingallee 69

18057 Rostock

Germany

Phone: +49 - 381 - 494 4930

FAX: +49 - 381 - 494 4932

e-mail: michael.glocker@med.uni-rostock.de

URL: https://pzr.med.uni-rostock.de

# Supplemental Figures


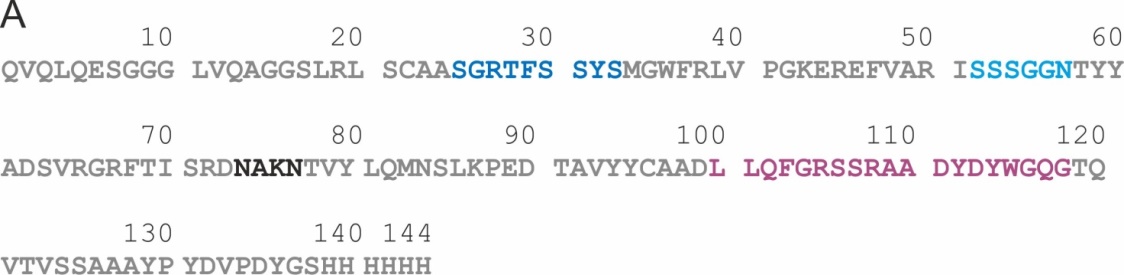


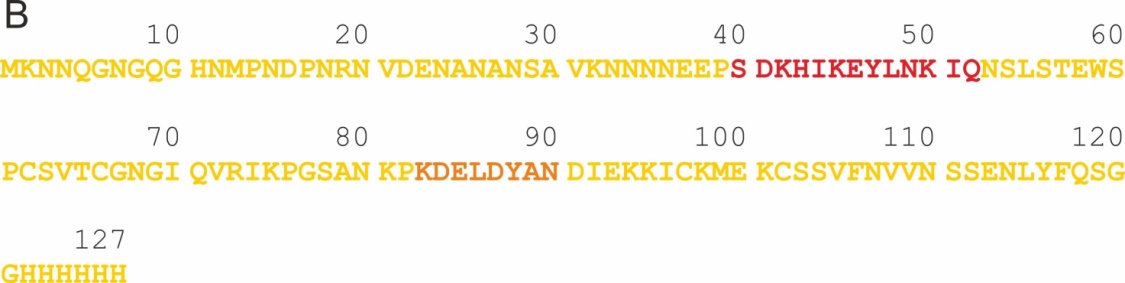


**Figure S1.** Amino acid sequences of sdAbCSP1 and PfCSP-Cext. **A,** Amino acid sequence of sdAbCSP1. CDR- and HV4-encompassing partial amino acid sequences are printed in blue (CDR1, aa25-33), cyan (CDR2, aa52-57), purple (CDR3, aa100-118), and black (HV4, aa74-77), respectively. **B,** Amino acid sequence of PfCSP-Cext. Helix- and loop-encompassing partial amino acid sequences are printed in red (aa40-52), and orange (aa83-90), respectively. Amino acid residues are shown in single letter code.

**Figure S2.** Overview of the AlphaFold2 prediction models for the sdAbCSP1 – PfCSP-Cext and sdAbCSP1 – PfCSP-C (tr) complexes and associated quality metrics. The models are displayed as cartoon representations and are colored according to the predicted local distance difference test (pLDDT) score, which reflects (local) model quality as indicated by the legend at the bottom. For all structures, the predicted aligned error (PAE), the normalized discrete optimized protein energy (zDOPE), the pDockQ and AlphaFold-Multimer model confidence (0.8*ipTM + 0.2*pTM) are also shown. The PAE provides a distance error for every residue pair, and is calculated for each residue x (scored residue) when the predicted and true structures are aligned on residue y (aligned residue). A zDOPE < -1 indicates that the distribution of atom pair distances in the model resembles that found in a large sample of known protein structures and that at least 80% of the model’s Cα atoms are within 3.5 Å of their correct positions. The pTM (score between 0 and 1) provides a measure of similarity between two protein structures (in this case, the predicted and unknown true structure) over all residues and thus reports on the accuracy of prediction within a single chain. The interface pTM (ipTM, score between 0 and 1) provides a measure of similarity between two protein structures (in this case, the predicted and unknown true structure) over only interfacing residues and thus reports on the accuracy of prediction for a complex. The pDockQ score (between 0 and 1) is another confidence metric for protein complexes that considers the number of interfacing residues and their pLDDT scores. Determination of the pDockQ score can be associated to a positive predictive value (PPV), which provides an estimate for the probability that the solution is a true positive. For the structure characterizing metrics, see structures #1 and #6 from Table 2.


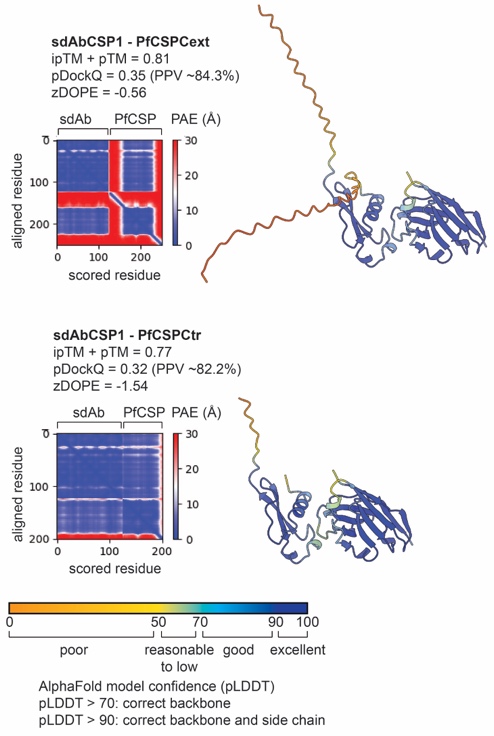


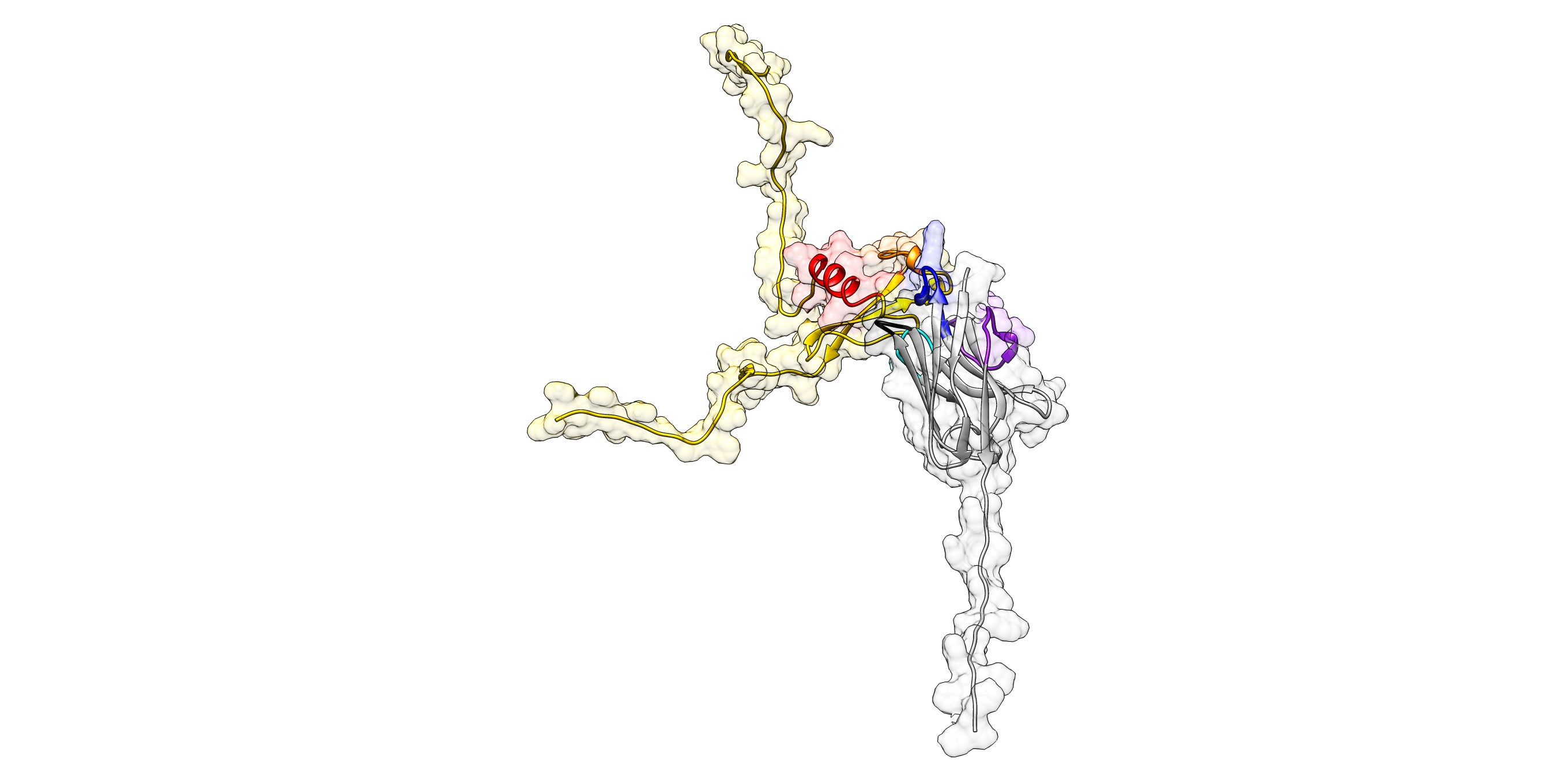


**Figure S3.** Three-dimensional structure representations of the sdAbCSP1 – PfCSP-Cext complex model from HADDOCK docking. Complex obtained using “head on” configuration. The backbone of the amino acid sequence is shown as a transparent spacefill and ribbon model (cartoon view). Gray-colored, sdAbCSP1; Gold-colored, PfCSP-Cext; Red-colored, helix of PfCSP-Cext (aa40-52); Orange-colored, loop of PfCSP-Cext (aa83-90); Blue-colored, CDR1 (aa25-33); Cyan-colored, CDR2 (aa52-57); Purple-colored, CDR3 (aa100-118) of sdAbCSP1; Black-colored, HV4 (aa74-77) of sdAbCSP1. For the structure characterizing metrics, see structure #2 from Table 2.


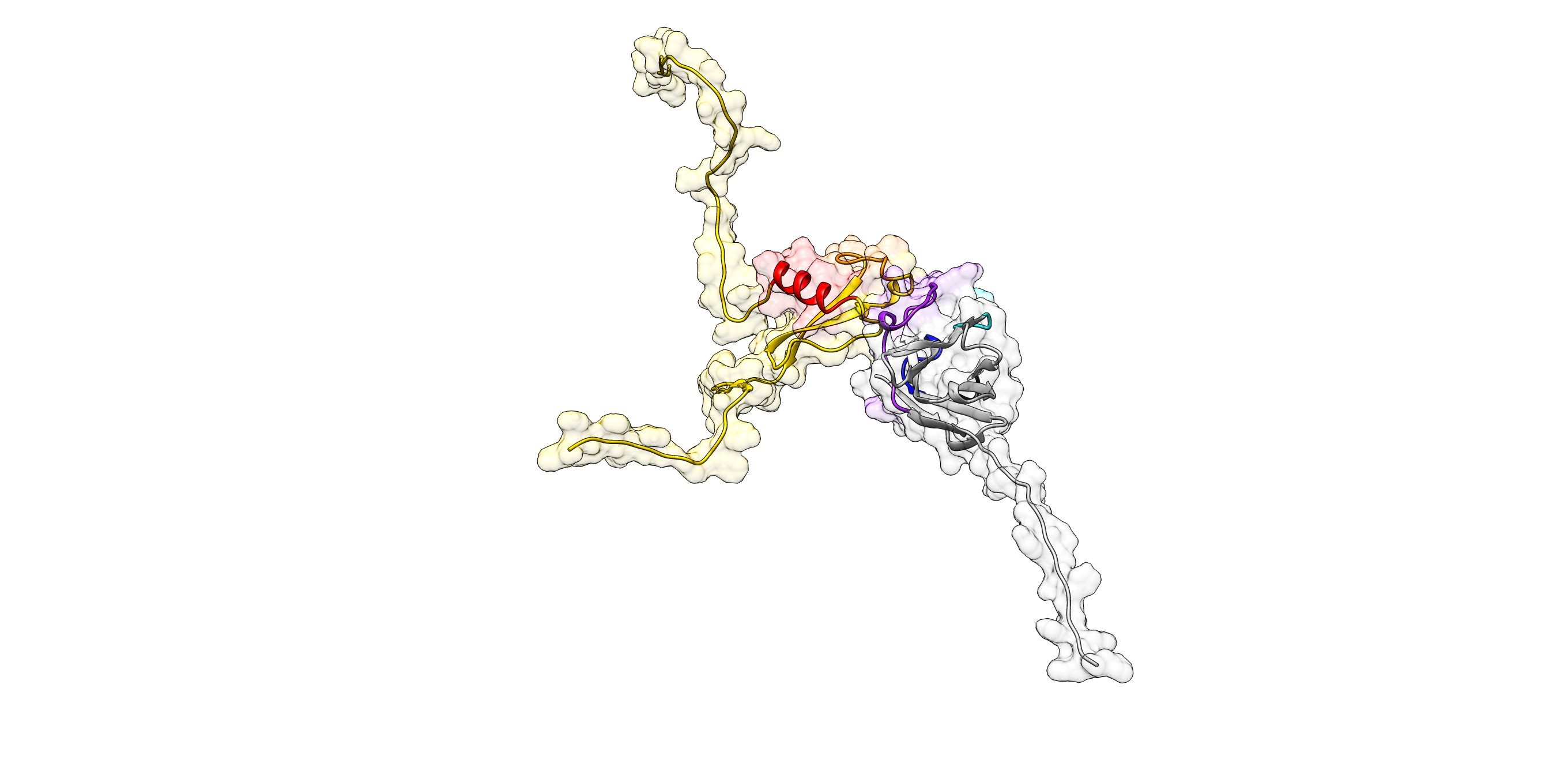


**Figure S4.** Three-dimensional structure representations of the sdAbCSP1 – PfCSP-Cext complex model from HADDOCK docking. Model obtained using “side on” configuration. The backbone of the amino acid sequence is shown as a transparent spacefill and ribbon model (cartoon view). Gray-colored, sdAbCSP1; Gold-colored, PfCSP-Cext; Red-colored, helix of PfCSP-Cext (aa40-52); Orange-colored, loop of PfCSP-Cext (aa83-90); Blue-colored, CDR1 (aa25-33); Cyan-colored, CDR2 (aa52-57); Purple-colored, CDR3 (aa100-118) of sdAbCSP1; Black-colored, HV4 (aa74-77) of sdAbCSP1. For the structure characterizing metrics, see structure #3 from Table 2.

**Figure S5.** Three-dimensional structure representations of the sdAbCSP1 – PfCSP-Cext complex model from HADDOCK docking. Model obtained using “backward” orientation as a start. The backbone of the amino acid sequence is shown as a transparent spacefill and ribbon model (cartoon view). Gray-colored, sdAbCSP1; Gold-colored, PfCSP-Cext; Red-colored, helix of PfCSP-Cext (aa40-52); Orange-colored, loop of PfCSP-Cext (aa83-90); Blue-colored, CDR1 (aa25-33); Cyan-colored, CDR2 (aa52-57); Purple-colored, CDR3 (aa100-118) of sdAbCSP1; Black-colored, HV4 (aa74-77) of sdAbCSP1. For the structure characterizing metrics, see structure #4 from Table 2.


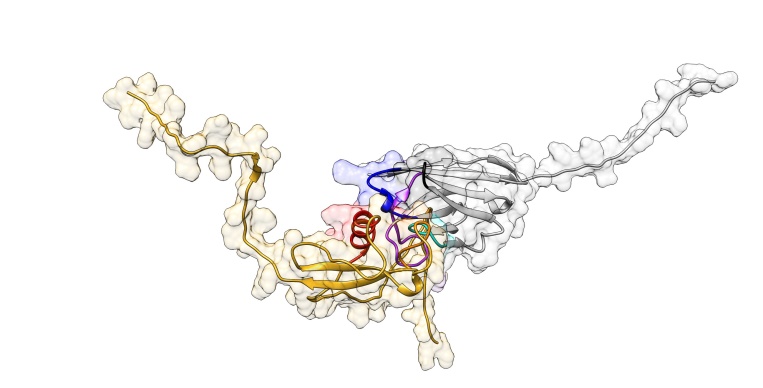


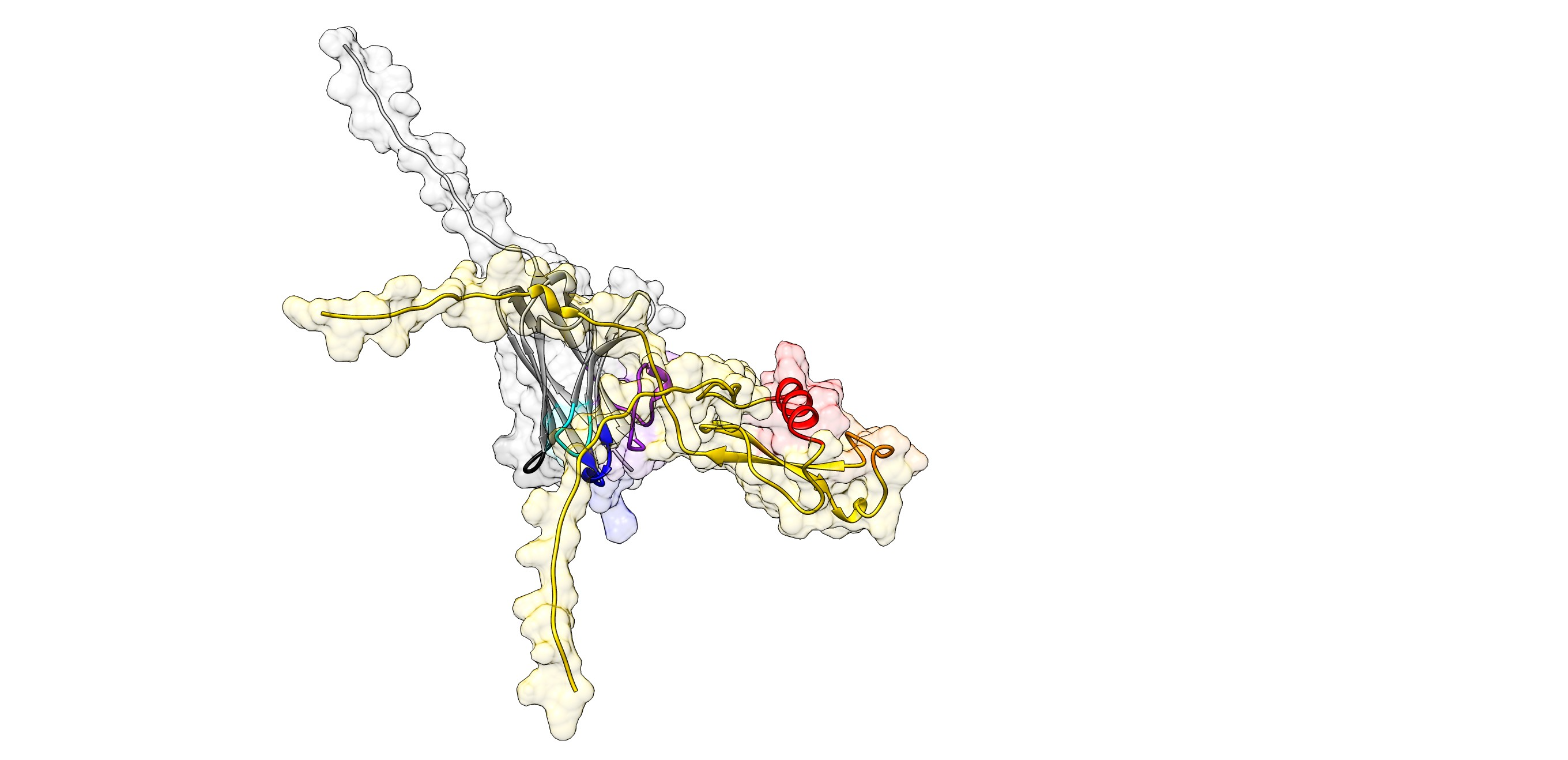


**Figure S6.** Three-dimensional structure representations of the sdAbCSP1 – PfCSP-Cext complex model from HADDOCK docking. Complex obtained using “random” configuration The backbone of the amino acid sequence is shown as a transparent spacefill and ribbon model (cartoon view). Gray-colored, sdAbCSP1; Gold-colored, PfCSP-Cext; Red-colored, helix of PfCSP-Cext (aa40-52); Orange-colored, loop of PfCSP-Cext (aa83-90); Blue-colored, CDR1 (aa25-33); Cyan-colored, CDR2 (aa52-57); Purple-colored, CDR3 (aa100-118) of sdAbCSP1; Black-colored, HV4 (aa74-77) of sdAbCSP1. For the structure characterizing metrics, see structure #5 from Table 2.

**Figure S7.** Three-dimensional structure representations of a dromedary single-domain VHH antibody fragment with RNASE A (1BZQ) from HADDOCK docking. Structure obtained using crystal structure (1BZQ) configuration. The backbone of the amino acid sequence is shown as a transparent spacefill and ribbon model (cartoon view). Gray-colored, dromedary single-domain VHH antibody fragment; Gold-colored, RNASE A; Blue-colored, CDR1 of dromedary single-domain VHH antibody fragment; Cyan-colored, CDR2 of dromedary single-domain VHH antibody fragment; Purple-colored, CDR3 of dromedary single-domain VHH antibody fragment; Black-colored, HV4 of dromedary single-domain VHH antibody fragment. For the structure characterizing metrics, see structure #7 from Table 2.


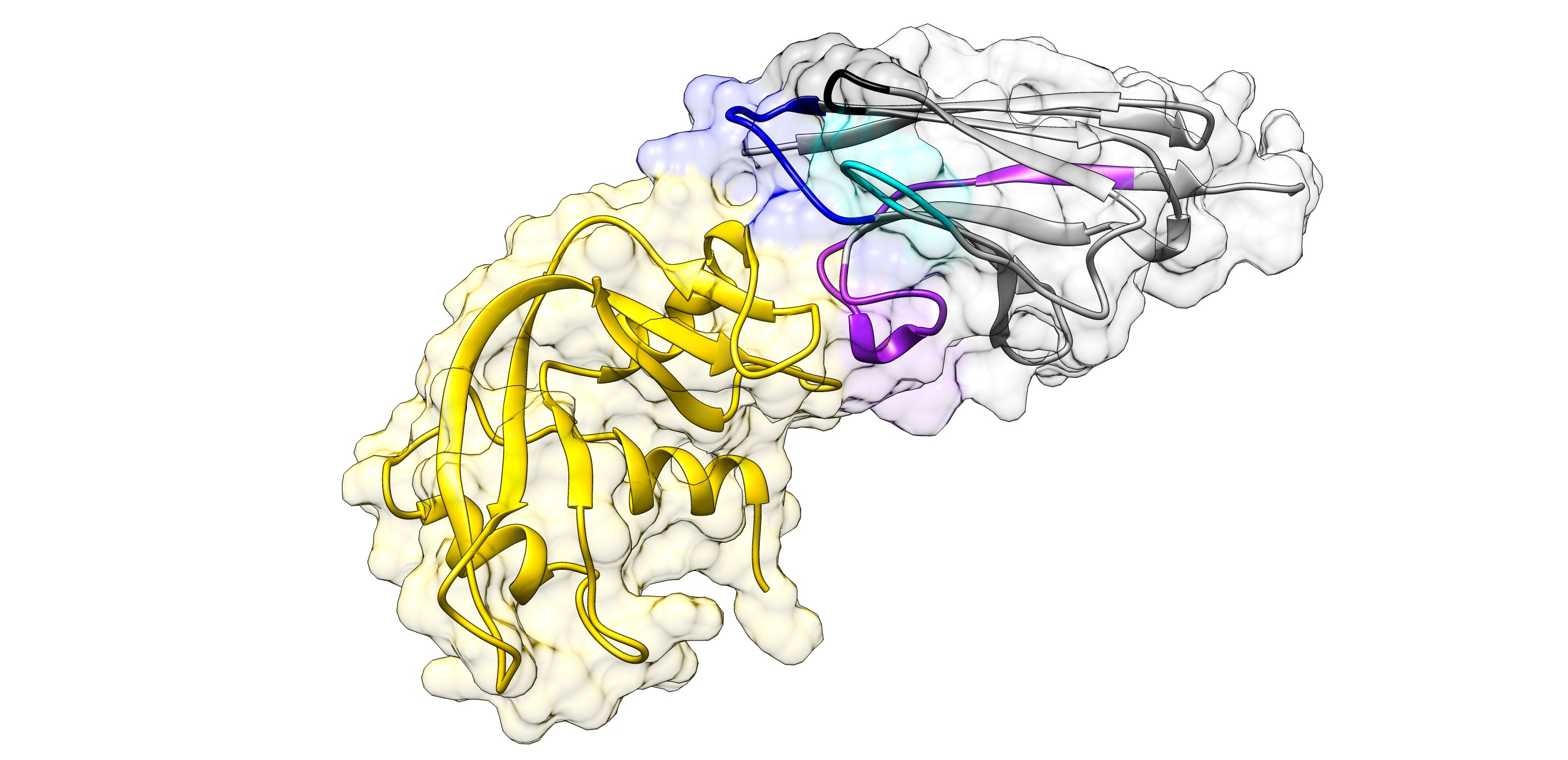


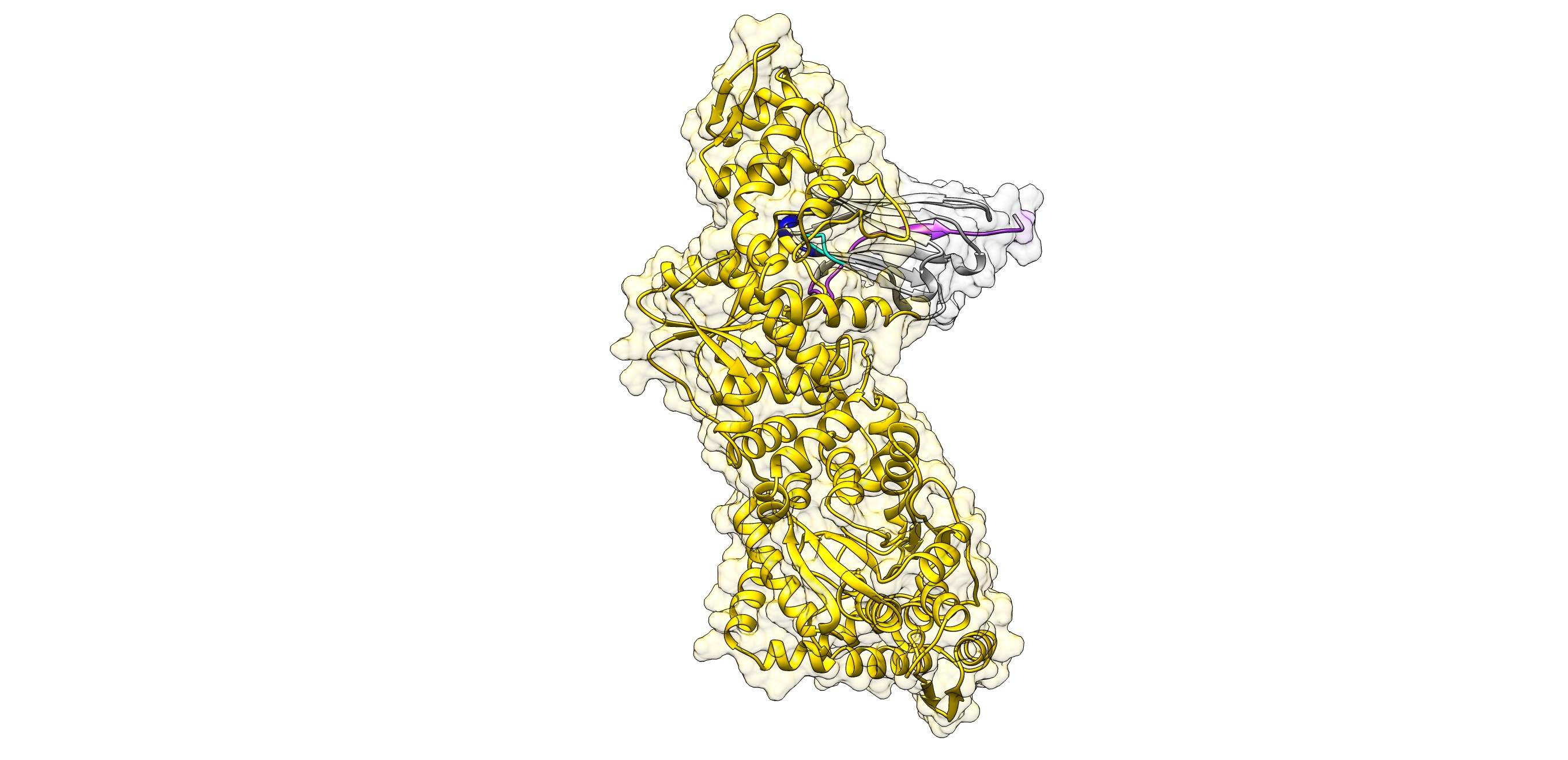


**Figure S8.** Three-dimensional structure representations of a *Leishmania donovani* tyrosyl-tRNA synthetase in complex with sdAb and inhibitor (5USF) from HADDOCK docking. Complex obtained using crystal structure (5USF) configuration. The backbone of the amino acid sequence is shown as a transparent spacefill and ribbon model (cartoon view). Gray-colored, sdAb; Gold-colored, *L. donovani* tyrosyl-tRNA synthetase; Blue-colored, CDR1 of sdAb; Cyan-colored, CDR2 of sdAb; Purple-colored, CDR3 of sdAb; Black-colored, HV4 of sdAb. For the structure characterizing metrics, see structure #8 from Table 2.


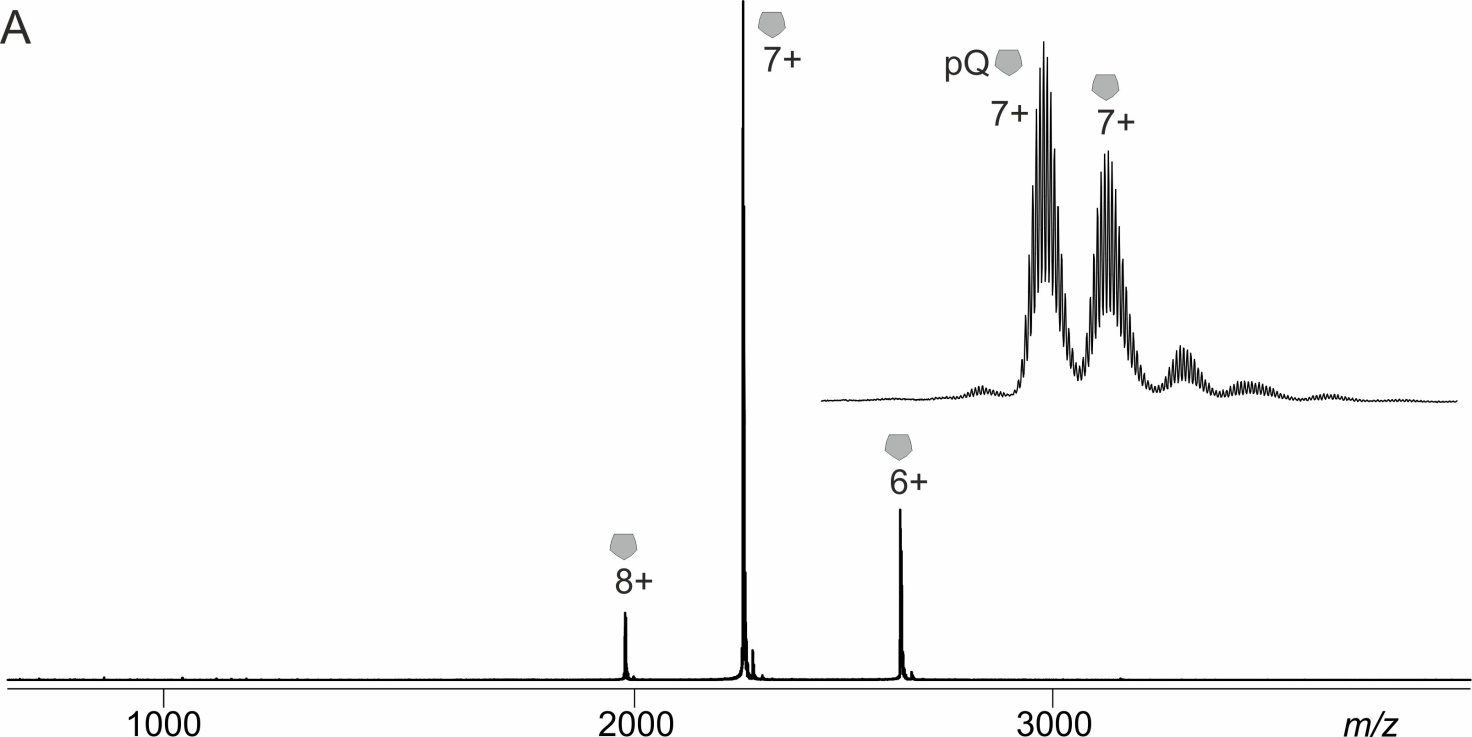


**
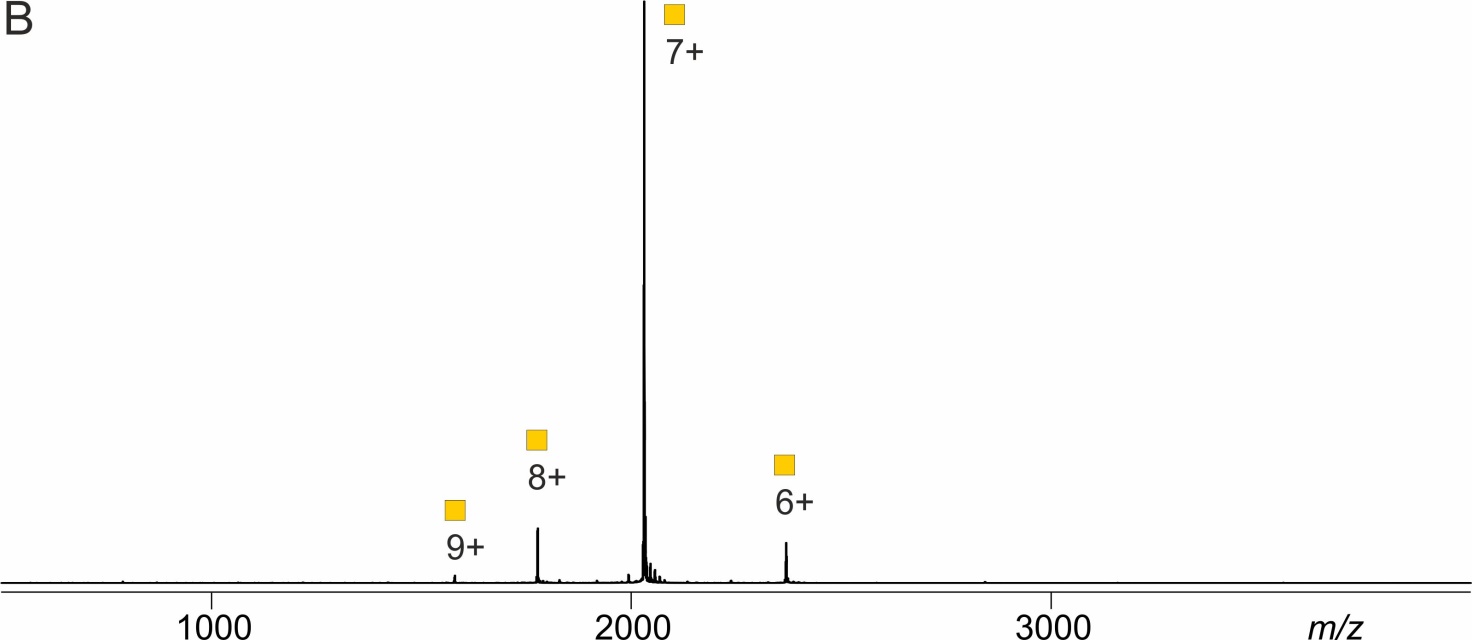
**

**Figure S9.** Offline nanoESI-MS analysis of sdAbCSP1 and PfCSP-Cext. A, Mass Spectrum of sdAbCSP1. The insert shows a zoomed view of the 7+ charged ion signal pair. pQ: ion signal of protein with N-terminal pyro-glutamine residue. B, Mass Spectrum of PfCSP-Cext. Ion signals are labeled with charge state numbers. For *m/z* values, see Tables S1, S2, and S3.


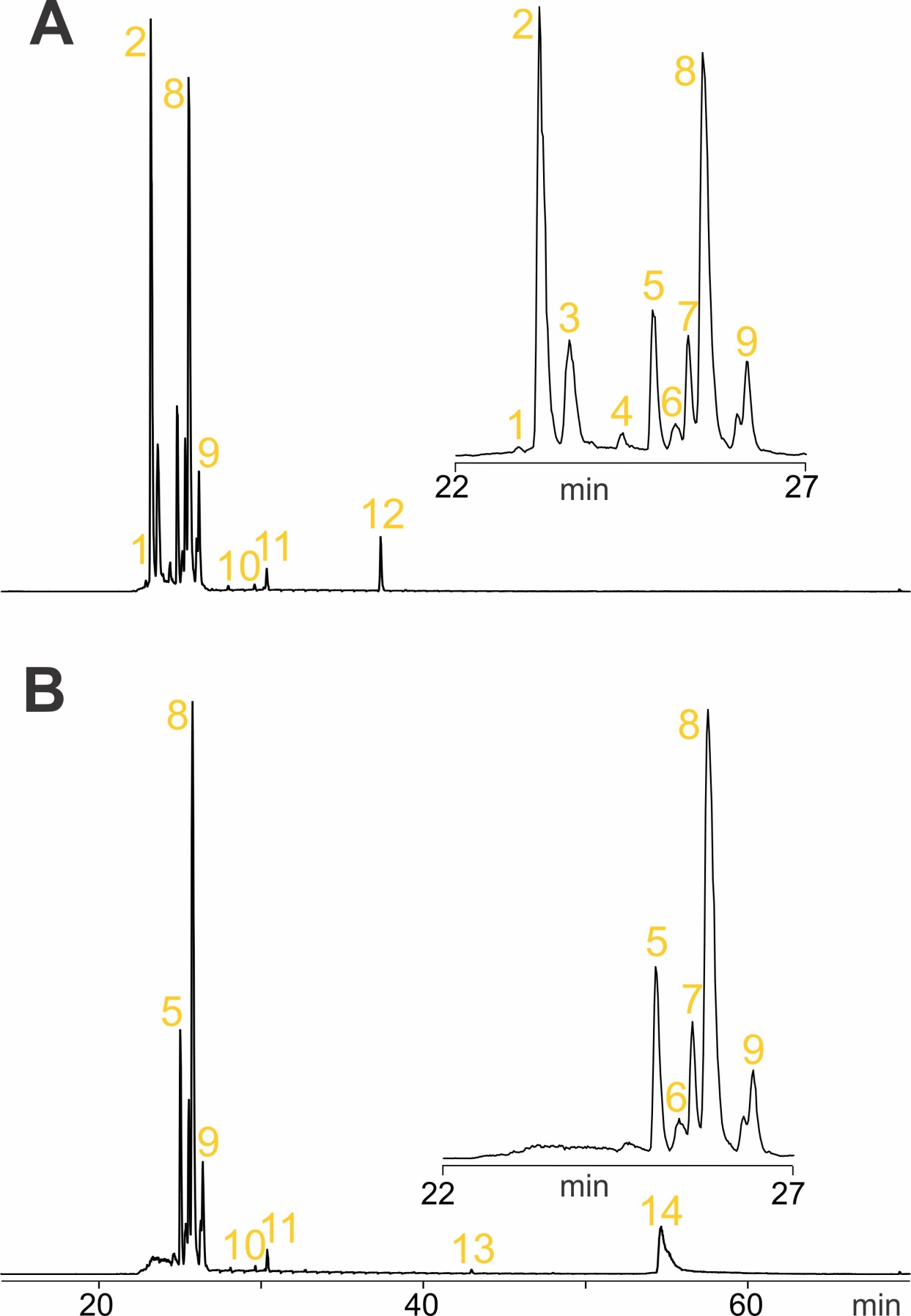


**Figure S10.** Chromatographic traces of online nanoLC-ESI-MS^E^ separated GluC-derived peptides after 72 h limited digestion. A, PfCSP-Cext; B, sdAbCSP1 – PfCSP-Cext complex (molar ratio of sdAbCSP1 : PfCSP-Cext = 1.8 : 1). Fractions are numbered according to retention times. Gold-colored fraction numbers indicate fractions that contain PfCSP-Cext peptides. For assignments of peptide partial sequences in separated fractions, see Table S5.


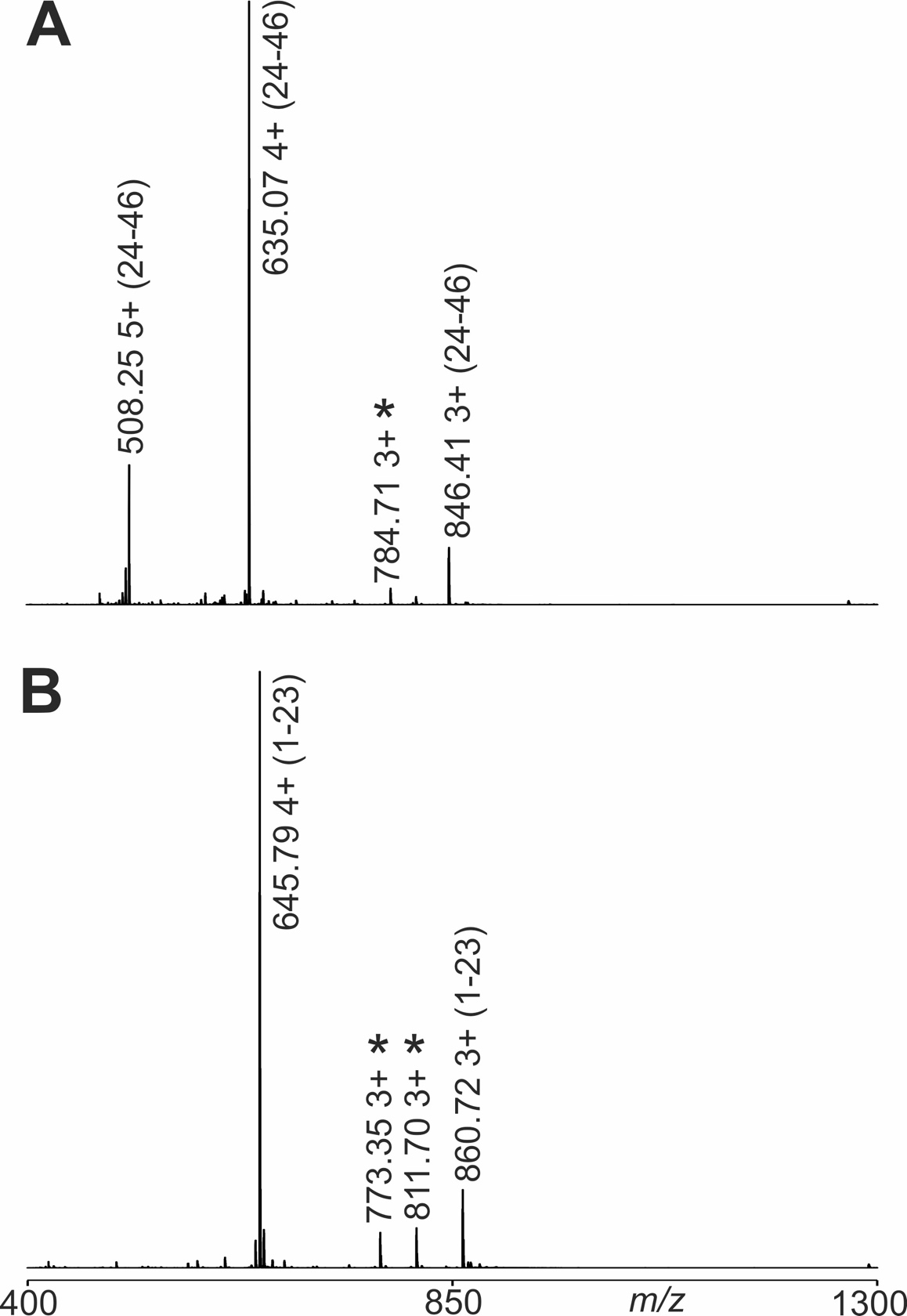


**Figure S11.** Offline nanoESI mass spectra of peptides eluting in nanoLC fractions. A, Fraction 2 of GluC digested PfCSP Cext alone; B, Fraction 5 of GluC digested sdAbCSP1 - PfCSP Cext complex. Ion signals are labeled with *m/z* values, and charge states are given. Ion signals marked with * are fragment ions. Numbers in parentheses indicate the matching PfCSP Cext amino acid sequence ranges. For ion signal assignments, see Table S5.


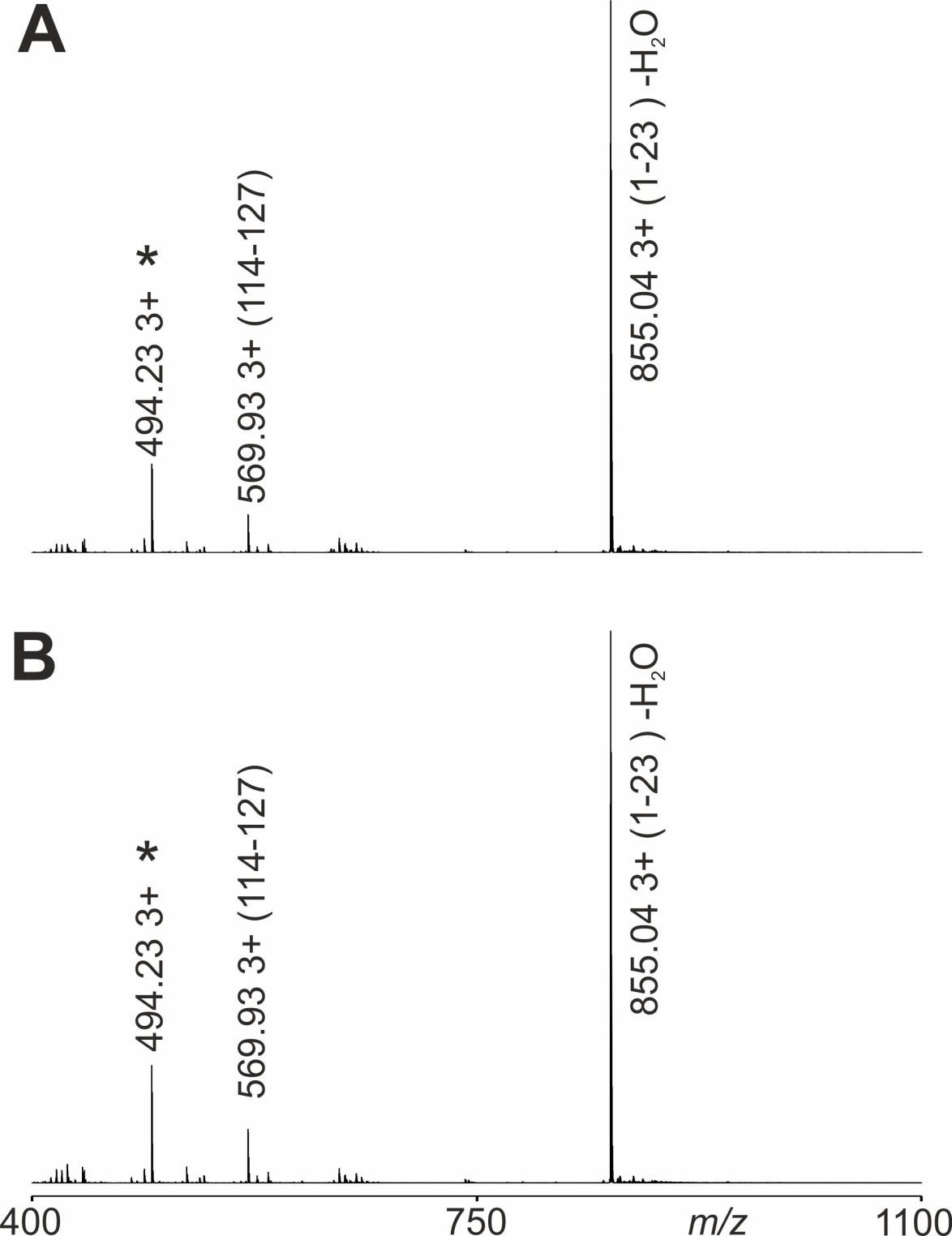


**Figure S12.** Offline nanoESI mass spectra of peptides eluting in nanoLC fractions. A, Fraction 7 of GluC digested PfCSP Cext alone. B, Fraction 7 of GluC digested sdAbCSP1 - PfCSP Cext complex. Ion signals are labeled with *m/z* values and charge states are given. Ion signals marked with * are fragment ions. Numbers in parentheses indicate the matching PfCSP Cext amino acid sequence range. For ion signal assignments, see Table S5.


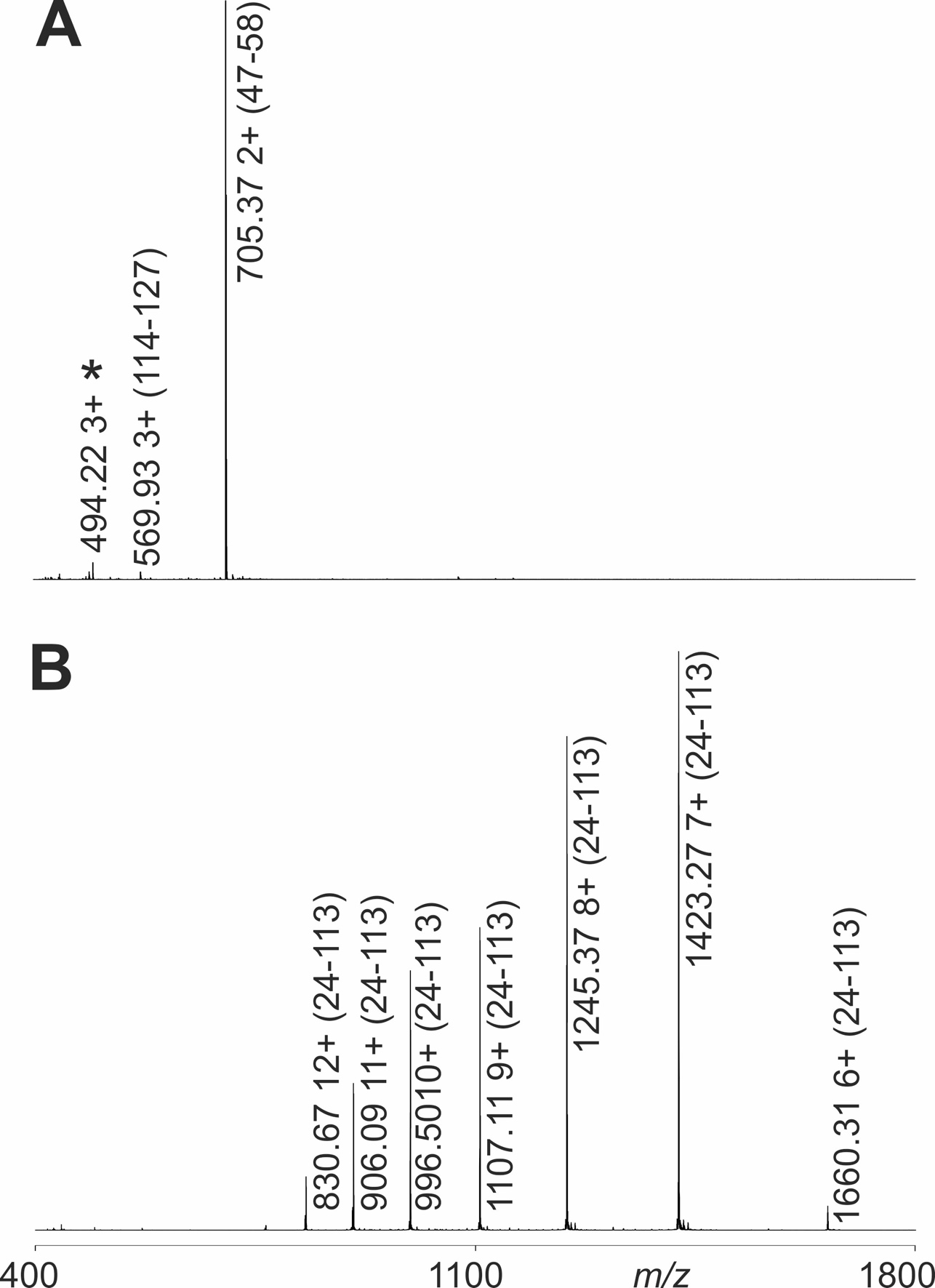


**Figure S13.** Offline nanoESI mass spectra of peptides eluting in nanoLC fractions. A, Fraction 12 of GluC digested PfCSP Cext alone; B, Fraction 14 of GluC digested sdAbCSP1 - PfCSP Cext complex. Ion signals are labeled with *m/z* values and charge states are given. Ion signals marked with * are fragment ions. Numbers in parentheses indicate the matching PfCSP Cext amino acid sequence range. For ion signal assignments, see Table S5.


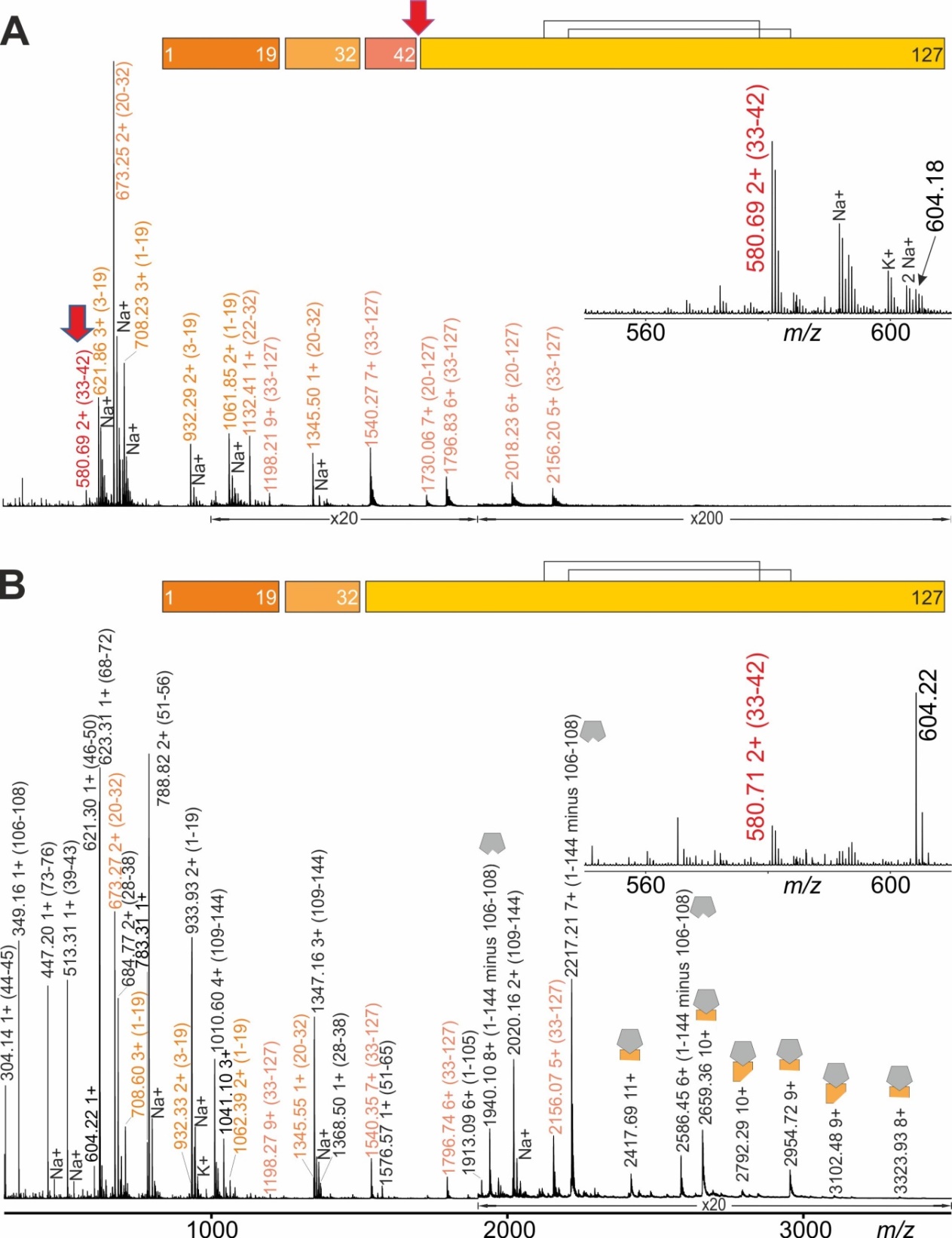


**Figure S14.** Peptide mapping by off-line nanoESI-MS^E^ analysis after 72 h limited tryptic digestion of PfCSP-Cext and of sdAbCSP1 – PfCSP-Cext complex. A, PfCSP-Cext alone; B, sdAbCSP1 – PfCSP-Cext complex with excess of sdAbCSP1 (molar ratio of sdAbCSP1 : PfCSP-Cext = 1.8 : 1). Selected ion signals are labeled with *m/z* values, and charge states for ion signals are given. Bars on top of spectra represent full-length proteins and cleavage sites are shown as gaps. Black lines represent disulfide bridges. The red arrow marks the cleavage site which was shielded by complex formation and the ion signal which proves the difference (see inserts). Solvent: 200 mM ammonium acetate, pH 6.7. For ion signal assignments, see Table S6.


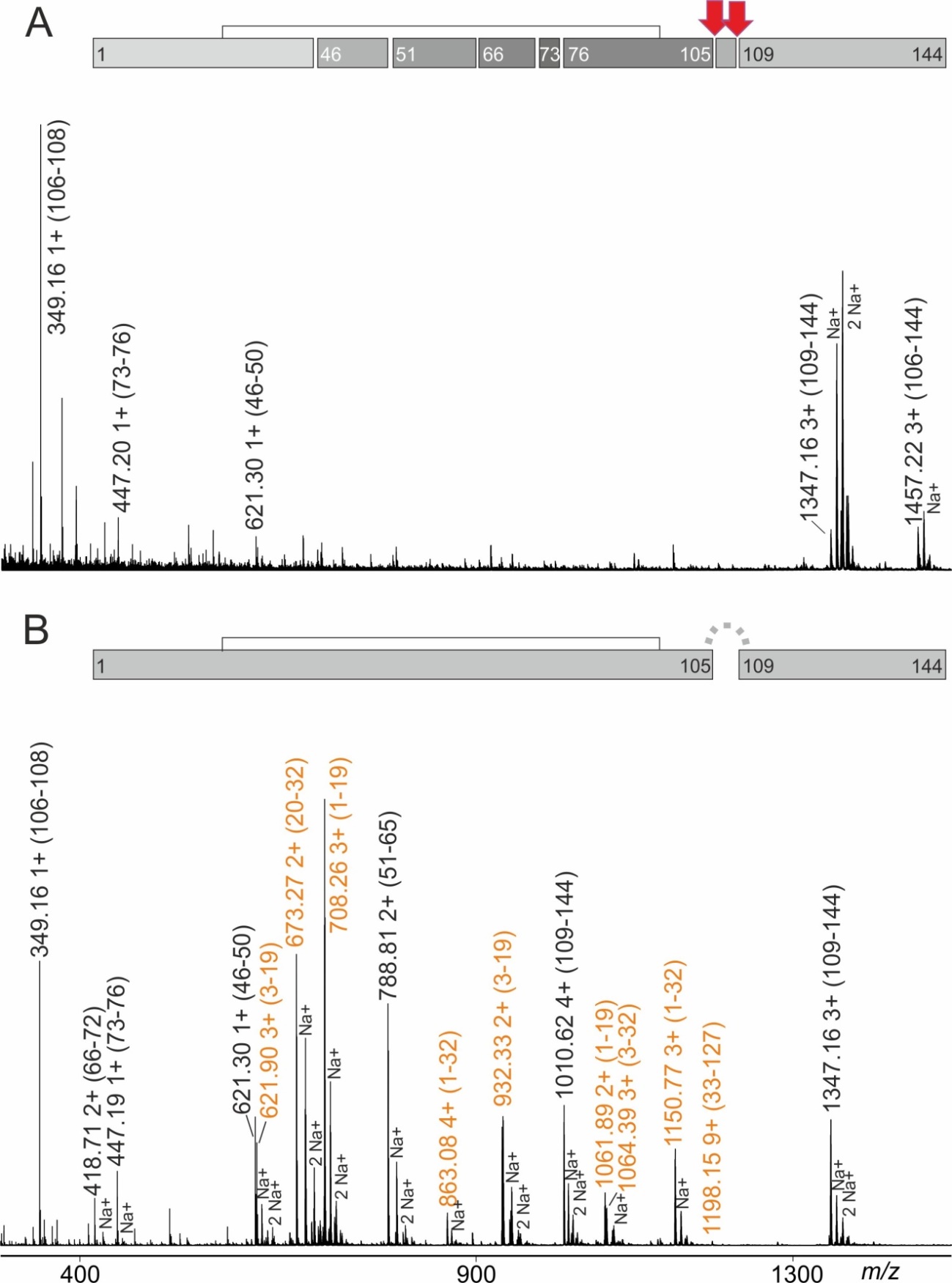


**Figure S15.** Peptide mapping by off-line nanoESI-MS^E^ analysis after 2 h limited tryptic digestion of sdAbCSP1 and of sdAbCSP1 - PfCSP Cext complex. A, sdAbCSP1 alone; B, sdAbCSP1 - PfCSP-Cext complex with excess sdAbCSP1 (molar ratio of sdAbCSP1 : PfCSP-Cext = 1.8 : 1). Selected ion signals are labeled with *m/z* values, and charge states for ion signals are given. Bars on top of spectra represent full-length or truncated proteins and cleavage sites are shown as gaps. Black lines represent disulfide bridges. The red arrows mark the cleavage sites which were shielded by complex formation. Solvent: 200 mM ammonium acetate, pH 6.7. For ion signal assignments, see Table S7.


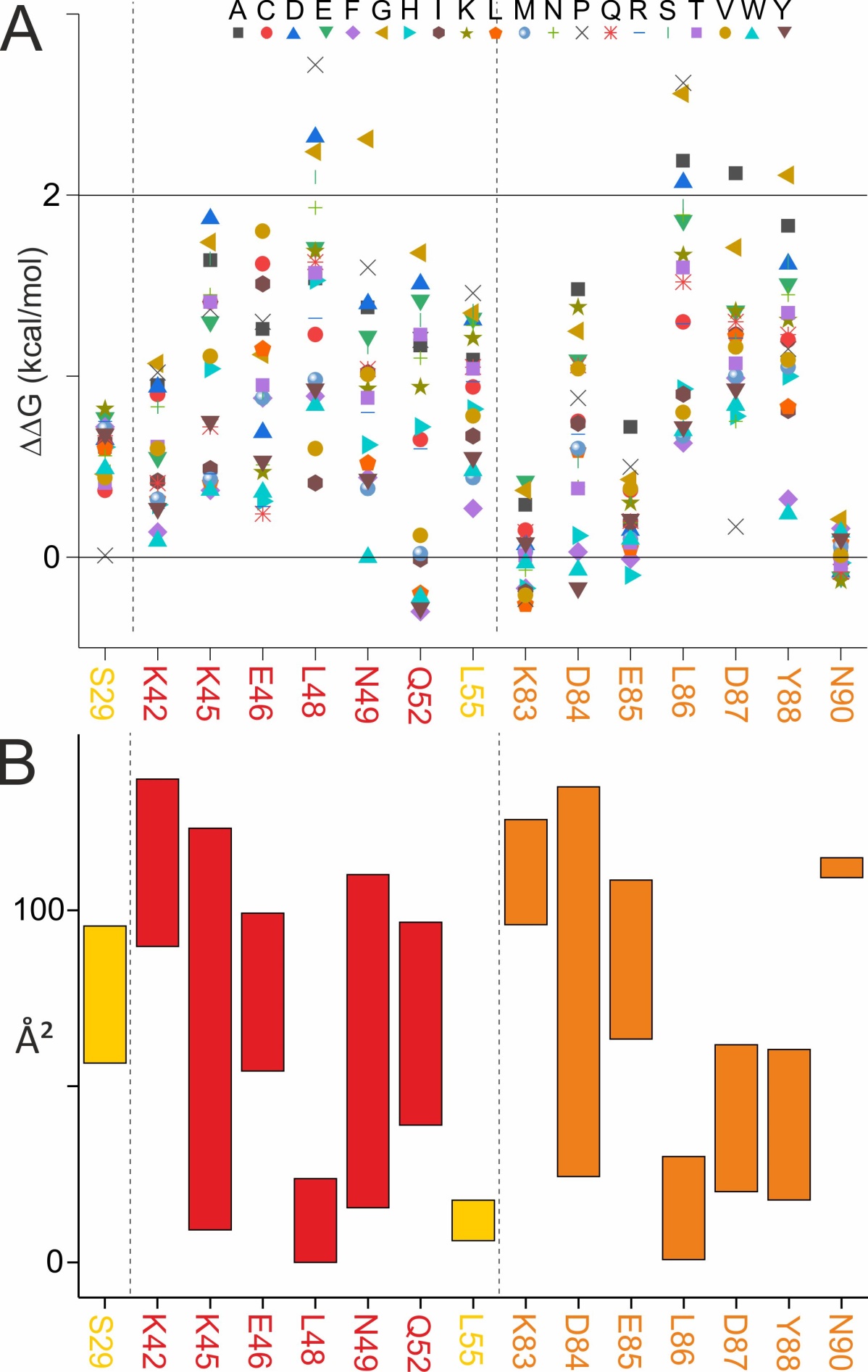


**Figure S16.** Energy penalties and surface accessibility changes accompanied with single amino acid exchanges of epitope-residing amino acid residues. **A,** Relative binding energy changes (ΔΔG values in kcal / mol) by single amino acid exchanges at each position of PfCSP-Cext, which makes contact with sdAbCSP1. The horizontal line at 2 kcal/mol marks the upper limit of the range of energy differences whose values are assumed to be negligible. **B,** Bar diagram of SASA differences. Top of the bar: SASA of the unbound PfCSP-Cext. Bottom of the bar: SASA of the sdAbCSP1 – PfCSP-Cext complex. For individual values, see Table S9 and Table S11.


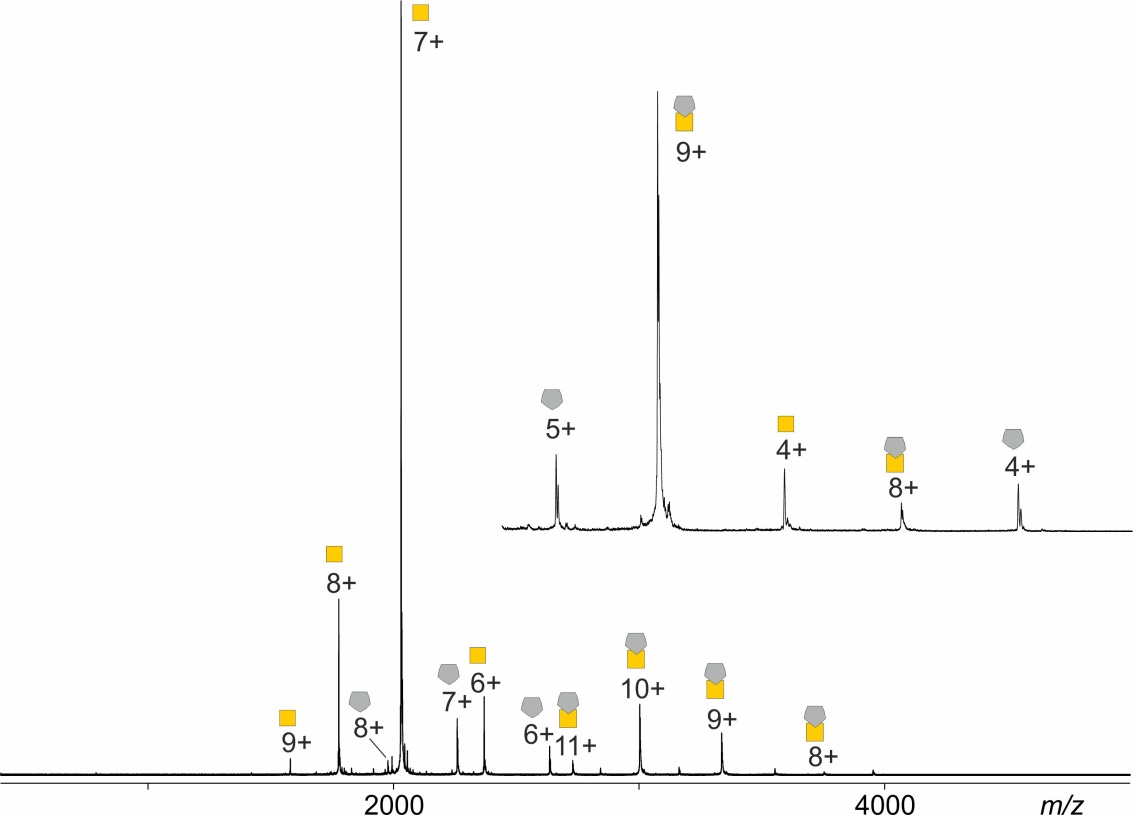


**Figure S17.** Offline nanoESI mass spectrum of sdAbCSP1 - PfCSP Cext complex prior to dissociation. Ion signals are labeled with charge states. Molar ratio of sdAbCSP1 : PfCSP-Cext = 1 : 3.4). The zoomed region shows ion signals from *m/z* 3300 to *m/z* 4000. Orange colored square & green colored pentagon, complex; Orange colored square, PfCSP Cext; Green colored pentagon, sdAbCSP1. Solvent: 200 mM ammonium acetate, pH 6.7.


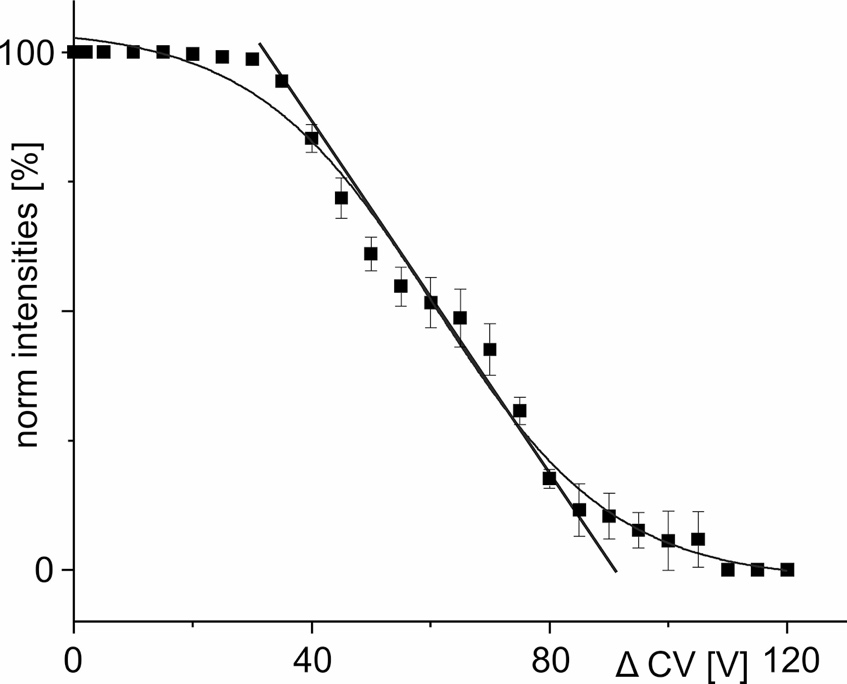


**Figure S18.** Boltzmann plot for the gas phase complex dissociation reaction of the sdAbCSP1 - PfCSP Cext complex. The course of normalized educt ion intensities (average of three independent measurement series) is shown as a function of ΔCV. Complexes consisted of i) sdAbCSP1 - PfCSP Cext and ii) pQ-sdAbCSP1 - PfCSP Cext. Data points with standard deviations (vertical bars) are the means of three measurements each. The tangent line indicates the linear dependency of the complex dissociation reaction in the gas phase with respect to ΔCV. For curve parameters, see Table S16.


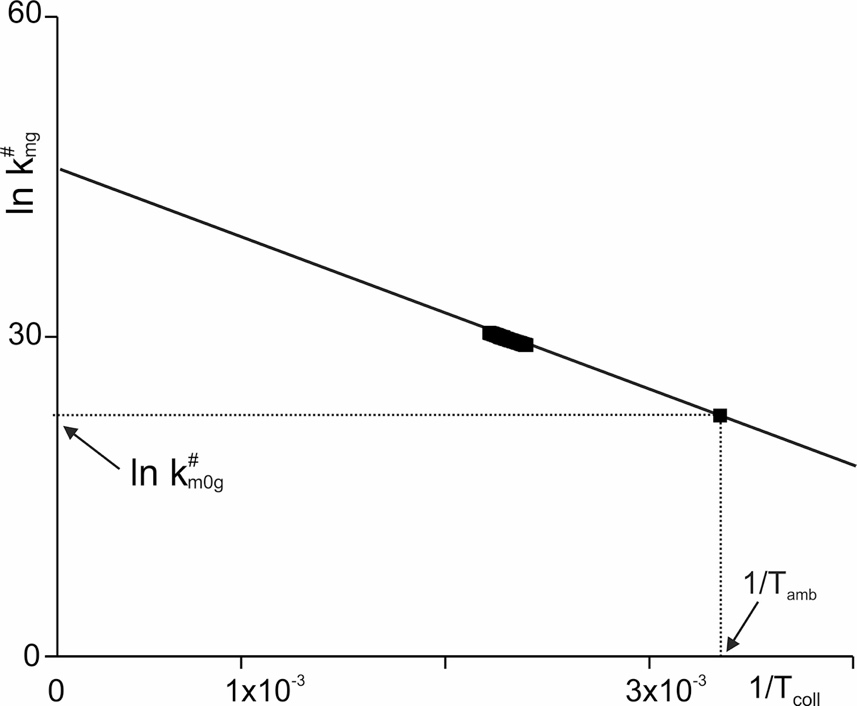


**Figure S19.** Arrhenius plot for the gas phase complex dissociation reaction of sdAbCSP1 - PfCSP Cext complex. Each data point (thickened part of the line) was obtained from the linear part of the Boltzmann curve (cf. Figure S17) and the resulting line was extrapolated in both directions. The value for ${ln k}_{D m0g}^{\#}$ corresponds to $\frac{1}{T_{\mathrm{amb}}}$. Calculated values are listed in Table 3.


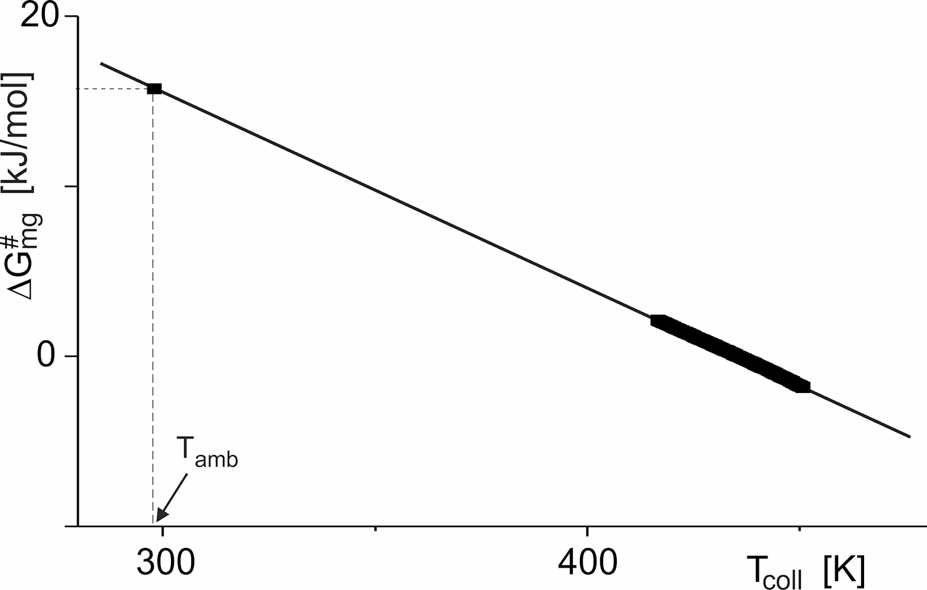


**Figure S20.** Experimental ΔG vs. T plot for the gas phase complex dissociation reaction of sdAbCSP1 - PfCSP Cext complex. Each data point (thickened part of the line) was obtained from the linear part of the Boltzmann curve and the resulting line was extrapolated in both directions. The value for ${\Delta G}_{m0g}^{\#}$ corresponds to $\frac{1}{T_{\mathrm{amb}}}$. Calculated values are listed in Table 3.

# Supplemental Tables

**Table S1.** Offline nanoESI-MS analysis of sdAbCSP1.

| ***m/z* (exp.)** | **z (exp.)** | **M (avg.)** | **std. dev.** | **M_r_ (avg.)** |
| --- | --- | --- | --- | --- |
| 1979.29 | 8 | 15,826.32 |  |  |
| 2261.90 | 7 | 15,826.30 |  |  |
| 2638.72 | 6 | 15,826.32 |  |  |
|  | Ø | 15,826.31 | 0.01 | 15,826.17 |

**Table S2.** Offline nanoESI-MS analysis of pQ-sdAbCSP1.

| ***m/z* (exp.)** | **z (exp.)** | **M (avg.)** | **std. dev.** | **M_r_ (avg.)** |
| --- | --- | --- | --- | --- |
| 1977.03 | 8 | 15,808.24 |  |  |
| 2259.33 | 7 | 15,808.31 |  |  |
| 2635.70 | 6 | 15,808.20 |  |  |
|  | Ø | 15,808.25 | 0.06 | 15808,15 |

**Table S3.** Offline nanoESI-MS analysis of PfCSP-Cext.

| ***m/z* (exp.)** | **z (exp.)** | **M (avg.)** | **std. dev.** | **M_r_ (avg.)** |
| --- | --- | --- | --- | --- |
| 1579.84 | 9 | 14,209.56 |  |  |
| 1777.18 | 8 | 14,209.44 |  |  |
| 2030.89 | 7 | 14,209.23 |  |  |
| 2369.24 | 6 | 14,209,44 |  |  |
| 2842.90 | 5 | 14,209.50 |  |  |
| 3553.32 | 4 | 14,209.28 |  |  |
|  | Ø | 14,209.41 | 0.13 | 14,208.36 |

**Table S4.** Peptide masses of PfCSP-Cext and sdAbCSP1 – PfCSP-Cext complex after 24 h Glu-C digest recorded by offline nanoESI-MS^E^ analysis.

| **sequence range** | **M+H^+^** | **z** |  | ***m/z* (exp.)** | |  | **comment** |
| --- | --- | --- | --- | --- | --- | --- | --- |
| **sdAbCSP1** | **(calcd)** | **(exp.)** |  | **PfCSP-Cext** | **complex** |  |  |
| (1-144) | 15827.17 ^a)^ | 9 |  | / | 1759.44 |  |  |
|  | 15827.17 ^a)^ | 8 |  | / | 1979.47 |  |  |
|  | 15827.17 ^a)^ | 7 |  | / | 2262.12 |  |  |
|  | 15827.17 ^a)^ | 6 |  | / | 2639.15 |  |  |
|  | 15810.14 ^a)^ | 10 |  | / | 1581.69 |  | pyroQ |
|  | 15810.14 ^a)^ | 9 |  | / | 1757.32 |  | pyroQ |
|  | 15810.14 ^a)^ | 8 |  | / | 1976.86 |  | pyroQ |
|  | 15810.14 ^a)^ | 7 |  | / | 2259.12 |  | pyroQ |
|  | 15810.14 ^a)^ | 6 |  | / | 2635.48 |  | pyroQ |
|  | 15849.17 ^a)^ | 8 |  | / | 1981.86 |  | +Na^+^ |
|  | 15849.17 ^a)^ | 7 |  | / | 2264.84 |  | +Na^+^ |
|  | 15849.17 ^a)^ | 6 |  | / | 2641.97 |  | +Na^+^ |

**Table S4.** Peptide masses of PfCSP-Cext and sdAbCSP1 – PfCSP-Cext complex after 24 h Glu-C digest recorded by offline nanoESI-MS^E^ analysis (continued).

| **sequence range** | **M+H^+^** | **z** |  | ***m/z* (exp.)** | |  | **comment** |
| --- | --- | --- | --- | --- | --- | --- | --- |
| **PfCSP-Cext** | **(calcd)** | **(exp.)** |  | **PfCSP-Cext** | **complex** |  |  |
| 1-23 | 2580.11 | 3 |  | 860.61 | 860.61 |  |  |
|  | 2580.11 | 2 |  | 1290.40 | 1290.41 |  |  |
|  | 2581.11 | 3 |  | 860.94 | 860.94 |  | 1 deamid |
|  | 2581.11 | 2 |  | 1290.90 | 1290.91 |  | 1 deamid |
|  | 2603.11 | 3 |  | 868.26 | 868.27 |  | 1 deamid, +Na^+^ |
|  | 2603.11 | 2 |  | 1301.88 | 1301.90 |  | 1 deamid, +Na^+^ |
|  | 2619.11 | 3 |  | 873.58 | 873.60 |  | 1 deamid, +K^+^ |
|  | 2619.11 | 2 |  | 1309.87 | 1309.88 |  | 1 deamid, +K^+^ |
|  | 2625.11 | 3 |  | 875.59 | 875.59 |  | 1 deamid, +2Na^+^ |
|  | 2625.11 | 2 |  | 1312.88 | 1312.88 |  | 1 deamid, +2Na^+^ |
| 1-46 | 5102.28 ^a)^ | 5 |  | 1021.33 | / |  | 1 deamid |
|  | 5102.28 ^a)^ | 4 |  | 1276.41 | 1276.43 |  | 1 deamid |
|  | 5124.28 ^a)^ | 5 |  | 1025.53 | / |  | 1 deamid, +Na^+^ |
| 24-46 | 2537.20 | 4 |  | 634.98 | / |  |  |
|  | 2537.20 | 3 |  | 846.30 | / |  |  |
|  | 2537.20 | 2 |  | 1268.95 | / |  |  |
|  | 2559.20 | 4 |  | 640.47 | / |  | +Na^+^ |
|  | 2559.20 | 3 |  | 853.63 | / |  | +Na^+^ |
|  | 2575.20 | 4 |  | 644.46 | / |  | +K^+^ |
|  | 2575.20 | 3 |  | 858.95 | / |  | +K^+^ |
| 47-58 | 1409.73 | 2 |  | 705.28 | / |  |  |
|  | 1431.73 | 2 |  | 716.27 | / |  | +Na^+^ |
|  | 1447.73 | 2 |  | 724.26 | / |  | +K^+^ |
| 1-113 | 12519.61 ^a)^ | 7 |  | 1789.49 | 1789.51 |  |  |
|  | 12541.61 ^a)^ | 7 |  | 1792.35 | / |  | +Na^+^ |
|  | 12557.61 ^a)^ | 7 |  | 1795.06 | / |  | +K^+^ |
| 24-113 | 9956.94 ^a)^ | 7 |  | 1423.09 | 1423.10 |  |  |
|  | 9956.94 ^a)^ | 6 |  | 1660.11 | 1660.13 |  |  |
|  | 9978.94 ^a)^ | 7 |  | 1426.23 | 1426.25 |  | +Na^+^ |
|  | 9978.94 ^a)^ | 6 |  | 1663.77 | 1663.79 |  | +Na^+^ |
| 47-113 | 7437.34 ^a)^ | 5 |  | 1487.96 | / |  |  |
|  | 7459.34 ^a)^ | 5 |  | 1492.35 | / |  | +Na^+^ |
| 114-127 | 1707.76 | 3 |  | 589.86 | 569.87 |  |  |
|  | 1707.76 | 2 |  | 854.29 | 854.29 |  |  |
|  | 1729.76 | 3 |  | 577.18 | 577.19 |  | +Na^+^ |
|  | 1745.76 | 3 |  | 582.50 | 582.51 |  | +K^+^ |
|  | 1751.76 | 3 |  | 584.51 | 584.51 |  | +2Na^+^ |

**Table S4.** Peptide masses of PfCSP Cext and sdAbCSP1 – PfCSP-Cext complex after 24 h Glu-C digest recorded by offline nanoESI-MS^E^ analysis (continued).

| **sequence range** | **M+H^+^** | **z** |  | ***m/z* (exp.)** | |  | **comment** |
| --- | --- | --- | --- | --- | --- | --- | --- |
| **complex** | **(calcd)** | **(exp.)** |  | **PfCSP-Cext** | **complex** |  |  |
| (1-144) + (24-113) | 25783.11 ^a)^ | 12 |  | / | 2149.70 |  |  |
|  | 25783.11 ^a)^ | 11 |  | / | 2344.94 |  |  |
|  | 25783.11 ^a)^ | 10 |  | / | 2579.43 |  |  |
|  | 25783.11 ^a)^ | 9 |  | / | 2865.83 |  |  |
|  | 25783.11 ^a)^ | 8 |  | / | 3223.97 |  |  |
|  | 25766.08 ^a)^ | 12 |  | / | 2147.79 |  | pyroQ |
|  | 25766.08 ^a)^ | 11 |  | / | 2343.04 |  | pyroQ |
|  | 25766.08 ^a)^ | 10 |  | / | 2577.26 |  | pyroQ |
|  | 25766.08 ^a)^ | 9 |  | / | 2863.57 |  | pyroQ |
|  | 25766.08 ^a)^ | 8 |  | / | 3221.30 |  | pyroQ |
|  | 25805.11 ^a)^ | 11 |  | / | 2346.67 |  | +Na^+^ |
|  | 25805.11 ^a)^ | 10 |  | / | 2581.35 |  | +Na^+^ |
|  | 25805.11 ^a)^ | 9 |  | / | 2867.91 |  | +Na^+^ |
|  | 25805.11 ^a)^ | 8 |  | / | 3226.30 |  | +Na^+^ |
|  | 25827.11 ^a)^ | 11 |  | / | 2348.60 |  | +2Na^+^ |
|  | 25827.11 ^a)^ | 10 |  | / | 2583.26 |  | +2Na^+^ |
|  | 25827.11 ^a)^ | 9 |  | / | 2870.24 |  | +2Na^+^ |
|  | 25827.11 ^a)^ | 8 |  | / | 3229.02 |  | +2Na^+^ |

1. average mass

**Table S5.** Peptide masses of PfCSP-Cext and sdAbCSP1 – PfCSP-Cext complex after 72 h GluC digestion recorded by nanoLC ESI-MS^E^ analysis.

|  | ret. | *m/z* | *m/z* |  |  | **PfCSP-Cext** |  | **complex** |  |  |
| --- | --- | --- | --- | --- | --- | --- | --- | --- | --- | --- |
| no. | time | (exp.) | (calcd) | z |  | sequence range |  | sequence range |  | comment |
| **1** | **22.90** | 585.27 | 585.26 | 2 |  | 1-23 |  |  |  | oxM; y"10 |
|  |  | **649.79** | **649.78** | **4** |  | **1-23** |  |  |  | **oxM** |
|  |  | 778.68 | 778.68 | 3 |  | 1-23 |  |  |  | oxM; b21 |
|  |  | 817.02 | 817.03 | 3 |  | 1-23 |  |  |  | oxM; y"22/b22 |
|  |  | 866.05 | 866.04 | 3 |  | 1-23 |  |  |  | oxM |
| **2** | **23.20** | 508.25 | 508.25 | 5 |  | 24-46 |  |  |  |  |
|  |  | **635.07** | **635.05** | **4** |  | **24-46** |  |  |  |  |
|  |  | 846.41 | 846.40 | 3 |  | 24-46 |  |  |  |  |
| **3** | **23.63** | 508.44 | 508.44 | 5 |  | 24-46 |  |  |  | 1 deamid |
|  |  | **635.31** | **635.30** | **4** |  | **24-46** |  |  |  | **1 deamid** |
|  |  | 650.03 | 650.03 | 4 |  | 1-23 |  |  |  | 1 deamid; oxM |
|  |  | 846.74 | 846.73 | 3 |  | 24-46 |  |  |  | 1 deamid |
| **4** | **24.39** | 508.44 | 508.44 | 5 |  | 24-46 |  |  |  | 1 deamid |
|  |  | **635.31** | **635.30** | **4** |  | **24-46** |  |  |  | **1 deamid** |
|  |  | **649.79** | **649.78** | **4** |  | **1-23** |  |  |  | **oxM** |
|  |  | 846.74 | 846.73 | 3 |  | 24-46 |  |  |  | 1 deamid |
|  |  | 866.05 | 866.04 | 3 |  | 1-23 |  |  |  | oxM |
| **5** | **24.82** | **645.79** | **645.78** | **4** |  | **1-23** |  | **1-23** |  |  |
|  |  | 773.35 | 773.35 | 3 |  | 1-23 |  | 1-23 |  | b21 |
|  |  | 811.70 | 811.69 | 3 |  | 1-23 |  | 1-23 |  | b22 |
|  |  | 860.71 | 860.71 | 3 |  | 1-23 |  | 1-23 |  |  |
| **6** | **25.14** | **650.03** | **650.03** | **4** |  | **1-23** |  | **1-23** |  | **1 deamid; oxM** |
|  |  | 866.37 | 866.37 | 3 |  | 1-23 |  | 1-23 |  | 1 deamid; oxM |
| **7** | **25.33** | **646.04** | **646.03** | **4** |  | **1-23** |  | **1-23** |  | **1 deamid** |
|  |  | 773.68 | 773.67 | 3 |  | 1-23 |  | 1-23 |  | 1 deamid; b21 |
|  |  | 812.02 | 812.02 | 3 |  | 1-23 |  | 1-23 |  | 1 deamid; b22 |
|  |  | 861.04 | 861.03 | 3 |  | 1-23 |  | 1-23 |  | 1 deamid |
| **8** | **25.53** | **646.04** | **646.03** | **4** |  | **1-23** |  | **1-23** |  | **1 deamid** |
|  |  | 773.68 | 773.67 | 3 |  | 1-23 |  | 1-23 |  | 1 deamid; b21 |
|  |  | 812.02 | 812.02 | 3 |  | 1-23 |  | 1-23 |  | 1 deamid; b22 |
|  |  | 861.04 | 861.03 | 3 |  | 1-23 |  | 1-23 |  | 1 deamid |
| **9** | **26.16** | **646.28** | **646.27** | **4** |  | **1-23** |  | **1-23** |  | **2 deamid** |
|  |  | 774.01 | 774.00 | 3 |  | 1-23 |  | 1-23 |  | 2 deamid; b21 |
|  |  | 812.35 | 812.34 | 3 |  | 1-23 |  | 1-23 |  | 2 deamid; b22 |
|  |  | 861.37 | 861.36 | 3 |  | 1-23 |  | 1-23 |  | 2 deamid |
| **10** | **29.60** | 494.23 | 494.22 | 3 |  | 1-23 |  | 1-23 |  | a10 |
|  |  | 569.93 | 569.93 | 3 |  | 114-127 |  | 114-127 |  |  |
|  |  | **854.71** | **854.70** | **3** |  | **1-23** |  | **1-23** |  | **-H_2_O** |
| **11** | **30.35** | 494.23 | 494.22 | 3 |  | 1-23 |  | 1-23 |  | a10 |
|  |  | 569.93 | 569.93 | 3 |  | 114-127 |  | 114-127 |  |  |
|  |  | **855.04** | **855.03** | **3** |  | **1-23** |  | **1-23** |  | **1 deamid; -H_2_O** |
| **12** | **37.36** | **705.37** | **705.37** | **2** |  | **47-58** |  |  |  |  |
|  |  | 569.93 | 569.93 | 3 |  | 114-127 |  |  |  |  |
| **13** | **43.72** | 569.93 | 569.93 | 3 |  |  |  | 114-127 |  |  |
|  |  | **684.34** | **684.33** | **1** |  |  |  | **114-118** |  |  |
| **14** | **44.40** | 830.67 | 830.66 | 12 |  |  |  | 24-113 |  |  |
|  |  | 906.09 | 906.08 | 11 |  |  |  | 24-113 |  |  |
|  |  | 996.50 | 996.59 | 10 |  |  |  | 24-113 |  |  |
|  |  | 1107.11 | 1107.21 | 9 |  |  |  | 24-113 |  |  |
|  |  | 1245.37 | 1245.49 | 8 |  |  |  | 24-113 |  |  |
|  |  | 1423.27 | 1423.28 | 7 |  |  |  | 24-113 |  |  |

**Table S6.** Peptide masses of sdAbCSP1 and sdAbCSP1 – PfCSP-Cext complex after 72h tryptic digestion recorded by offline nanoESI-MS^E^ analysis.

| **sequence range** | **M+H^+^** | **z** |  | ***m/z* (exp.)** | |  | **comment** |
| --- | --- | --- | --- | --- | --- | --- | --- |
| **sdAbCSP1** | **(calcd)** | **(exp.)** |  | **sdAbCSP1** | **complex** |  |  |
| 1-19 | 1883.99 | 2 |  | 942.41 | 942.43 |  |  |
|  | 1866.97 | 1 |  | 1866.77 | 1866.77 |  | pyroQ |
|  | 1866.97 | 2 |  | 933.89 | 933.93 |  | pyroQ |
|  | 1888.97 | 2 |  | 944.88 | 944.91 |  | pyroQ, +Na^+^ |
|  | 1904.97 | 2 |  | 952.86 | 952.90 |  | pyroQ, +K^+^ |
| 28-38 | 1368.60 | 1 |  | 1368.45 | 1368.50 |  |  |
|  | 1368.60 | 2 |  | 684.73 | 684.77 |  |  |
|  | 1390.60 | 1 |  | 1390.43 | 1390.50 |  | +Na^+^ |
|  | 1390.60 | 2 |  | 695.72 | 695.76 |  | +Na^+^ |
|  | 1406.60 | 2 |  | 703.71 | 703.71 |  | +K^+^ |
| 39-43 | 513.34 | 1 |  | 513.29 | 513.31 |  |  |
|  | 535.34 | 1 |  | 535.27 | 535.29 |  | +Na^+^ |
|  | 531.34 | 1 |  | 551.24 | 551.26 |  | +K^+^ |
|  | 557.34 | 1 |  | 557.25 | 557.27 |  | +2Na^+^ |
| 39-45 | 798.48 | 2 |  | 399.70 | 399.72 |  |  |
|  | 820.48 | 2 |  | 410.70 | 410.70 |  | +Na^+^ |
| 44-45 | 304.16 | 1 |  | 304.13 | 304.14 |  |  |
| 44-50 | 906.48 | 2 |  | 453.70 | 453.72 |  |  |
|  | 928.48 | 2 |  | 464.69 | 464.71 |  | +Na^+^ |
| 46-50 | 621.34 | 1 |  | 621.28 | 621.30 |  |  |
|  | 643.34 | 1 |  | 643.26 | 643.28 |  | +Na^+^ |
|  | 659.34 | 1 |  | 659.21 | 659.25 |  | +K^+^ |
| 51-65 | 1576.72 | 1 |  | 1576.57 | 1576.57 |  |  |
|  | 1576.72 | 2 |  | 788.78 | 788.82 |  |  |
|  | 1598.72 | 1 |  | 1598.53 | 1598.53 |  | +Na^+^ |
|  | 1598.72 | 2 |  | 799.77 | 799.81 |  | +Na^+^ |
|  | 1614.72 | 2 |  | 807.75 | 807.79 |  | +K^+^ |
|  | 1620.72 | 2 |  | 810.77 | 810.79 |  | +2Na^+^ |
| 51-67 | 1789.85 | 2 |  | 895.33 | 895.37 |  |  |
|  | 1811.85 | 2 |  | 906.38 | 906.42 |  | +Na^+^ |
| 66-72 | 836.47 | 1 |  | 836.39 | 836.42 |  |  |
|  | 836.47 | 2 |  | 418.70 | 418.71 |  |  |
|  | 858.47 | 2 |  | 429.69 | 429.70 |  | +Na^+^ |
| 68-72 | 623.35 | 1 |  | 623.29 | 623.31 |  |  |
|  | 645.35 | 1 |  | 645.27 | 645.29 |  | +Na^+^ |
| 68-76 | 1051.55 | 2 |  | 526.22 | 526.24 |  |  |
| 73-76 | 447.12 | 1 |  | 447.18 | 447.20 |  |  |
|  | 469.12 | 1 |  | 469.15 | 469.17 |  | +Na^+^ |
|  | 485.12 | 1 |  | 485.11 | 485.13 |  | +K^+^ |
|  | 491.12 | 1 |  | 491.13 | 491.15 |  | +2Na^+^ |
| 1-105 | 11473.71 | 7 |  | 1639.79^d)^ | 1639.37^d)^ |  |  |
|  | 11473.71 | 6 |  | 1913.09^d)^ | 1913.09^d)^ |  |  |
|  | 11473.71 | 5 |  | 2295.49^d)^ | 2295.49^d)^ |  |  |
|  | 11473.71 | 4 |  | 2869.12^d)^ | 2869.12^d)^ |  |  |
|  | 11456.68 | 6 |  | 1909.91^d)^ | 1909.91^d)^ |  | pyroQ |
| 106-108 | 349.18 | 1 |  | 349.15 | 349.16 |  |  |
|  | 371.18 | 1 |  | 371.13 | 371.14 |  | +Na^+^ |
| 109-144 | 4039.74 | 4 |  | 1010.58 | 1010.62 |  |  |
|  | 4039.74 | 3 |  | 1347.11 | 1347.16 |  |  |
|  | 4039.74 | 2 |  | 2020.16 | 2020.16 |  |  |
|  | 4061.74 | 4 |  | 1016.01 | 1016.11 |  | +Na^+^ |
|  | 4061.74 | 3 |  | 1354.43 | 1354.48 |  | +Na^+^ |
|  | 4061.74 | 2 |  | 2031.15 | 2031.23 |  | +Na^+^ |
|  | 4077.74 | 4 |  | 1020.06 | 1020.10 |  | +K^+^ |
|  | 4077.74 | 3 |  | 1359.76 | 1359.81 |  | +K^+^ |
|  | 4077.74 | 2 |  | 2039.13 | 2039.13 |  | +K^+^ |
|  | 4083.74 | 4 |  | 1021.57 | 1021.61 |  | +2Na^+^ |
|  | 4083.74 | 3 |  | 1361.75 | 1361.80 |  | +2Na^+^ |
|  | 4083.74 | 2 |  | 2041.65 | 2042.16 |  | +2Na^+^ |
| (1-144 minus 106-108) | 15515.85 | 8 |  | 1940.10^d)^ | 1940.10^d)^ |  |  |
|  | 15515.85 | 7 |  | 2217.10^d)^ | 2217.10^d)^ |  |  |
|  | 15515.85 | 6 |  | 2586.45^d)^ | 2586.45^d)^ |  |  |
|  | 15537.85 | 8 |  | 1942.85^d)^ | 1942.85^d)^ |  | +Na^+^ |
|  | 15537.85 | 7 |  | 2220.24^d)^ | 2220.24^d)^ |  | +Na^+^ |
|  | 15537.85 | 6 |  | 2589.95^d)^ | 2589.95^d)^ |  | +Na^+^ |
|  | 15498.82 | 7 |  | 2214.54 | 2214.54^d)^ |  | pyroQ |
|  | 15498.82 | 6 |  | 2583.55^d)^ | 2583.63^d)^ |  | pyroQ |

**Table S6.** Peptide masses of sdAbCSP1 and sdAbCSP1 – PfCSP-Cext complex after 72h tryptic digestion recorded by offline nanoESI-MS^E^ analysis (continued).

| **sequence range** | **M+H^+^** | **z** |  | ***m/z* (exp.)** | |  | **comment** |
| --- | --- | --- | --- | --- | --- | --- | --- |
| **PfCSP-Cext** | **(calcd)** | **(exp.)** |  | **SdAbCSP1** | **complex** |  |  |
| 1-19 | 2123.91 | 3 |  | / | 708.60 |  | 1 deamid |
|  | 2123.91 | 2 |  | / | 1062.39 |  | 1 deamid |
|  | 2145.91 | 3 |  | / | 715.93 |  | 1 deamid, +Na^+^ |
|  | 2145.91 | 2 |  | / | 1073.38 |  | 1 deamid, +Na^+^ |
|  | 2161.91 | 3 |  | / | 721.25 |  | 1 deamid, +K^+^ |
| 3-19 | 1863.79 | 2 |  | / | 932.33 |  |  |
|  | 1885.79 | 3 |  | / | 629.22 |  | +Na^+^ |
|  | 1885.79 | 2 |  | / | 943.33 |  | +Na^+^ |
|  | 1901.79 | 3 |  | / | 634.54 |  | +K^+^ |
|  | 1901.79 | 2 |  | / | 951.31 |  | +K^+^ |
|  | 1907.79 | 3 |  | / | 636.54 |  | +2Na^+^ |
|  | 1907.79 | 2 |  | / | 954.31 |  | +2Na^+^ |
|  | 1864.79 | 3 |  | / | 622.22 |  | 1 deamid |
|  | 1864.79 | 2 |  | / | 932.82 |  | 1 deamid |
|  | 1864.79 | 1 |  | / | 1864.65 |  | 1 deamid |
|  | 1886.77 | 3 |  | / | 629.55 |  | 1 deamid, +Na^+^ |
|  | 1886.77 | 2 |  | / | 943.82 |  | 1 deamid, +Na^+^ |
|  | 1902.77 | 3 |  | / | 634.87 |  | 1 deamid, +K^+^ |
|  | 1902.77 | 2 |  | / | 951.81 |  | 1 deamid, +K^+^ |
|  | 1908.77 | 3 |  | / | 636.87 |  | 1 deamid, +2Na^+^ |
|  | 1908.77 | 2 |  | / | 954.81 |  | 1 deamid, +2Na^+^ |
| 20-32 | 1345.63 | 2 |  | / | 673.27 |  |  |
|  | 1345.63 | 1 |  | / | 1345.55 |  |  |
|  | 1367.63 | 2 |  | / | 684.26 |  | +Na^+^ |
|  | 1367.63 | 1 |  | / | 1367.50 |  | +Na^+^ |
|  | 1383.63 | 2 |  | / | 692.24 |  | +K^+^ |
|  | 1389.63 | 2 |  | / | 695.25 |  | +2Na^+^ |
| 33-42 | 1160.48 | 2 |  | / | 580.71 |  |  |
|  | 1160.48 | 1 |  | / | 1160.41 |  |  |
| 74-94 | 2345.20 | 2 |  | / | 1173.02 |  |  |
| 20-127 | 12104.13 | 7 |  | / | 1729.99^d)^ |  |  |
|  | 12104.13 | 6 |  | / | 2018.15^d)^ |  |  |
|  | 12126.13 | 7 |  | / | 1733.13^d)^ |  | +Na^+^ |
| 33-127 | 10776.77 | 9 |  | / | 1198.27^d)^ |  |  |
|  | 10776.77 | 7 |  | / | 1540.35^d)^ |  |  |
|  | 10776.77 | 6 |  | / | 1796.91^d)^ |  |  |
|  | 10776.77 | 5 |  | / | 2156.07^d)^ |  |  |
|  | 10798.77 | 7 |  | / | 1543.35^d)^ |  | +Na^+^ |
|  | 10798.77 | 6 |  | / | 1800.40^d)^ |  | +Na^+^ |
|  | 10798.77 | 5 |  | / | 2160.28^d)^ |  | +Na^+^ |
|  | 10814.77 | 7 |  | / | / |  | +K^+^ |
|  | 10814.77 | 6 |  | / | / |  | +K^+^ |

**Table S6.** Peptide masses of sdAbCSP1 and sdAbCSP1 – PfCSP-Cext complex after 72h tryptic digestion recorded by offline nanoESI-MS^E^ analysis (continued).

| **sequence range** | **M+H^+^** | **z** |  | ***m/z* (exp.)** | |  | **comment** |
| --- | --- | --- | --- | --- | --- | --- | --- |
| **complex** | **(calcd)** | **(exp.)** |  | **sdAbCSP1** | **complex** |  |  |
| (1-144) + (1-127 minus 1-19) ^b)^ | 27913.60 | 11 |  | / | 2538.34^d)^ |  |  |
|  | 27913.60 | 10 |  | / | 2792.01^d)^ |  |  |
|  | 27913.60 | 9 |  | / | 3102.24^d)^ |  |  |
|  | 27935.60 | 11 |  | / | 2540.14^d)^ |  | +Na^+^ |
|  | 27935.60 | 10 |  | / | 2794.09^d)^ |  | +Na^+^ |
|  | 27935.60 | 9 |  | / | 3104.31^d)^ |  | +Na^+^ |
|  | 27951.60 | 10 |  | / | 2796.07^d)^ |  | +K^+^ |
| (1-144) + (1-127 minus 1-32) ^c)^ | 26584.99 | 11 |  | / | 2417.69^d)^ |  |  |
|  | 26584.99 | 10 |  | / | 2659.36^d)^ |  |  |
|  | 26584.99 | 9 |  | / | 2954.74^d)^ |  |  |
|  | 26584.99 | 8 |  | / | 3323.93^d)^ |  |  |
|  | 26606.99 | 11 |  | / | 2419.51^d)^ |  | +Na^+^ |
|  | 26606.99 | 10 |  | / | 2661.27^d)^ |  | +Na^+^ |
|  | 26606.99 | 9 |  | / | 2956.85^d)^ |  | +Na^+^ |
|  | 26606.99 | 8 |  | / | 3326.17^d)^ |  | +Na^+^ |
|  | 26622.99 | 11 |  | / | 2421.18^d)^ |  | +K^+^ |
|  | 26622.99 | 10 |  | / | 2663.38^d)^ |  | +K^+^ |
|  | 26622.99 | 9 |  | / | 2959.08^d)^ |  | +K^+^ |
|  | 26622.99 | 8 |  | / | 3328.78^d)^ |  | +K^+^ |

1. C2
2. C3
3. average mass

**Table S7.** Peptide masses of sdAbCSP1 and sdAbCSP1 – PfCSP-Cext complex after 2h tryptic digestion recorded by offline nanoESI-MS^E^ analysis.

| **sequence range** | **M+H^+^** | **z** |  | ***m/z* (exp.)** | |  | **comment** |
| --- | --- | --- | --- | --- | --- | --- | --- |
| **sdAbCSP1** | **(calcd)** | **(exp.)** |  | **sdAbCSP1** | **complex** |  |  |
| 1-19 | 1883.99 | 2 |  | / | 942.43 |  |  |
|  | 1866.97 | 1 |  | 1866.83 | 1866.85 |  | pyroQ |
|  | 1866.97 | 2 |  | / | 933.92 |  | pyroQ |
|  | 1888.97 | 2 |  | 944.92 | 944.91 |  | pyroQ, +Na^+^ |
|  | 1904.97 | 2 |  | / | 952.90 |  | pyroQ, +K^+^ |
| 39-43 | 513.34 | 1 |  | / | 513.30 |  |  |
| 39-45 | 798.48 | 1 |  | 798.43 | 798.43 |  |  |
| 39-50 | 1400.80 | 2 |  | / | 700.86 |  |  |
| 46-50 | 621.34 | 1 |  | 621.30 | 621.30 |  |  |
| 51-65 | 1576.72 | 1 |  | 1576.62 | 1576.62 |  |  |
|  | 1576.72 | 2 |  | / | 788.81 |  |  |
|  | 1598.72 | 1 |  | 1598.60 | 1598.60 |  | +Na^+^ |
| 66-72 | 836.47 | 2 |  | / | 418.71 |  |  |
|  | 858.47 | 2 |  | / | 429.70 |  | +Na^+^ |
| 73-76 | 447.12 | 1 |  | 447.20 | 447.19 |  |  |
| 1-105 | 11473.71 | 7 |  | 1639.84^d)^ | 1639.84^d)^ |  |  |
|  |  | 6 |  | 1913.00^d)^ | 1912.98^d)^ |  |  |
|  |  | 5 |  | 2295.39^d)^ | 2295.57^d)^ |  |  |
|  |  | 4 |  | 2869.45^d)^ | / |  |  |
|  | 11456.68 | 7 |  | 1637.28^d)^ | 1637.41^d)^ |  | pyroQ |
|  |  | 6 |  | 1910.16^d)^ | 1909.97^d)^ |  | pyroQ |
|  |  | 5 |  | 2291.96^d)^ | 2291.75^d)^ |  | pyroQ |
|  |  | 4 |  | 2864.71^d)^ | / |  | pyroQ |
| **106-108** | **349.18** | **1** |  | **349.16** | **349.16** |  |  |
| 1-144 | 15827.17 | 8 |  | 1979.44^d)^ | 1979.18^d)^ |  |  |
|  |  | 7 |  | 2262.06^d)^ | 2261.79^d)^ |  |  |
|  |  | 6 |  | 2638.75^d)^ | 2638.73^d)^ |  |  |
|  | 15849.17 | 8 |  | 1982.05^d)^ | 1981.55^d)^ |  | +Na^+^ |
|  |  | 7 |  | 2265.36^d)^ | 2264.63^d)^ |  | +Na^+^ |
|  | 15810.15 | 8 |  | 1977.05^d)^ | 1976.93^d)^ |  | pyroQ |
|  |  | 7 |  | 2259.35^d)^ | 2259.33^d)^ |  | pyroQ |
|  |  | 6 |  | 2635.57^d)^ | 2635.57^d)^ |  | pyroQ |
| **106-144** | **4369.91** | **3** |  | **1457.22** | **/** |  |  |
|  |  | **2** |  | **2185.35** | **/** |  |  |
| 109-144 | 4039.74 | 4 |  | / | 1010.62 |  |  |
|  |  | 3 |  | 1347.16 | 1347.16 |  |  |
|  |  | 2 |  | 2020.24 | 2020.22 |  |  |
|  | 4061.74 | 4 |  | / | 1016.11 |  | +Na^+^ |
|  |  | 3 |  | 1354.50 | 1354.48 |  | +Na^+^ |
|  |  | 2 |  | 2031.23 | 2031.23 |  | +Na^+^ |
|  | 4077.74 | 4 |  | / | 1020.10 |  | +K^+^ |
|  |  | 3 |  | / | 1359.81 |  | +K^+^ |
|  | 4083.74 | 4 |  | / | 1021.61 |  | +2Na^+^ |
|  |  | 3 |  | 1361.82 | 1361.80 |  | +2Na^+^ |
| 1-144 minus106-108 | 15515.85 | 8 |  | / | 1940.16^d)^ |  |  |
|  |  | 7 |  | / | 2217.33^d)^ |  |  |
|  |  | 6 |  | 2586.54^d)^ | 2586.87^d)^ |  |  |
|  |  | 5 |  | 3104.28^d)^ | / |  |  |
|  | 15537.85 | 8 |  | / | 1942.79^d)^ |  | +Na^+^ |
|  |  | 7 |  | / | 2220.33^d)^ |  | +Na^+^ |
|  |  | 6 |  | 2590.22^d)^ | 2590.20^d)^ |  | +Na^+^ |
|  | 15498.82 | 8 |  | / | 1937.91^d)^ |  | pyroQ |
|  |  | 7 |  | / | 2214.62^d)^ |  | pyroQ |
|  |  | 6 |  | 2583.55^d)^ | 2583.55^d)^ |  | pyroQ |
|  |  | 5 |  | 3100.13^d)^ | / |  | pyroQ |

**Table S7.** Peptide masses of sdAbCSP1 and sdAbCSP1 – PfCSP-Cext complex after 2h tryptic digestion recorded by offline nanoESI-MS^E^ analysis (continued).

| **sequence range** | **M+H^+^** | **z** |  | ***m/z* (exp.)** | |  | **comment** |
| --- | --- | --- | --- | --- | --- | --- | --- |
| **PfCSP Cext** | **(calcd)** | **(exp.)** |  | **sdAbCSP1** | **complex** |  |  |
| 1-19 | 2122.93 | 3 |  | / | 708.26 |  |  |
|  |  | 2 |  | / | 1061.89 |  |  |
|  | 2144.93 | 3 |  | / | 715.59 |  | +Na+ |
|  | 2123.91 | 3 |  | / | 708.59 |  | 1 deamid |
|  |  | 2 |  | / | 1062.39 |  | 1 deamid |
|  | 2145.91 | 3 |  | / | 715.91 |  | 1 deamid, +Na+ |
|  |  | 2 |  | / | 1073.37 |  | 1 deamid, +Na+ |
|  | 2161.91 | 3 |  | / | 721.24 |  | 1 deamid, +K+ |
| 3-19 | 1863.79 | 3 |  | / | 621.90 |  |  |
|  |  | 2 |  | / | 932.33 |  |  |
|  | 1885.79 | 2 |  | / | 943.32 |  | +Na+ |
|  | 1864.77 | 3 |  | / | 622.20 |  | 1 deamid |
|  |  | 2 |  | / | 932.82 |  | 1 deamid |
|  | 1886.77 | 2 |  | / | 943.81 |  | 1 deamid, +Na+ |
|  | 1902.77 | 2 |  | / | 951.81 |  | 1 deamid, +K+ |
| 1-32 | 3449.54 | 4 |  | / | 863.08 |  |  |
|  |  | 3 |  | / | 1150.44 |  |  |
|  | 3450.53 | 4 |  | / | 863.32 |  | 1 deamid |
|  |  | 3 |  | / | 1150.77 |  | 1 deamid |
|  |  | 2 |  | / | 1725.64 |  | 1 deamid |
|  | 3472.53 | 4 |  | / | 868.82 |  | 1 deamid, +Na+ |
|  |  | 3 |  | / | 1158.10 |  | 1 deamid, +Na+ |
|  | 3488.53 | 4 |  | / | 872.81 |  | 1 deamid, +K+ |
|  |  | 3 |  | / | 1163.41 |  | 1 deamid, +K+ |
|  | 3494.53 | 3 |  | / | 1165.42 |  | 1 deamid, +2Na+ |
| 3-32 | 3191.39 | 3 |  | / | 1064.39 |  | 1 deamid |
|  | 3213.39 | 3 |  | / | 1071.71 |  | 1 deamid, +Na+ |
| 20-32 | 1345.63 | 2 |  | / | 673.27 |  |  |
|  | 1367.63 | 2 |  | / | 684.26 |  | +Na+ |
|  | 1383.63 | 2 |  | / | 692.24 |  | +K+ |
|  | 1389.63 | 2 |  | / | 695.25 |  | +2Na+ |
| 20-127 | 12104.13 | 8 |  | / | 1513.86^d)^ |  |  |
|  |  | 7 |  | / | 1729.97^d)^ |  |  |
|  |  | 6 |  | / | 2018.15^d)^ |  |  |
|  | 12126.13 | 7 |  | / | 1732.98^d)^ |  | +Na+ |
| 33-127 | 10776.77 | 9 |  | / | 1198.15^d)^ |  |  |
|  |  | 7 |  | / | 1540.20^d)^ |  |  |
|  |  | 6 |  | / | 1796.72^d)^ |  |  |
|  |  | 5 |  | / | 2156.05^d)^ |  |  |
|  | 10798.77 | 7 |  | / | 1543.33^d)^ |  | +Na+ |
|  |  | 6 |  | / | 1800.38^d)^ |  | +Na+ |
|  |  | 5 |  | / | 2160.47^d)^ |  | +Na+ |
| 1-127 | 14209.36 | 8 |  | / | 1777.07d) |  |  |
|  |  | 7 |  | / | 2030.79d) |  |  |
|  |  | 6 |  | / | 2369.21d) |  |  |
|  | 14231.36 | 8 |  | / | 1779.70d) |  | +Na+ |

**Table S7.** Peptide masses of sdAbCSP1 and sdAbCSP1 – PfCSP-Cext complex after 2h tryptic digestion recorded by offline nanoESI-MS^E^ analysis (continued).

| **sequence range** | **M+H^+^** | **z** |  | ***m/z* (exp.)** | |  | **comment** |
| --- | --- | --- | --- | --- | --- | --- | --- |
| **complex** | **(calcd)** | **(exp.)** |  | **sdAbCSP1** | **complex** |  |  |
| (1-144) + (1-127) ^a)^ | 30035.53 | 10 |  | / | 3004.55^d)^ |  |  |
|  | 30057.53 | 10 |  | / | 3006.70^d)^ |  | +Na^+^ |
|  | 30018.50 | 12 |  | / | 2502.26^d)^ |  | pyroQ |
|  |  | 11 |  | / | 2729.73^d)^ |  | pyroQ |
|  |  | 10 |  | / | 3002.71^d)^ |  | pyroQ |
| (1-144) + (1-127 minus 1-19) ^b)^ | 27913.60 | 11 |  | / | 2538.21^d)^ |  |  |
|  |  | 10 |  | / | 2792.20^d)^ |  |  |
|  |  | 9 |  | / | 3102.22^d)^ |  |  |
|  | 27935.60 | 11 |  | / | 2540.29^d)^ |  | +Na^+^ |
|  |  | 10 |  | / | 2794.18^d)^ |  | +Na^+^ |
|  |  | 9 |  | / | 3104.52^d)^ |  | +Na^+^ |
|  | 27951.60 | 10 |  | / | 2795.95^d)^ |  | +K^+^ |
| (1-144) +(1-127 minus 1-32) ^c)^ | 26584.99 | 11 |  | / | 2417.54^d)^ |  |  |
|  |  | 10 |  | / | 2659.27^d)^ |  |  |
|  |  | 9 |  | / | 2954.62^d)^ |  |  |
|  |  | 8 |  | / | 3324.16^d)^ |  |  |
|  | 26606.99 | 11 |  | / | 2419.43^d)^ |  | +Na^+^ |
|  |  | 10 |  | / | 2661.43^d)^ |  | +Na^+^ |
|  |  | 9 |  | / | 2957.04^d)^ |  | +Na^+^ |
|  | 26622.99 | 10 |  | / | 2663.38^d)^ |  | +K^+^ |
|  |  | 9 |  | / | 2959.08^d)^ |  | +K^+^ |

1. C1
2. C2
3. C3
4. average mass

**Table S8.** Effects of amino acid residues in mutated sdAbCSP1-exchanged amino acid residues on strengths of interaction with PfCSP-Cext. ^a)^

| amino acid residues in sdAbCSP1 ^b)^ | ΔΔG values in kcal / mol ^c)^ | | | | | | | | | | | | | | | | | | | |
| --- | --- | --- | --- | --- | --- | --- | --- | --- | --- | --- | --- | --- | --- | --- | --- | --- | --- | --- | --- | --- |
|  | A | C | D | E | F | G | H | I | K | L | M | N | P | Q | R | S | T | V | W | Y |
| R27 | +0.48 | +0.13 | +0.01 | +0.11 | +0.11 | +0.78 | -0.01 | +0.14 | +0.07 | +0.23 | +0.21 | -0.02 | +0.75 | +0.18 | ±0.00 | +0.15 | -0.02 | +0.12 | +0.08 | -0.03 |
| S31 | +0.52 | +0.76 | +0.94 | +0.39 | +0.81 | +0.70 | +0.21 | +0.88 | +0.21 | +0.86 | +0.48 | +0.34 | +0.61 | +0.13 | +0.06 | ±0.00 | +0.50 | +0.78 | +0.35 | +0.51 |
| Y32 | +1.48 | +0.79 | +1.49 | +1.32 | +0.17 | +1.83 | +0.66 | +0.66 | +1.11 | +0.57 | +0.90 | +1.11 | +1.79 | +1.04 | +0.98 | +1.45 | +1.32 | +0.84 | +0.30 | ±0.00 |
| N57 | +0.35 | +0.28 | +0.44 | +0.43 | +0.18 | +0.32 | 0.16 | -0.02 | +0.34 | +0.17 | +0.08 | ±0.00 | +0.06 | +0.19 | +0.19 | +0.16 | +0.11 | -0.17 | +0.38 | +0.25 |
| Y59 | +0.95 | +0.81 | +0.92 | +0.71 | +0.28 | +1.04 | +0.59 | +0.44 | +0.84 | +0.53 | +0.69 | +0.86 | +1.17 | +0.68 | +0.66 | +0.91 | +0.72 | +0.50 | +0.14 | ±0.00 |
| L100 | +2.22 | +1.03 | +2.55 | +2.71 | +0.59 | +2.40 | +1.08 | +0.52 | +2.02 | ±0.00 | +0.71 | +1.92 | +1.94 | +2.10 | +1.43 | +1.93 | +1.63 | +0.59 | +0.34 | +0.48 |
| L101 | +2.20 | +1.49 | +2.27 | +2.26 | +0.04 | +2.56 | +0.90 | +1.17 | +1.91 | ±0.00 | +0.80 | +1.44 | +2.66 | +1.49 | +1.23 | +2.07 | +1.46 | +1.21 | +0.04 | +0.28 |
| Q102 | +1.23 | +1.35 | +0.94 | +0.96 | -0.09 | +1.40 | +0.37 | +0.49 | +0.97 | +0.12 | +0.12 | +0.64 | +0.61 | ±0.00 | +0.34 | +0.87 | +0.65 | +0.55 | -0.22 | +0.17 |
| F103 | +5.17 | +3.32 | +4.93 | +4.95 | ±0.00 | +4.86 | +2.99 | +2.24 | +4.61 | +2.43 | +2.76 | +4.37 | +5.21 | +4.37 | +3.57 | +4.44 | +3.94 | +3.17 | +1.07 | +1.28 |
| G104 | +0.85 | +0.47 | +0.91 | +1.05 | -0.15 | ±0.00 | -0.10 | +0.26 | +0.31 | +0.40 | +0.37 | +0.53 | +0.42 | +0.48 | -0.19 | +0.53 | +0.34 | +0.45 | -0.53 | -0.42 |
| R105 | +0.91 | +0.76 | +0.65 | +0.98 | +0.33 | +0.87 | +0.22 | +0.26 | +0.60 | +0.26 | +0.34 | +0.47 | +0.31 | +0.59 | ±0.00 | +0.77 | +0.63 | +0.34 | +0.37 | +0.46 |
| R108 | +0.42 | +0.25 | -0.04 | +0.29 | +0.16 | +0.36 | +0.07 | +0.21 | +0.14 | +0.15 | +0.14 | +0.02 | +0.40 | +0.30 | ±0.00 | +0.19 | +0.06 | +0.26 | +0.14 | +0.24 |
| A110 | ±0.00 | -0.05 | -0.19 | -0.06 | +0.15 | -0.03 | -0.05 | +0.14 | +0.03 | +0.15 | +0.18 | -0.12 | +0.15 | +0.03 | -0.02 | -0.20 | -0.06 | +0.12 | -0.01 | +0.08 |
| D111 | +0.94 | +0.42 | ±0.00 | +0.47 | +0.03 | +0.63 | +0.41 | +0.35 | +0.55 | +0.27 | +0.36 | +0.51 | +0.23 | +0.51 | +0.56 | +0.62 | +0.46 | +0.35 | +0.32 | +0.18 |
| Y112 | +1.44 | +1.15 | +1.24 | +1.20 | +0.41 | +1.68 | +0.73 | +0.82 | +1.18 | +0.78 | +0.82 | +1.15 | +1.12 | +1.08 | +0.92 | +1.17 | +1.00 | +1.03 | +0.30 | ±0.00 |
| D113 | +0.63 | +0.82 | ±0.00 | +0.47 | +0.28 | +0.84 | +0.24 | +0.27 | +0.46 | +0.31 | +0.41 | +0.53 | +0.39 | +0.33 | +0.34 | +0.68 | +0.34 | +0.14 | -0.08 | +0.28 |
| Y114 | +2.34 | +1.83 | +2.59 | +2.67 | +0.78 | +2.53 | +1.43 | +1.44 | +2.13 | +1.47 | +1.09 | +2.11 | +2.80 | +2.19 | +1.66 | +2.49 | +2.12 | +1.72 | +0.69 | ±0.00 |

a) no cumulative effects; free energy difference calculations for individual amino acid positions.

b) residues with a shortest atom distance below 4 Å between PfCSP-Cext and sdAbCSP1.

c) orange: max. value; yellow: min. value; gray: no exchange.

**Table S9.** Effects of amino acid residues in mutated PfCSP-Cext-exchanged amino acid residues on strengths of interaction with sdAbCSP1. ^a)^

| amino acid residues in PfCSP-Cext ^b)^ | ΔΔG values in kcal / mol ^c)^ | | | | | | | | | | | | | | | | | | | |
| --- | --- | --- | --- | --- | --- | --- | --- | --- | --- | --- | --- | --- | --- | --- | --- | --- | --- | --- | --- | --- |
|  | A | C | D | E | F | G | H | I | K | L | M | N | P | Q | R | S | T | V | W | Y |
| S29 | +0.61 | +0.37 | +0.65 | +0.77 | +0.72 | +0.46 | +0.61 | +0.64 | +0.82 | +0.60 | +0.71 | +0.56 | +0.01 | +0.67 | +0.75 | ±0.00 | +0.41 | +0.44 | +0.49 | +0.68 |
| K42 | +0.95 | +0.90 | +0.94 | +0.55 | +0.14 | +1.07 | +0.29 | +0.42 | ±0.00 | +0.32 | +0.32 | +0.83 | +1.02 | +0.41 | +0.27 | +0.83 | +0.61 | +0.60 | +0.09 | +0.27 |
| K45 | +1.64 | +1.41 | +1.87 | +1.30 | +0.37 | +1.74 | +1.04 | +0.49 | ±0.00 | +0.41 | +0.43 | +1.45 | +1.37 | +0.72 | +0.46 | +1.65 | +1.41 | +1.11 | +0.37 | +0.75 |
| E46 | +1.26 | +1.62 | +0.69 | ±0.00 | +0.88 | +1.12 | 0.31 | +1.51 | +0.47 | +1.15 | +0.88 | +0.51 | +1.30 | +0.24 | +0.28 | +0.91 | +0.95 | +1.80 | +0.36 | +0.53 |
| L48 | +1.54 | +1.23 | +2.32 | +1.71 | +0.89 | +2.24 | +1.53 | +0.41 | +1.69 | ±0.00 | +0.98 | +1.93 | +2.72 | +1.63 | +1.32 | +2.10 | +1.57 | +0.60 | +0.84 | +0.93 |
| N49 | +1.38 | +1.02 | +1.40 | +1.22 | +0.44 | +2.31 | +0.62 | +1.02 | +0.93 | +0.52 | +0.38 | ±0.00 | +1.60 | +1.04 | +0.80 | +1.16 | +0.88 | +1.01 | -0.00 | +0.43 |
| Q52 | +1.17 | +0.65 | +1.51 | +1.42 | -0.30 | +1.68 | +0.72 | -0.01 | +0.94 | -0.20 | +0.02 | +1.10 | +1.20 | ±0.00 | +0.60 | +1.31 | +1.23 | +0.12 | -0.22 | -0.28 |
| L55 | +1.09 | +0.94 | +1.31 | +1.32 | +0.27 | +1.35 | +0.82 | +0.67 | +1.21 | ±0.00 | +0.44 | +1.28 | +1.46 | +1.05 | +0.97 | +1.36 | +1.04 | +0.78 | +0.48 | +0.55 |
| K83 | +0.29 | +0.15 | +0.07 | +0.42 | -0.17 | +0.37 | -0.17 | -0.19 | ±0.00 | -0.26 | +0.03 | -0.07 | -0.23 | +0.14 | +0.10 | +0.01 | +0.01 | -0.21 | -0.03 | +0.08 |
| D84 | +1.48 | +0.75 | ±0.00 | +1.09 | +0.03 | +1.25 | +0.12 | +0.74 | +1.38 | +0.59 | +0.60 | +0.39 | +0.88 | +1.06 | +0.68 | +0.53 | +0.38 | +1.04 | -0.07 | -0.17 |
| E85 | +0.72 | +0.37 | +0.15 | ±0.00 | -0.01 | +0.43 | -0.10 | +0.19 | +0.30 | +0.05 | +0.10 | +0.19 | +0.50 | +0.20 | +0.08 | +0.33 | +0.08 | +0.38 | +0.10 | +0.21 |
| L86 | +2.19 | +1.30 | +2.07 | +1.86 | +0.63 | +2.56 | +0.93 | +0.90 | +1.67 | ±0.00 | +0.67 | +1.89 | +2.62 | +1.52 | +1.29 | +1.94 | +1.60 | +0.80 | +0.70 | +0.72 |
| D87 | +2.12 | +1.23 | ±0.00 | +1.36 | +0.99 | +1.71 | +0.78 | +1.23 | +1.36 | +1.22 | +1.00 | +0.75 | +0.17 | +1.30 | +1.21 | +1.20 | +1.07 | +1.16 | +0.84 | +0.93 |
| Y88 | +1.83 | +1.20 | +1.62 | +1.51 | +0.32 | +2.11 | +1.00 | +0.81 | +1.31 | +0.83 | +1.05 | +1.45 | +1.15 | +1.23 | +1.11 | +1.63 | +1.35 | +1.09 | +0.24 | ±0.00 |
| N90 | +0.06 | -0.00 | -0.08 | -0.11 | +0.16 | +0.21 | -0.03 | +0.07 | -0.13 | +0.08 | +0.06 | ±0.00 | -0.08 | -0.08 | -0.06 | -0.03 | -0.04 | +0.01 | +0.14 | +0.10 |

a) no cumulative effects; free energy difference calculations for individual amino acid positions.

b) residues with a shortest atom distance below 4 Å between PfCSP-Cext and sdAbCSP1.

c) orange: max. value; yellow: min. value; gray: no exchange.

**Table S10.** Buried surface area of the paratope of sdAbCSP1 in contact with the epitope of PfCSP-Cext.

| **Amino acid Residue** | **ASA Value of each amino acid of the sdAbCSP1 – PfCSP-Cext complex [Å^2^]** | **ASA Value of each amino acid of the sdAbCSP1 alone [Å^2^]** | **Difference of ASA values between sdAbCSP1 – PfCSP-Cext complex and sdAbCSP1 alone [Å^2^]** |
| --- | --- | --- | --- |
| R27 | 146.20 | 196.40 | 50.20 |
| S31 | 35.23 | 53.88 | 18.65 |
| Y32 | 19.27 | 38.13 | 18.86 |
| N57 | 66.92 | 81.65 | 14.73 |
| Y59 | 64.06 | 65.56 | 1.50 |
| L100 | 0.13 | 70.15 | 70.02 |
| L101 | 4.53 | 70.07 | 65.54 |
| Q102 | 37.01 | 96.42 | 59.41 |
| F103 | 4.36 | 154.05 | 149.69 |
| G104 | 8.20 | 50.66 | 42.46 |
| R105 | 122.79 | 197.88 | 75.09 |
| R108 | 103.46 | 154.65 | 51.19 |
| A110 | 80.86 | 90.01 | 9.15 |
| D111 | 15.26 | 32.78 | 17.52 |
| Y112 | 8.22 | 8.22 | 0.00 |
| D113 | 53.94 | 101.01 | 47.07 |
| Y114 | 22.30 | 69.03 | 46.73 |

**Table S11.** Buried surface area of the epitope of PfCSP-Cext in contact with the paratope of sdAbCSP1.

| **Amino acid Residue** | **ASA Value of each amino acid of the sdAbCSP1 – PfCSP-Cext complex [Å^2^]** | **ASA Value of each amino acid of the PfCSP-Cext alone [Å^2^]** | **Difference of ASA values between sdAbCSP1 – PfCSP-Cext complex and PfCSP Cext alone [Å^2^]** |
| --- | --- | --- | --- |
| S29 | 56.57 | 95.49 | 38.92 |
| K42 | 89.72 | 137.23 | 47.51 |
| K45 | 9.2 | 123.28 | 114.08 |
| E46 | 54.31 | 99.21 | 44.90 |
| L48 | 0.00 | 23.75 | 23.75 |
| N49 | 15.49 | 110.18 | 94.69 |
| Q52 | 38.97 | 96.60 | 57.63 |
| L55 | 6.17 | 17.62 | 11.45 |
| K83 | 95.85 | 125.75 | 29.90 |
| D84 | 24.38 | 135.07 | 110.69 |
| E85 | 63.37 | 108.57 | 45.20 |
| L86 | 0.75 | 30.04 | 29.29 |
| D87 | 20.11 | 61.85 | 41.74 |
| Y88 | 17.66 | 60.47 | 42.81 |
| N90 | 109.25 | 114.92 | 5.67 |

**Table S12.** Molecular information of PfCSP-Cext (antigen), sdAbCSP1 (nanobody), and sdAbCSP1 – PfCSP-Cext complex.

| protein/complex ^a)^ | number of atoms | mean charge | MM (calcd) ^b)^ [Da] | MM (exp.) [Da] |
| --- | --- | --- | --- | --- |
| sdAbCSP1 | 2157 | 6.89 | 15826.17 | 15826.31 ± 0.01 |
| pQ-sdAbCSP1 | 2139 | 6.87 | 15809.14 | 15808.25 ± 0.06 |
| PfCSP-Cext | 1922 | 7.03 | 14208.36 | 14209.01 ± 0.52 |
| sdAbCSP1 - PfCSP-Cext | 4079 | 9.90 | 30034.53 | 30035.51 ± 0.45 |
| pQ-sdAbCSP1 - PfCSP-Cext | 4061 | 9.90 | 30017.50 | 30018.25 ± 0.58 |

^a)^ pQ: N-terminal pyroglutamine residue ^b)^ average masses

**Table S13.** Ion species, *m/z* values, charge states and intensities for sdAbCSP1 complexed with PfCSP-Cext at various collision cell voltage differences.

measurement 1

| ∆CV | sdAbCSP1 *m/z* and charge state ^a)^ | | | | | | | |  | pyroQ sdAbCSP1 *m/z* and charge state ^a)^ | | | | | | | |
| --- | --- | --- | --- | --- | --- | --- | --- | --- | --- | --- | --- | --- | --- | --- | --- | --- | --- |
|  | 1583.62 | 1759.46 | 1979.18 | 2261.84 | 2638.70 | 3166.08 | 3957.43 ^b)^ | 5276.39 |  | 1581.91 | 1757.57 | 1977.10 | 2259.39 | 2635.79 | 3162.74 | 3952.85 ^b)^ | 5270.71 |
|  | 10+ | 9+ | 8+ | 7+ | 6+ | 5+ | 4+ | 3+ |  | 10+ | 9+ | 8+ | 7+ | 6+ | 5+ | 4+ | 3+ |
| 2 | n.d. | n.d. | n.d. | n.d. | n.d. | n.d. | n.d. | n.d. |  | n.d. | n.d. | n.d. | n.d. | n.d. | n.d. | n.d. | n.d. |
| 5 | n.d. | n.d. | n.d. | n.d. | n.d. | n.d. | n.d. | n.d. |  | n.d. | n.d. | n.d. | n.d. | n.d. | n.d. | n.d. | n.d. |
| 10 | n.d. | n.d. | n.d. | n.d. | n.d. | n.d. | n.d. | n.d. |  | n.d. | n.d. | n.d. | n.d. | n.d. | n.d. | n.d. | n.d. |
| 15 | n.d. | n.d. | n.d. | n.d. | n.d. | n.d. | n.d. | n.d. |  | n.d. | n.d. | n.d. | n.d. | n.d. | n.d. | n.d. | n.d. |
| 20 | n.d. | n.d. | n.d. | n.d. | n.d. | n.d. | n.d. | n.d. |  | n.d. | n.d. | n.d. | n.d. | n.d. | n.d. | n.d. | n.d. |
| 25 | n.d. | n.d. | n.d. | n.d. | n.d. | n.d. | n.d. | n.d. |  | n.d. | n.d. | n.d. | n.d. | n.d. | n.d. | n.d. | n.d. |
| 30 | n.d. | n.d. | n.d. | n.d. | n.d. | n.d. | 2440 | n.d. |  | n.d. | n.d. | n.d. | n.d. | n.d. | n.d. | 1290 | n.d. |
| 35 | n.d. | n.d. | n.d. | n.d. | n.d. | n.d. | 12950 | n.d. |  | n.d. | n.d. | 200 | 21560 | 33550 | 12360 | 4421 | n.d. |
| 40 | n.d. | n.d. | 200 | 42950 | 68570 | 28900 | 17630 | n.d. |  | n.d. | n.d. | 200 | 71460 | 137600 | 44560 | 26250 | n.d. |
| 45 | n.d. | n.d. | 200 | 32610 | 62000 | 24460 | 50090 | n.d. |  | n.d. | n.d. | 200 | 53540 | 113200 | 36200 | 32180 | n.d. |
| 50 | n.d. | n.d. | 200 | 55110 | 118500 | 48030 | 54770 | n.d. |  | n.d. | n.d. | 200 | 102300 | 224000 | 74250 | 92470 | n.d. |
| 55 | n.d. | n.d. | 2416 | 48620 | 109100 | 44360 | 64640 | n.d. |  | n.d. | n.d. | 2701 | 92000 | 209400 | 70160 | 100400 | n.d. |
| 60 | n.d. | n.d. | 3050 | 50280 | 115700 | 48150 | 63850 | n.d. |  | n.d. | n.d. | 3810 | 101100 | 225800 | 81780 | 121500 | n.d. |
| 65 | n.d. | n.d. | 4160 | 48690 | 107900 | 46920 | 46720 | n.d. |  | n.d. | n.d. | 5142 | 97380 | 220300 | 86640 | 126600 | n.d. |
| 70 | n.d. | n.d. | 3380 | 31400 | 68550 | 34890 | 53110 | n.d. |  | n.d. | n.d. | 4605 | 64810 | 144200 | 65800 | 97140 | n.d. |
| 75 | n.d. | n.d. | 4481 | 33470 | 66300 | 39100 | 45640 | n.d. |  | n.d. | 200 | 6465 | 71560 | 147300 | 76410 | 120400 | 200 |
| 80 | n.d. | 200 | 4135 | 26650 | 46710 | 30620 | 32940 | 200 |  | n.d. | 200 | 6434 | 59640 | 110400 | 62770 | 108900 | 200 |
| 85 | n.d. | 200 | 3940 | 21910 | n.d. | 20420 | 20940 | 200 |  | n.d. | 200 | 5809 | 49560 | 70670 | 45300 | 84990 | 200 |
| 90 | n.d. | 200 | 3332 | 16140 | n.d. | 11860 | 10560 | 200 |  | n.d. | 200 | 5143 | 36970 | 39340 | 28840 | 56420 | 200 |
| 95 | n.d. | 200 | 3160 | 11140 | n.d. | 6553 | 5009 | 200 |  | n.d. | 200 | 3935 | 25790 | 18490 | 16310 | 30710 | 200 |
| 100 | n.d. | 200 | 2844 | 7465 | n.d. | 3611 | 2085 | 200 |  | n.d. | 200 | 2831 | 16110 | 8240 | 8396 | 14810 | 200 |
| 105 | n.d. | n.d. | n.d. | 42950 | n.d. | n.d. | n.d. | n.d. |  | n.d. | 200 | 2375 | 10740 | 4157 | 3791 | 5922 | 200 |
| 110 | n.d. | n.d. | n.d. | 32610 | n.d. | n.d. | n.d. | n.d. |  | n.d. | 200 | 2746 | 8914 | 3027 | 2414 | 2325 | 200 |
| 115 | n.d. | n.d. | n.d. | 55110 | n.d. | n.d. | 430 | n.d. |  | n.d. | 200 | 1969 | 4947 | n.d. | 1526 | 1013 | 200 |
| 120 | n.d. | n.d. | n.d. | 48620 | n.d. | n.d. | n.d. | n.d. |  | n.d. | 200 | 1618 | 3219 | 1588 | n.d. | 657 | 200 |

1. calculated *m/z* value. Imputed intensity values which are necessary for optimized Gauss fits are printed in red and are equal to the background intensity at the given *m/z*. n.d.: value not determined / ion signal not present.
2. Ion signal intensity was not included in Gauss fit since this charge state represents an individual sdAbCSP1 protein conformation.

**Table S13.** Ion species, *m/z* values, charge states and intensities for sdAbCSP1 complexed with PfCSP-Cext at various collision cell voltage differences (continued).

measurement 1

| **∆**CV | PfCSP-Cext *m/z* and charge state ^a)^ | | | | | | |
| --- | --- | --- | --- | --- | --- | --- | --- |
|  | 1579.71 | 1777.15 | 2030.89 | 2369.22 | 2842.83 | 3553.35 | 4737.12 |
|  | 9+ | 8+ | 7+ | 6+ | 5+ | 4+ | 3+ |
| 2 | n.d. | n.d. | n.d. | n.d. | 1815 | n.d. | n.d. |
| 5 | n.d. | n.d. | n.d. | n.d. | 3303 | n.d. | n.d. |
| 10 | n.d. | n.d. | n.d. | n.d. | 3953 | n.d. | n.d. |
| 15 | n.d. | n.d. | n.d. | n.d. | 2523 | n.d. | n.d. |
| 20 | n.d. | 200 | 3254 | n.d. | 9311 | 2200 | 200 |
| 25 | n.d. | 200 | n.d. | 3871 | 5300 | 1990 | 200 |
| 30 | n.d. | n.d. | n.d. | n.d. | n.d. | n.d. | n.d. |
| 35 | n.d. | n.d. | n.d. | n.d. | n.d. | n.d. | n.d. |
| 40 | n.d. | 200 | 93470 | 159400 | 45600 | n.d. | 200 |
| 45 | n.d. | 200 | 71000 | 191900 | n.d. | 31190 | 200 |
| 50 | n.d. | 2813 | 162500 | 580900 | 80330 | 64680 | n.d. |
| 55 | n.d. | 3495 | 155500 | 596500 | 72180 | 57800 | n.d. |
| 60 | n.d. | 4364 | 175100 | 674000 | 83180 | 62500 | n.d. |
| 65 | n.d. | 6340 | 166400 | 620000 | 94980 | 60070 | n.d. |
| 70 | n.d. | 4896 | 112300 | 407900 | 89530 | 41580 | n.d. |
| 75 | n.d. | 6702 | 131400 | 455200 | 146500 | 44260 | n.d. |
| 80 | n.d. | 6682 | 114900 | 384400 | 160600 | 34050 | n.d. |
| 85 | n.d. | 6798 | 89710 | 300800 | 135100 | 23620 | n.d. |
| 90 | n.d. | 6211 | 62740 | 221000 | 98310 | 14490 | n.d. |
| 95 | n.d. | 5331 | 37990 | 148000 | 56910 | 8410 | n.d. |
| 100 | n.d. | 4853 | 21350 | 96550 | 29640 | 4723 | n.d. |
| 105 | n.d. | 4058 | 11530 | 53690 | 13630 | 2660 | n.d. |
| 110 | n.d. | 5537 | 8571 | 31570 | 6234 | 1823 | n.d. |
| 115 | 200 | 3617 | 5168 | 13780 | 2826 | 1124 | n.d. |
| 120 | 200 | 4110 | 4001 | 6542 | 1766 | 777 | n.d. |

1. calculated *m/z* value. Imputed intensity values which are necessary for optimized Gauss fits are printed in red and are equal to the background intensity at the given *m/z*. n.d.: value not determined / ion signal not present.

**Table S13.** Ion species, *m/z* values, charge states and intensities for sdAbCSP1 complexed with PfCSP-Cext at various collision cell voltage differences (continued).

measurement 1

| **∆**CV | sdAbCSP1-PfCSP-Cext complex *m/z* and charge state ^a)^ | | | | | |  | pyroQ sdAbCSP1-PfCSP-Cext complex *m/z* and charge state ^a)^ | | | | | |
| --- | --- | --- | --- | --- | --- | --- | --- | --- | --- | --- | --- | --- | --- |
|  | 2503.88 | 2731.53 | 3004.66 | 3338.36 | 3755.31 | 4291.65 |  | 2502.46 | 2729.98 | 3002.82 | 3336.37 | 3753.26 | 4289.21 |
|  | 12+ | 11+ | 10+ | 9+ | 8+ | 7+ |  | 12+ | 11+ | 10+ | 9+ | 8+ | 7+ |
| 2 | 200 | 90700 | 240300 | 93820 | 2644 | 200 |  | 200 | 117600 | 309300 | 120500 | 3363 | 200 |
| 5 | 200 | 150900 | 432900 | 230000 | 12390 | 200 |  | 200 | 187000 | 543900 | 283000 | 15020 | 200 |
| 10 | 200 | 154600 | 456900 | 238800 | 13270 | 200 |  | 200 | 194900 | 574400 | 301000 | 16650 | 200 |
| 15 | 200 | 85350 | 251800 | 126800 | 6556 | 200 |  | 200 | 99540 | 295200 | 149000 | 7790 | 200 |
| 20 | 200 | 176700 | 570900 | 297900 | 14930 | 200 |  | 200 | 211300 | 708700 | 373200 | 18220 | 200 |
| 25 | 200 | 68520 | 248200 | 124100 | 6238 | 200 |  | 200 | 76160 | 285600 | 143300 | 7071 | 200 |
| 30 | 200 | 48640 | 220900 | 111300 | 5636 | 200 |  | 200 | 53850 | 251000 | 128400 | 6730 | 200 |
| 35 | 200 | 55540 | 331200 | 185700 | 10310 | 200 |  | 200 | 66940 | 398300 | 231200 | 14260 | 200 |
| 40 | 200 | 93110 | 767600 | 521500 | 31760 | 200 |  | 200 | 124000 | 968300 | 703700 | 56440 | 200 |
| 45 | 200 | 36600 | 366100 | 319400 | 21640 | 200 |  | 200 | 45370 | 429500 | 415700 | 35120 | 200 |
| 50 | 200 | 31720 | 398900 | 558000 | 37110 | 200 |  | 200 | 35920 | 516400 | 841800 | 70220 | 200 |
| 55 | 200 | 11710 | 187800 | 449300 | 33750 | 200 |  | 200 | 9432 | 249400 | 696900 | 61160 | 200 |
| 60 | 200 | 8089 | 129400 | 436700 | 35650 | 200 |  | 200 | 4775 | 178100 | 715500 | 62470 | 200 |
| 65 | 200 | 5910 | 74680 | 357600 | 34150 | 200 |  | 200 | 3590 | 101600 | 584000 | 58250 | 200 |
| 70 | 200 | 1846 | 25800 | 189300 | 21230 | 200 |  | 200 | 1529 | 33550 | 304300 | 34170 | 200 |
| 75 | 200 | 200 | 12570 | 129100 | 21630 | 200 |  | 200 | 200 | 15670 | 212500 | 34660 | 200 |
| 80 | 200 | 200 | 4327 | 47960 | 17200 | 200 |  | 200 | 1234 | 6192 | 80990 | 28000 | 200 |
| 85 | 200 | 1206 | 1769 | 14210 | 13340 | 200 |  | 200 | 1433 | 2451 | 24300 | 22440 | 200 |
| 90 | 200 | 1390 | 1102 | 4888 | 10270 | 200 |  | 200 | 200 | 1510 | 7040 | 17860 | 200 |
| 95 | n.d. | n.d. | n.d. | n.d. | n.d. | n.d. |  | 200 | 200 | 1241 | 2373 | 12430 | 200 |
| 100 | n.d. | n.d. | n.d. | n.d. | n.d. | n.d. |  | n.d. | n.d. | n.d. | n.d. | n.d. | n.d. |
| 105 | n.d. | n.d. | n.d. | 2211 | n.d. | n.d. |  | n.d. | n.d. | n.d. | n.d. | n.d. | n.d. |
| 110 | n.d. | n.d. | n.d. | 2246 | n.d. | n.d. |  | n.d. | n.d. | n.d. | n.d. | n.d. | n.d. |
| 115 | n.d. | n.d. | n.d. | n.d. | n.d. | n.d. |  | n.d. | n.d. | n.d. | n.d. | n.d. | n.d. |
| 120 | n.d. | n.d. | n.d. | n.d. | n.d. | n.d. |  | n.d. | n.d. | n.d. | n.d. | n.d. | n.d. |

1. calculated *m/z* value. Imputed intensity values which are necessary for optimized Gauss fits are printed in red and are equal to the background intensity at the given *m/z*. n.d.: value not determined / ion signal not present.

**Table S14.** Ion species, *m/z* values, charge states and intensities for sdAbCSP1 complexed with PfCSP-Cext at various collision cell voltage differences.

measurement 2

| ∆CV | sdAbCSP1 *m/z* and charge state ^a)^ | | | | | | | |  | pyroQ sdAbCSP1 *m/z* and charge state ^a)^ | | | | | | | |
| --- | --- | --- | --- | --- | --- | --- | --- | --- | --- | --- | --- | --- | --- | --- | --- | --- | --- |
|  | 1583.62 | 1759.46 | 1979.18 | 2261.84 | 2638.70 | 3166.08 | 3957.43 ^b)^ | 5276.39 |  | 1581.91 | 1757.57 | 1977.10 | 2259.39 | 2635.79 | 3162.74 | 3952.85 ^b)^ | 5270.71 |
|  | 10+ | 9+ | 8+ | 7+ | 6+ | 5+ | 4+ | 3+ |  | 10+ | 9+ | 8+ | 7+ | 6+ | 5+ | 4+ | 3+ |
| 2 | n.d. | n.d. | n.d. | n.d. | n.d. | n.d. | n.d. | n.d. |  | n.d. | n.d. | n.d. | n.d. | n.d. | n.d. | n.d. | n.d. |
| 5 | n.d. | n.d. | n.d. | n.d. | n.d. | n.d. | n.d. | n.d. |  | n.d. | n.d. | n.d. | n.d. | n.d. | 4055 | n.d. | n.d. |
| 10 | n.d. | n.d. | n.d. | n.d. | n.d. | n.d. | n.d. | n.d. |  | n.d. | n.d. | n.d. | n.d. | n.d. | 2756 | n.d. | n.d. |
| 15 | n.d. | n.d. | n.d. | n.d. | n.d. | n.d. | n.d. | n.d. |  | n.d. | n.d. | n.d. | n.d. | n.d. | n.d. | n.d. | n.d. |
| 20 | n.d. | n.d. | n.d. | 7690 | 6840 | n.d. | n.d. | n.d. |  | n.d. | n.d. | n.d. | 11870 | 10460 | 8773 | n.d. | n.d. |
| 25 | n.d. | n.d. | n.d. | 22700 | 15590 | 8252 | n.d. | n.d. |  | n.d. | n.d. | n.d. | 31550 | 25320 | 13450 | n.d. | n.d. |
| 30 | n.d. | n.d. | n.d. | 25920 | 18070 | 8619 | n.d. | n.d. |  | n.d. | n.d. | n.d. | 32620 | 30880 | 13930 | 3316 | n.d. |
| 35 | n.d. | n.d. | n.d. | 9468 | 10440 | 4989 | 1508 | n.d. |  | n.d. | n.d. | 200 | 12470 | 20380 | 8089 | 2789 | n.d. |
| 40 | n.d. | n.d. | 200 | 8917 | 14430 | 6452 | 2712 | n.d. |  | n.d. | n.d. | 200 | 13240 | 27250 | 9661 | 5346 | n.d. |
| 45 | n.d. | n.d. | 200 | 8033 | 17000 | 7529 | 4819 | n.d. |  | n.d. | n.d. | 200 | 13380 | 29590 | 10430 | 9609 | n.d. |
| 50 | n.d. | n.d. | 200 | 7539 | 17880 | 8434 | 8542 | n.d. |  | n.d. | n.d. | 200 | 13000 | 31520 | 11910 | 14650 | n.d. |
| 55 | n.d. | n.d. | 200 | 6908 | 18580 | 8454 | 10710 | n.d. |  | n.d. | n.d. | 200 | 12640 | 33190 | 12590 | 17680 | n.d. |
| 60 | n.d. | n.d. | 368 | 6383 | 17700 | 7938 | 11060 | n.d. |  | n.d. | n.d. | 445 | 12250 | 32580 | 13120 | 19420 | n.d. |
| 65 | n.d. | n.d. | 200 | 5172 | 15880 | 7826 | 10740 | n.d. |  | n.d. | n.d. | 460 | 11120 | 30700 | 13100 | 20100 | n.d. |
| 70 | n.d. | n.d. | 406 | 4579 | 13110 | 7778 | 9778 | n.d. |  | n.d. | n.d. | 474 | 8692 | 27330 | 13360 | 19550 | n.d. |
| 75 | n.d. | n.d. | 724 | 5864 | 14440 | 8759 | 11970 | n.d. |  | n.d. | n.d. | 863 | 11100 | 30740 | 16510 | 25920 | n.d. |
| 80 | n.d. | 200 | 595 | 4428 | 9134 | 6099 | 9575 | 200 |  | n.d. | n.d. | 832 | 9379 | 21530 | 12890 | 21180 | n.d. |
| 85 | n.d. | 200 | 687 | 3525 | 5620 | 4261 | 6729 | 200 |  | n.d. | 200 | 854 | 7283 | 13280 | 8301 | 16180 | 200 |
| 90 | n.d. | 200 | 611 | 2312 | 2777 | 2171 | 3798 | 200 |  | n.d. | 200 | 683 | 4717 | 6509 | 4978 | 9420 | 200 |
| 95 | n.d. | 200 | 443 | 1323 | 1395 | 1085 | 1773 | 200 |  | n.d. | 200 | 455 | 2957 | 2682 | 2563 | 4912 | 200 |
| 100 | n.d. | 200 | 505 | 930 | 742 | 666 | 882 | 200 |  | n.d. | 200 | 408 | 2098 | 1368 | 1360 | 2346 | 200 |
| 105 | n.d. | 200 | 501 | 712 | 603 | 393 | 368 | 200 |  | n.d. | 200 | 273 | 1213 | 619 | 605 | 969 | 200 |
| 110 | n.d. | n.d. | n.d. | n.d. | n.d. | n.d. | 169 | n.d. |  | n.d. | 200 | 251 | 845 | 383 | 348 | 372 | 200 |
| 115 | n.d. | n.d. | n.d. | n.d. | n.d. | n.d. | n.d. | n.d. |  | n.d. | n.d. | 251 | 595 | 274 | 245 | 186 | n.d. |
| 120 | n.d. | n.d. | n.d. | n.d. | n.d. | n.d. | n.d. | n.d. |  | n.d. | n.d. | 191 | 401 | 216 | 162 | 97 | n.d. |

1. calculated *m/z* value. Imputed intensity values which are necessary for optimized Gauss fits are printed in red and are equal to the background intensity at the given *m/z*. n.d.: value not determined / ion signal not present.
2. Ion signal intensity was not included in Gauss fit since this charge state represents an individual sdAbCSP1 protein conformation.

**Table S14.** Ion species, *m/z* values, charge states and intensities for sdAbCSP1 complexed with PfCSP-Cext at various collision cell voltage differences (continued).

measurement 2

| **∆**CV | PfCSP-Cext *m/z* and charge state | | | | | | |
| --- | --- | --- | --- | --- | --- | --- | --- |
|  | 1579.71^a)^ | 1777.15 | 2030.89 | 2369.22 | 2842.83 | 3553.35 | 4737.12^a)^ |
|  | 9+ | 8+ | 7+ | 6+ | 5+ | 4+ | 3+ |
| 2 | n.d. | n.d. | n.d. | n.d. | 3610 | n.d. | n.d. |
| 5 | n.d. | n.d. | n.d. | n.d. | 5147 | n.d. | n.d. |
| 10 | n.d. | n.d. | n.d. | n.d. | 3415 | n.d. | n.d. |
| 15 | n.d. | n.d. | n.d. | n.d. | 10950 | n.d. | n.d. |
| 20 | n.d. | n.d. | 4123 | 5240 | 16870 | n.d. | n.d. |
| 25 | n.d. | 200 | 16900 | 14030 | 27240 | 7884 | 200 |
| 30 | n.d. | 200 | 29120 | 20860 | 21980 | 9987 | 200 |
| 35 | n.d. | n.d. | 17330 | 16630 | 8513 | 6262 | n.d. |
| 40 | n.d. | 200 | 17150 | 30270 | 9672 | 8285 | 200 |
| 45 | n.d. | 200 | 17100 | 53780 | 10890 | 9312 | 200 |
| 50 | n.d. | 200 | 18580 | 78000 | 12050 | 9731 | 200 |
| 55 | n.d. | 414 | 20140 | 89090 | 12580 | 10290 | 200 |
| 60 | n.d. | 443 | 17750 | 87450 | 13060 | 10240 | 200 |
| 65 | n.d. | 496 | 15560 | 78480 | 13700 | 9495 | 200 |
| 70 | n.d. | 460 | 13100 | 67620 | 17260 | 8472 | 200 |
| 75 | n.d. | 920 | 19060 | 79060 | 30100 | 9835 | 200 |
| 80 | n.d. | 896 | 15660 | 59860 | 29370 | 6997 | 200 |
| 85 | n.d. | 1039 | 11530 | 44020 | 23770 | 4763 | 200 |
| 90 | n.d. | 944 | 6690 | 27540 | 15040 | 2566 | 200 |
| 95 | n.d. | 781 | 3413 | 14300 | 8233 | 1276 | 200 |
| 100 | n.d. | 666 | 1981 | 9634 | 4760 | 883 | 200 |
| 105 | n.d. | 436 | 1231 | 5136 | 2011 | 480 | 200 |
| 110 | n.d. | n.d. | 730 | 2633 | 1015 | 318 | 200 |
| 115 | n.d. | n.d. | n.d. | n.d. | n.d. | n.d. | n.d. |
| 120 | n.d. | n.d. | n.d. | n.d. | n.d. | n.d. | n.d. |

1. calculated *m/z* value. Imputed intensity values which are necessary for optimized Gauss fits are printed in red and are equal to the background intensity at the given *m/z*. n.d.: value not determined / ion signal not present.

**Table S14.** Ion species, *m/z* values, charge states and intensities for sdAbCSP1 complexed with PfCSP-Cext at various collision cell voltage differences (continued).

measurement 2

| **∆**CV | sdAbCSP1-PfCSP-Cext complex *m/z* and charge state ^a)^ | | | | | |  | pyroQ sdAbCSP1-PfCSP-Cext complex *m/z* and charge state ^a)^ | | | | | |
| --- | --- | --- | --- | --- | --- | --- | --- | --- | --- | --- | --- | --- | --- |
|  | 2503.88 | 2731.53 | 3004.66 | 3338.36 | 3755.31 | 4291.65 |  | 2502.46 | 2729.98 | 3002.82 | 3336.37 | 3753.26 | 4289.21 |
|  | 12+ | 11+ | 10+ | 9+ | 8+ | 7+ |  | 12+ | 11+ | 10+ | 9+ | 8+ | 7+ |
| 2 | 200 | 160100 | 430900 | 192000 | 6781 | 200 |  | 200 | 198100 | 535600 | 237100 | 8217 | 200 |
| 5 | 200 | 214100 | 578500 | 270900 | 12860 | 200 |  | 200 | 264400 | 714900 | 339600 | 15950 | 200 |
| 10 | 200 | 140500 | 386700 | 186600 | 8636 | 200 |  | 200 | 173700 | 473400 | 227400 | 10690 | 200 |
| 15 | 200 | 333700 | 962900 | 476400 | 22720 | 200 |  | 200 | 410400 | 1210000 | 598900 | 28200 | 200 |
| 20 | 200 | 348000 | 1070000 | 524000 | 23960 | 200 |  | 200 | 430300 | 1361000 | 677000 | 30450 | 200 |
| 25 | 200 | 344800 | 1218000 | 592700 | 27580 | 200 |  | 200 | 422400 | 1542000 | 772100 | 35650 | 200 |
| 30 | 200 | 177300 | 777200 | 383400 | 18570 | 200 |  | 200 | 220400 | 976900 | 502000 | 27400 | 200 |
| 35 | 200 | 36070 | 211600 | 109700 | 5135 | 200 |  | 200 | 47370 | 267000 | 141200 | 8518 | 200 |
| 40 | 200 | 19660 | 150400 | 90750 | 4680 | 200 |  | 200 | 25240 | 184000 | 118200 | 7758 | 200 |
| 45 | 200 | 10440 | 102000 | 82960 | 4471 | 200 |  | 200 | 12880 | 121000 | 110300 | 7107 | 200 |
| 50 | 200 | 4671 | 57020 | 76000 | 4081 | 200 |  | 200 | 4618 | 65780 | 102400 | 6651 | 200 |
| 55 | 200 | 2356 | 31770 | 69650 | 3994 | 200 |  | 200 | 1339 | 40240 | 99840 | 6627 | 200 |
| 60 | 200 | 1316 | 20120 | 61820 | 3602 | 200 |  | 200 | 916 | 27090 | 93120 | 6090 | 200 |
| 65 | 200 | 871 | 11960 | 51120 | 3346 | 200 |  | 200 | 200 | 16560 | 78430 | 5215 | 200 |
| 70 | 200 | 368 | 5197 | 35240 | 2916 | 200 |  | 200 | 351 | 6930 | 55120 | 4538 | 200 |
| 75 | 200 | 248 | 2683 | 24950 | 3563 | 200 |  | 200 | 200 | 3635 | 40130 | 5346 | 200 |
| 80 | 200 | 247 | 1037 | 8677 | 2882 | 200 |  | 200 | 200 | 1343 | 13760 | 4398 | 200 |
| 85 | 200 | 200 | 369 | 2820 | 2166 | 200 |  | 200 | 319 | 581 | 4053 | 3199 | 200 |
| 90 | 200 | n.d. | n.d. | n.d. | n.d. | 200 |  | 200 | 354 | 307 | 1203 | 2391 | 200 |
| 95 | 200 | n.d. | n.d. | n.d. | n.d. | 200 |  | 200 | 300 | 214 | 457 | 1635 | 200 |
| 100 | 200 | n.d. | n.d. | n.d. | n.d. | 200 |  | 200 | 296 | 227 | 326 | 1048 | 200 |
| 105 | 200 | 200 | 206 | 372 | 406 | 200 |  | 200 | 200 | 214 | 274 | 552 | 200 |
| 110 | n.d. | n.d. | n.d. | n.d. | n.d. | n.d. |  | n.d. | n.d. | n.d. | n.d. | n.d. | n.d. |
| 115 | n.d. | n.d. | n.d. | n.d. | n.d. | n.d. |  | n.d. | n.d. | n.d. | n.d. | n.d. | n.d. |
| 120 | n.d. | n.d. | n.d. | n.d. | n.d. | n.d. |  | n.d. | n.d. | n.d. | n.d. | n.d. | n.d. |

1. calculated *m/z* value. Imputed intensity values which are necessary for optimized Gauss fits are printed in red and are equal to the background intensity at the given *m/z*. n.d.: value not determined / ion signal not present.

**Table S15.** Ion species, *m/z* values, charge states and intensities for sdAbCSP1 complexed with PfCSP-Cext at various collision cell voltage differences.

measurement 3

| ∆ CV | sdAbCSP1 *m/z* and charge state ^a)^ | | | | | | | |  | pyroQ sdAbCSP1 *m/z* and charge state ^a)^ | | | | | | | |
| --- | --- | --- | --- | --- | --- | --- | --- | --- | --- | --- | --- | --- | --- | --- | --- | --- | --- |
|  | 1583.62 | 1759.46 | 1979.18 | 2261.84 | 2638.70 | 3166.08 | 3957.43 ^b)^ | 5276.39 |  | 1581.91 | 1757.57 | 1977.10 | 2259.39 | 2635.79 | 3162.74 | 3952.85 ^b)^ | 5270.71 |
|  | 10+ | 9+ | 8+ | 7+ | 6+ | 5+ | 4+ | 3+ |  | 10+ | 9+ | 8+ | 7+ | 6+ | 5+ | 4+ | 3+ |
| 2 | n.d. | n.d. | n.d. | n.d. | n.d. | n.d. | n.d. | n.d. |  | n.d. | n.d. | n.d. | n.d. | n.d. | n.d. | n.d. | n.d. |
| 5 | n.d. | n.d. | n.d. | n.d. | n.d. | n.d. | n.d. | n.d. |  | n.d. | n.d. | n.d. | n.d. | n.d. | n.d. | n.d. | n.d. |
| 10 | n.d. | n.d. | n.d. | n.d. | n.d. | n.d. | n.d. | n.d. |  | n.d. | n.d. | n.d. | n.d. | n.d. | n.d. | n.d. | n.d. |
| 15 | n.d. | n.d. | n.d. | n.d. | n.d. | n.d. | n.d. | n.d. |  | n.d. | n.d. | n.d. | n.d. | n.d. | n.d. | n.d. | n.d. |
| 20 | n.d. | n.d. | n.d. | n.d. | n.d. | n.d. | n.d. | n.d. |  | n.d. | n.d. | n.d. | n.d. | n.d. | n.d. | n.d. | n.d. |
| 25 | n.d. | n.d. | n.d. | n.d. | n.d. | n.d. | n.d. | n.d. |  | n.d. | n.d. | n.d. | n.d. | n.d. | n.d. | n.d. | n.d. |
| 30 | n.d. | 200 | 200 | n.d. | 3069 | 2371 | 200 | n.d. |  | n.d. | n.d. | n.d. | n.d. | n.d. | n.d. | n.d. | n.d. |
| 35 | n.d. | n.d. | 200 | 4314 | 6921 | 3816 | 200 | n.d. |  | n.d. | n.d. | 200 | 8408 | 19230 | 7595 | 200 | n.d. |
| 40 | n.d. | n.d. | 200 | 5600 | 14030 | 5775 | 200 | n.d. |  | n.d. | n.d. | 200 | 11550 | 35190 | 11180 | 200 | n.d. |
| 45 | n.d. | n.d. | 200 | 5969 | 18390 | 7376 | 200 | n.d. |  | n.d. | n.d. | 200 | 13490 | 43680 | 14720 | 200 | n.d. |
| 50 | n.d. | n.d. | 200 | 6244 | 22100 | 8766 | 200 | n.d. |  | n.d. | n.d. | 200 | 13990 | 51100 | 17500 | 200 | n.d. |
| 55 | n.d. | n.d. | 200 | 6283 | 24420 | 10110 | 200 | n.d. |  | n.d. | n.d. | 200 | 14250 | 55990 | 18890 | 200 | n.d. |
| 60 | n.d. | n.d. | 200 | 5882 | 23390 | 10870 | 200 | n.d. |  | n.d. | n.d. | 200 | 13130 | 54110 | 21990 | 200 | n.d. |
| 65 | n.d. | n.d. | 200 | 5063 | 22250 | 11880 | 200 | n.d. |  | n.d. | n.d. | 200 | 11250 | 52530 | 26090 | 200 | n.d. |
| 70 | n.d. | n.d. | 409 | 4402 | 19640 | 12950 | 200 | n.d. |  | n.d. | n.d. | 200 | 10530 | 46680 | 28460 | 200 | n.d. |
| 75 | n.d. | n.d. | 399 | 3785 | 15460 | 12270 | 200 | n.d. |  | n.d. | n.d. | 666 | 9023 | 39130 | 27000 | 200 | n.d. |
| 80 | n.d. | n.d. | 423 | 3033 | 10020 | 9756 | 200 | n.d. |  | n.d. | n.d. | 684 | 7342 | 28090 | 21920 | 200 | n.d. |
| 85 | n.d. | 200 | 429 | 2532 | 6683 | 6862 | 200 | n.d. |  | n.d. | 200 | 626 | 6128 | 17560 | n.d. | 200 | n.d. |
| 90 | n.d. | 200 | 394 | 1937 | 3456 | 3923 | 200 | n.d. |  | n.d. | 200 | 471 | 5270 | 9106 | n.d. | 200 | n.d. |
| 95 | n.d. | 200 | 623 | 1376 | 1923 | 2281 | 200 | n.d. |  | n.d. | 200 | 200 | 3828 | 4215 | n.d. | 200 | n.d. |
| 100 | 200 | 200 | 690 | 1105 | 1075 | 1207 | 200 | 200 |  | n.d. | 200 | 368 | 2620 | 1918 | n.d. | 200 | n.d. |
| 105 | 200 | 200 | 786 | 948 | 886 | 785 | 200 | 200 |  | n.d. | 200 | 406 | 1850 | 1091 | n.d. | 200 | n.d. |
| 110 | 200 | 200 | n.d. | 672 | 690 | 627 | 200 | 200 |  | n.d. | 200 | 358 | 1144 | 722 | n.d. | 200 | n.d. |
| 115 | 200 | 200 | n.d. | 689 | 576 | 505 | 200 | 200 |  | n.d. | 200 | 478 | 880 | 582 | n.d. | 200 | n.d. |
| 120 | n.d. | n.d. | n.d. | n.d. | n.d. | n.d. | n.d. | n.d. |  | n.d. | 200 | 335 | 615 | 440 | n.d. | 200 | n.d. |

1. calculated *m/z* value. Imputed intensity values which are necessary for optimized Gauss fits are printed in red and are equal to the background intensity at the given *m/z*. n.d.: value not determined / ion signal not present.
2. Ion signal intensity was not included in Gauss fit since this charge state represents an individual sdAbCSP1 protein conformation.

**Table S15.** Ion species, *m/z* values, charge states and intensities for sdAbCSP1 complexed with PfCSP-Cext at various collision cell voltage differences (continued).

measurement 3

| **∆**CV | PfCSP-Cext *m/z* and charge state ^a)^ | | | | | | |
| --- | --- | --- | --- | --- | --- | --- | --- |
|  |  |  |  |  |  |  |  |
|  | 1579.71 | 1777.15 | 2030.89 | 2369.22 | 2842.83 | 3553.35 | 4737.12 |
|  | 9+ | 8+ | 7+ | 6+ | 5+ | 4+ | 3+ |
| 2 | n.d. | n.d. | n.d. | n.d. | n.d. | n.d. | n.d. |
| 5 | n.d. | n.d. | n.d. | n.d. | n.d. | n.d. | n.d. |
| 10 | n.d. | n.d. | n.d. | n.d. | n.d. | n.d. | n.d. |
| 15 | n.d. | n.d. | n.d. | n.d. | n.d. | n.d. | n.d. |
| 20 | n.d. | n.d. | n.d. | n.d. | n.d. | n.d. | n.d. |
| 25 | n.d. | 200 | n.d. | 1642 | 3223 | 974 | 200 |
| 30 | n.d. | 200 | n.d. | 3776 | 4509 | 1921 | 200 |
| 35 | n.d. | n.d. | n.d. | n.d. | n.d. | n.d. | n.d. |
| 40 | n.d. | 200 | 11650 | 21960 | n.d. | 8376 | 200 |
| 45 | n.d. | 200 | 12820 | 58880 | n.d. | 11060 | 200 |
| 50 | n.d. | 200 | 16360 | 104000 | n.d. | 12830 | 200 |
| 55 | n.d. | 200 | 17890 | 126600 | n.d. | 13960 | 200 |
| 60 | n.d. | 200 | 16080 | 123400 | n.d. | 14280 | 200 |
| 65 | n.d. | 200 | 14140 | 114500 | n.d. | 15250 | 200 |
| 70 | n.d. | 200 | 13390 | 99890 | 40720 | n.d. | 200 |
| 75 | n.d. | 296 | 12160 | 82820 | 56130 | 13220 | 200 |
| 80 | n.d. | 431 | 9580 | 65370 | 59810 | 10370 | 200 |
| 85 | n.d. | 485 | 7475 | 48760 | 50110 | 7271 | 200 |
| 90 | n.d. | 617 | 5162 | 34190 | 34720 | 4629 | 200 |
| 95 | n.d. | 839 | 3075 | 23070 | 21490 | 3029 | 200 |
| 100 | n.d. | 868 | 1935 | 15140 | 10320 | 1758 | 200 |
| 105 | n.d. | 864 | 1334 | 9079 | 4850 | 1032 | 200 |
| 110 | n.d. | n.d. | 1009 | 4717 | 1932 | 762 | n.d. |
| 115 | n.d. | n.d. | 892 | 2335 | 901 | 564 | n.d. |
| 120 | n.d. | n.d. | n.d. | n.d. | n.d. | n.d. | n.d. |

1. calculated *m/z* value. Imputed intensity values which are necessary for optimized Gauss fits are printed in red and are equal to the background intensity at the given *m/z*. n.d.: value not determined / ion signal not present.

**Table S15.** Ion species, *m/z* values, charge states and intensities for sdAbCSP1 complexed with PfCSP-Cext at various collision cell voltage differences (continued).

measurement 3

| **∆**CV | sdAbCSP1-PfCSP-Cext complex *m/z* and charge state ^a)^ | | | | | |  | pyroQ sdAbCSP1-PfCSP-Cext complex *m/z* and charge state ^a)^ | | | | | |
| --- | --- | --- | --- | --- | --- | --- | --- | --- | --- | --- | --- | --- | --- |
|  | 2503.88 | 2731.53 | 3004.66 | 3338.36 | 3755.31 | 4291.65 |  | 2502.46 | 2729.98 | 3002.82 | 3336.37 | 3753.26 | 4289.21 |
|  | 12+ | 11+ | 10+ | 9+ | 8+ | 7+ |  | 12+ | 11+ | 10+ | 9+ | 8+ | 7+ |
| 2 | 200 | 9788 | 41900 | 31000 | 1722 | 200 |  | 200 | 14370 | 60100 | 44810 | 2372 | 200 |
| 5 | 200 | 16730 | 71700 | 52670 | 2900 | 200 |  | 200 | 24520 | 102400 | 75100 | 4183 | 200 |
| 10 | 200 | 22680 | 94110 | 70430 | 3665 | 200 |  | 200 | 33030 | 135900 | 99500 | 5412 | 200 |
| 15 | 200 | 29450 | 125600 | 93490 | 4947 | 200 |  | 200 | 42670 | 181700 | 132500 | 7334 | 200 |
| 20 | 200 | 31460 | 151300 | 114700 | 5758 | 200 |  | 200 | 44960 | 215000 | 161000 | 8461 | 200 |
| 25 | 200 | 30140 | 171200 | 129900 | 6998 | 200 |  | 200 | 42920 | 244900 | 188500 | 10770 | 200 |
| 30 | 200 | 24310 | 178800 | 146400 | 9628 | 200 |  | 200 | 34830 | 255500 | 210200 | 14870 | 200 |
| 35 | 200 | 16530 | 168000 | 152000 | 12040 | 200 |  | 200 | 23590 | 235600 | 221000 | 19200 | 200 |
| 40 | 200 | 11420 | 153400 | 161500 | 13450 | 200 |  | 200 | 15810 | 208500 | 230300 | 21400 | 200 |
| 45 | 200 | 7015 | 116500 | 161700 | 13460 | 200 |  | 200 | 9482 | 152400 | 234600 | 23340 | 200 |
| 50 | 200 | 3021 | 65510 | 157600 | 14070 | 200 |  | 200 | 3460 | 87820 | 241300 | 23930 | 200 |
| 55 | 200 | n.d. | 38600 | 152600 | 14710 | 200 |  | 200 | 1299 | 52700 | 242400 | 24520 | 200 |
| 60 | 200 | n.d. | 25580 | 143700 | 15140 | 200 |  | 200 | 200 | 34330 | 232500 | 25260 | 200 |
| 65 | 200 | 620 | 14710 | 124500 | 15720 | 200 |  | 200 | 200 | 20030 | 202700 | 24230 | 200 |
| 70 | 200 | n.d. | 6333 | 82640 | 12830 | 200 |  | 200 | 200 | 8422 | 132000 | 20450 | 200 |
| 75 | 200 | 317 | n.d. | 39660 | 10880 | 200 |  | 200 | 200 | 3820 | 63000 | 16770 | 200 |
| 80 | 200 | n.d. | 1166 | 13330 | 9462 | 200 |  | 200 | 399 | 1456 | 21620 | 15410 | 200 |
| 85 | 200 | 458 | 476 | n.d. | 7941 | 200 |  | 200 | 493 | 716 | 8012 | 12560 | 200 |
| 90 | 200 | 503 | 352 | 1510 | 5605 | 200 |  | 200 | 563 | 200 | 2158 | 9666 | 200 |
| 95 | n.d. | n.d. | n.d. | n.d. | n.d. | n.d. |  | 200 | 610 | 456 | 1028 | 6831 | 200 |
| 100 | n.d. | n.d. | n.d. | n.d. | n.d. | n.d. |  | 200 | n.d. | 426 | 745 | 3702 | 200 |
| 105 | 200 | n.d. | 427 | 1176 | 1057 | 200 |  | n.d. | n.d. | n.d. | n.d. | n.d. | n.d. |
| 110 | n.d. | n.d. | n.d. | n.d. | n.d. | n.d. |  | n.d. | n.d. | n.d. | n.d. | n.d. | n.d. |
| 115 | n.d. | n.d. | n.d. | n.d. | n.d. | n.d. |  | n.d. | n.d. | n.d. | n.d. | n.d. | n.d. |
| 120 | n.d. | n.d. | n.d. | n.d. | n.d. | n.d. |  | n.d. | n.d. | n.d. | n.d. | n.d. | n.d. |

1. calculated *m/z* value. Imputed intensity values which are necessary for optimized Gauss fits are printed in red and are equal to the background intensity at the given *m/z*. n.d.: value not determined / ion signal not present.

**Table S16:** Apex heights of Gaussian fits of educts and products ion signals upon gas phase dissociation of sdAbCSP1 – PfCSP-Cext complex ^a)^.

measurement 1

| **∆CV** | **products** | | | | |  | **educts** | |
| --- | --- | --- | --- | --- | --- | --- | --- | --- |
|  | **sdAbCSP1** | **sdAbCSP1‘** | **pQ-sdAbCSP1** | **pQ-sdAbCSP1** | **PfCSP-Cext** |  | **sdAbCSP1- PfCSP-Cext** | **pQ-sdAbCSP1- PfCSP-Cext** |
| 2 | n.d. | n.d. | n.d. | n.d. | n.d. |  | 242154 | 312213 |
| 5 | n.d. | n.d. | n.d. | n.d. | n.d. |  | 436345 | 545890 |
| 10 | n.d. | n.d. | n.d. | n.d. | n.d. |  | 469854 | 578353 |
| 15 | n.d. | n.d. | n.d. | n.d. | n.d. |  | 250657 | 294308 |
| 20 | n.d. | n.d. | n.d. | n.d. | 11557 |  | 577829 | 720388 |
| 25 | n.d. | n.d. | n.d. | n.d. | 5460 |  | 250939 | 289662 |
| 30 | n.d. | 2440 | n.d. | 1290 | n.d. |  | 227309 | 259483 |
| 35 | n.d. | 12950 | 37158 | 4421 | n.d. |  | 356370 | 431258 |
| 40 | 76204 | 17630 | 145516 | 26250 | 169662 |  | 892762 | 1135520 |
| 45 | 64402 | 50090 | 114957 | 32180 | 208169 |  | 468988 | 571826 |
| 50 | 120832 | 54770 | 231239 | 92470 | 565693 |  | 687380 | 989852 |
| 55 | 111118 | 64640 | 210882 | 100400 | 586294 |  | 500556 | 749042 |
| 60 | 118087 | 63850 | 229352 | 121500 | 661198 |  | 444564 | 731198 |
| 65 | 110579 | 46720 | 222488 | 126600 | 598707 |  | 364132 | 596605 |
| 70 | 71270 | 53110 | 149402 | 97140 | 386343 |  | 192993 | 310641 |
| 75 | 72177 | 45640 | 151927 | 120400 | 430850 |  | 131958 | 217242 |
| 80 | 49273 | 32940 | 114384 | 108900 | 367067 |  | 51467 | 87816 |
| 85 | 44240 | 20940 | 107880 | 84990 | 291083 |  | 18822 | 32999 |
| 90 | 26497 | 10560 | 73072 | 56420 | 214921 |  | 14381 | 23846 |
| 95 | 15366 | 5009 | 38901 | 30710 | 142464 |  | n.d. | 16661 |
| 100 | 8926 | 2085 | 24871 | 14810 | 91933 |  | n.d. | n.d. |
| 105 | n.d. | n.d. | 14150 | 5922 | 50235 |  | n.d. | n.d. |
| 110 | n.d. | n.d. | 10089 | 2325 | 28263 |  | n.d. | n.d. |
| 115 | n.d. | 430 | 5247 | 1013 | 13401 |  | n.d. | n.d. |
| 120 | n.d. | n.d. | 3121 | 657 | 6385 |  | 242154 | 312213 |

^a)^ n.d.: not determined

**Table S16:** Apex heights of Gaussian fits of educts and products ion signals upon gas phase dissociation of sdAbCSP1 – PfCSP-Cext complex ^a)^.

(continued)

measurement 2

| **∆CV** | **products** | | | | |  | **educts** | |
| --- | --- | --- | --- | --- | --- | --- | --- | --- |
|  | **sdAbCSP1** | **sdAbCSP1** | **pQ-sdAbCSP1** | **pQ-sdAbCSP1** | **PfCSP-Cext** |  | **sdAbCSP1 - PfCSP-Cext** | **pQ-sdAbCSP1 - PfCSP-Cext** |
| 2 | n.d. | n.d. | n.d. | n.d. | n.d. |  | 438421 | 544787 |
| 5 | n.d. | n.d. | n.d. | n.d. | n.d. |  | 583180 | 723080 |
| 10 | n.d. | n.d. | n.d. | n.d. | n.d. |  | 392877 | 480219 |
| 15 | n.d. | n.d. | n.d. | n.d. | n.d. |  | 983917 | 1237300 |
| 20 | n.d. | n.d. | n.d. | n.d. | n.d. |  | 1094140 | 1360650 |
| 25 | n.d. | n.d. | n.d. | n.d. | 27973 |  | 1249580 | 1561410 |
| 30 | n.d. | n.d. | n.d. | 3316 | 25920 |  | 806412 | 1006910 |
| 35 | n.d. | 1508 | 22240 | 2789 | n.d. |  | 224008 | 282151 |
| 40 | 15522 | 2712 | 27667 | 5346 | 34996 |  | 168905 | 208094 |
| 45 | 17681 | 4819 | 29742 | 9609 | 58681 |  | 127650 | 157635 |
| 50 | 18595 | 8542 | 31453 | 14650 | 87747 |  | 95700 | 124248 |
| 55 | 19025 | 10710 | 33374 | 17680 | 100357 |  | 80959 | 112127 |
| 60 | 17943 | 11060 | 32952 | 19420 | 100908 |  | 68258 | 100150 |
| 65 | 16287 | 10740 | 31171 | 20100 | 90612 |  | 53632 | 81533 |
| 70 | 13711 | 9778 | 27991 | 19550 | 67364 |  | 35332 | 55136 |
| 75 | 15105 | 11970 | 31825 | 25920 | 79372 |  | 24798 | 40841 |
| 80 | 9464 | 9575 | 22898 | 21180 | 59038 |  | 8907 | 14399 |
| 85 | 5958 | 6729 | 13809 | 16180 | 43989 |  | 3364 | 4843 |
| 90 | 2993 | 3798 | 7161 | 9420 | 27733 |  | n.d. | 3671 |
| 95 | 1917 | 1773 | 6377 | 4912 | 14475 |  | n.d. | 1479 |
| 100 | 951 | 882 | 3469 | 2346 | 9573 |  | n.d. | 978 |
| 105 | 553 | 368 | 1943 | 969 | 4827 |  | 539 | 483 |
| 110 | n.d. | 169 | 1036 | 372 | 2477 |  | n.d. | n.d. |
| 115 | n.d. | n.d. | n.d. | 186 | n.d. |  | n.d. | n.d. |
| 120 | n.d. | n.d. | n.d. | 97 | n.d. |  | n.d. | n.d. |

^a)^ n.d.: not determined

**Table S16:** Apex heights of Gaussian fits of educts and products ion signals upon gas phase dissociation of sdAbCSP1 – PfCSP-Cext complex ^a)^.

(continued)

measurement 3

| **∆CV** | **products** | | | | |  | **edducts** | |
| --- | --- | --- | --- | --- | --- | --- | --- | --- |
|  | **sdAbCSP1** | **sdAbCSP1‘** | **pQ-sdAbCSP1** | **pQ-sdAbCSP1** | **PfCSP-Cext** |  | **sdAbCSP1 - PfCSP-Cext** | **pQ-sdAbCSP1 - PfCSP-Cext** |
| 2 | n.d. | n.d. | n.d. | n.d. | n.d. |  | 46635 | 67666 |
| 5 | n.d. | n.d. | n.d. | n.d. | n.d. |  | 79678 | 114614 |
| 10 | n.d. | n.d. | n.d. | n.d. | n.d. |  | 105088 | 151930 |
| 15 | n.d. | n.d. | n.d. | n.d. | n.d. |  | 140390 | 203409 |
| 20 | n.d. | n.d. | n.d. | n.d. | n.d. |  | 172710 | 245555 |
| 25 | n.d. | n.d. | n.d. | n.d. | 3134 |  | 198669 | 286666 |
| 30 | 4454 | n.d. | n.d. | n.d. | 4560 |  | 218112 | 312085 |
| 35 | 8143 | n.d. | 19257 | 1469 | n.d. |  | 217735 | 309309 |
| 40 | 14169 | 1531 | 34648 | 4324 | 26997 |  | 216837 | 300071 |
| 45 | 18488 | 4508 | 43558 | 10680 | 69819 |  | 197540 | 275006 |
| 50 | 22222 | 9400 | 51146 | 20490 | 130021 |  | 160750 | 257073 |
| 55 | 24693 | 12670 | 56057 | 28440 | 158363 |  | 153655 | 247708 |
| 60 | 23961 | 14260 | 54954 | 30690 | 162471 |  | 144752 | 237787 |
| 65 | 23386 | 15040 | 54985 | 34770 | 145073 |  | 127146 | 207128 |
| 70 | 21583 | 16340 | 50740 | 39270 | 105408 |  | 83036 | 134810 |
| 75 | 17729 | 17090 | 43673 | 42410 | 90444 |  | 39735 | 67510 |
| 80 | 12235 | 15360 | 32064 | 40490 | 79937 |  | 16443 | 27313 |
| 85 | 6414 | 11910 | 17269 | 32590 | 63771 |  | 10962 | 16723 |
| 90 | 3316 | 7358 | 8981 | 21340 | 44682 |  | 5595 | 9887 |
| 95 | 1769 | 3798 | 6965 | 11870 | 28869 |  | n.d. | 6868 |
| 100 | 1038 | 1679 | 3040 | 5379 | 17006 |  | n.d. | 3621 |
| 105 | 873 | 838 | 1830 | 2128 | 9530 |  | 1165 | n.d. |
| 110 | 593 | 422 | 1021 | 911 | 4427 |  | n.d. | n.d. |
| 115 | 520 | 240 | 700 | 463 | 1959 |  | n.d. | n.d. |
| 120 | n.d. | n.d. | 430 | 226 | n.d. |  | n.d. | n.d. |

^a)^ n.d.: not determined

**Table S17.** Course characteristics of gas phase dissociation of the sdAbCSP1 – PfCSP-Cext complex.

| complex (educt) | initial [%] ^a,b)^ | final [%] ^a,c)^ | ∆CV_50_ [V] | dx [V] | slope [%/V] | R^2^ |
| --- | --- | --- | --- | --- | --- | --- |

| sdAbCSP1 - PfCSP-Cext | 104.81 | -2.30 | 60.53 | 15.18 | -1.76 | 0.991 |
| --- | --- | --- | --- | --- | --- | --- |

^a)^ averaged amounts at corresponding ∆CV from three measurement series (Boltzmann fits)

^b)^ immune complex amount at lowest applied ∆CV (0 V)

^c)^ immune complex amount at highest applied ∆CV (120 V)

**Table S18.** Thermodynamic *in-solution* values for sdAbCSP1 – PfCSP-Cext complex formation.

| complex | N ^a)^ [Ø] | K_D_ [M] | ΔG [kJ/mol] | ΔH [kJ/mol] | TΔS ^b)^ [kJ/mol] |
| --- | --- | --- | --- | --- | --- |
| sdAbCSP1 – PfCSP-Cext | 0.90 ± 0.12 | 5 x 10^-10^ ± 2 x 10^-10^ | -48.76 ± 1.68 | -34.92 ± 3.10 | 13.84 ± 4.54 |

^a)^ unitless number ^b)^ T_amb_: 298 K
